# New Fluorescent Synthetic Retinoids as Potential RAR Agonists with Anticancer, Molecular Docking and ADME Assessments

**DOI:** 10.1007/s10895-025-04343-6

**Published:** 2025-05-23

**Authors:** Esraa Ibrahim, Yara E. Mansour, Sameh Soror, Hesham Haffez

**Affiliations:** 1 Biochemistry and Molecular Biology Department, Faculty of Pharmacy, Helwan University, Cairo, 11795 Egypt; 2 Center of Scientific Excellence “Helwan Structural Biology Research, (HSBR)”, Helwan University, Cairo, 11795 Egypt; 3 Pharmaceutical Organic Chemistry Department, Faculty of Pharmacy, Helwan University, Cairo, 11795 Egypt

**Keywords:** Anticancer, Caco-2, Cell cycle arrest, Fluorescent, RARs

## Abstract

Natural and synthetic retinoids are derivatives of vitamin A that mediate different transcriptional activities. Synthesis of fluorescent probes can be a useful tool in cellular imaging and understanding different regulatory signaling pathways. In this study, new fluorescent synthetic retinoid analogues (**3a**, **3b**, **4a**, and **4b**) were prepared and biologically screened against six different cancer and normal cell lines for assessment of their anticancer and fluorescent activities. A combination of biological assays, such as MTT, flow cytometry, gene and protein expression analysis, DNA fragmentation, and ATPase activity assays, as well as *in-silico* and ADME studies, was performed. All new compounds showed anti-proliferative activity (2.09–132.70 µM) against different cancer cell types with minimal cytotoxicity (SI > 1), and the Caco-2 cancer cell line was selected for further in vitro investigation. All compounds showed cell cycle arrest at the SubG_0_-G_1_ phase with induction of early apoptosis and necrosis. Compound **4b** showed a significant apoptotic effect by overexpression of Caspase-3 and Cyt-c genes, followed by compound **3a**. All compounds showed anti-inflammatory activity by significantly down-regulating the IL-10 pro-inflammatory marker, while compounds **3b**, **4a**, and **4b** specifically down-regulated IL-6. Retinoic acid receptors (RARs) were suggested as molecular targets confirmed by their overexpression of both gene and protein levels with molecular docking and molecular dynamic simulation studies. All fluorescent compounds showed intracellular fluorescent emission spectra with intracellular lipophilic properties. The new fluorescent synthetic retinoids showed dual activities as anticancer agents with fluorescence properties and can be used as useful probes in a variety of cellular imaging investigations.

**Supplementary Information:**

The online version contains supplementary material available at 10.1007/s10895-025-04343-6.

## Introduction

**Fig. 1:**
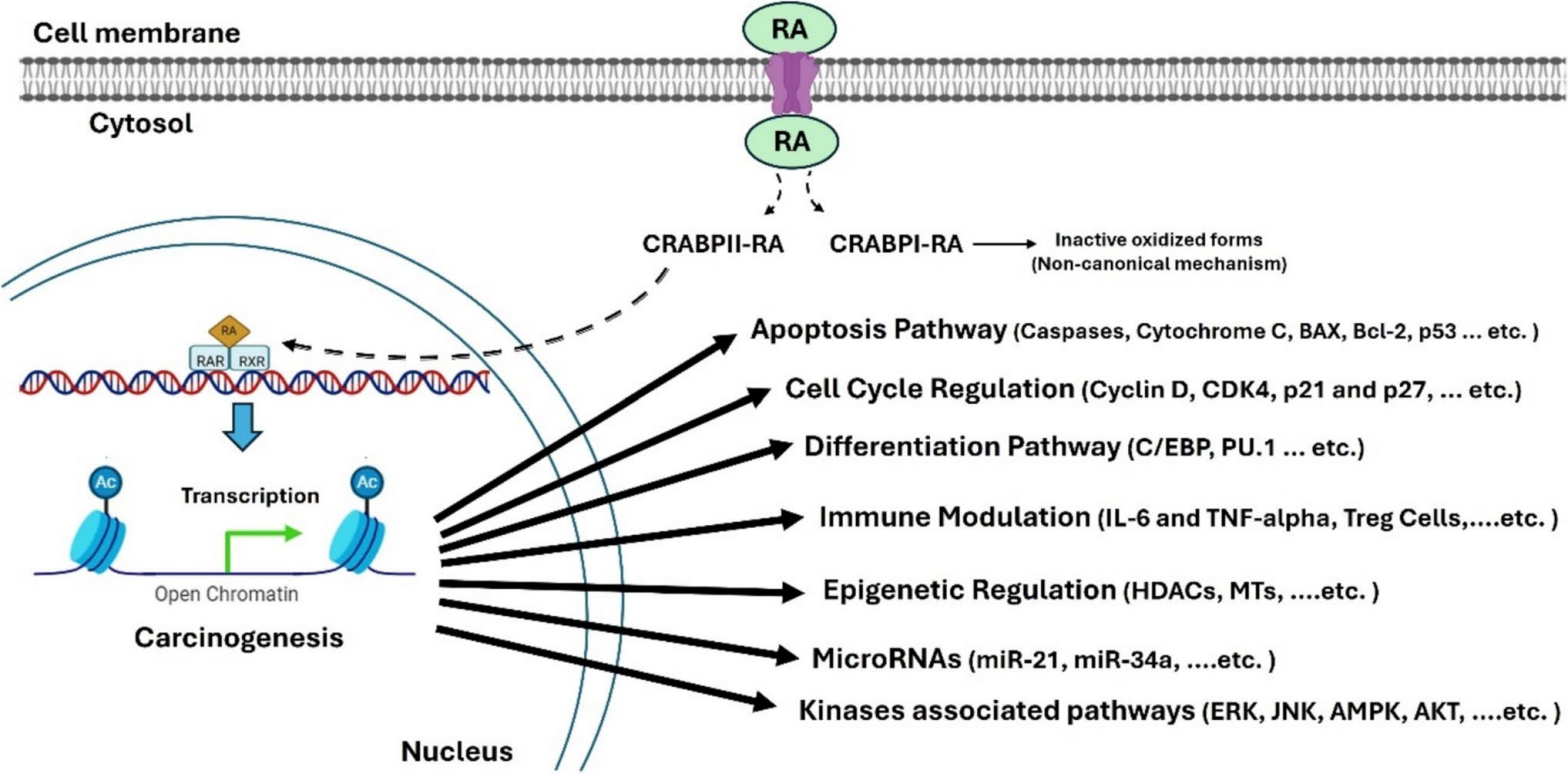
Schematic representation of the RARs'and retinoids'implication as anticancer therapy

**Fig. 2:**
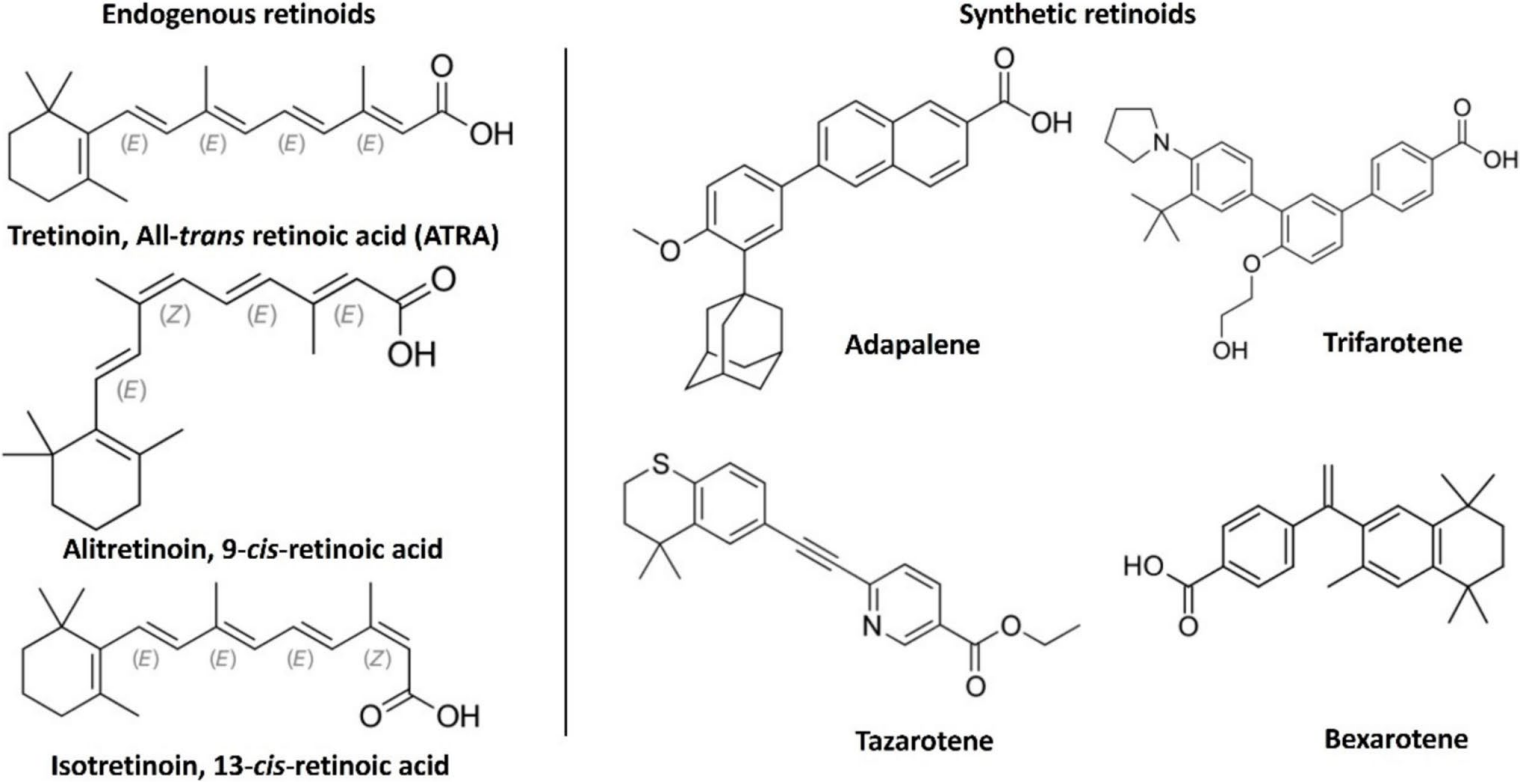
The chemical structures of endogenous and some synthetic retinoids (color is not required in print)

The multistep process known as carcinogenesis transforms normal cell phenotypes into malignant equivalents that can invade and spread, giving rise to clinically evident malignancies [1]. Significant advancements in our knowledge have been made throughout the past few decades about the physiological, genetic, environmental, and biochemical bases of cancer etiology [2]. Retinoids are a group of vitamin A analogs that exhibit a remarkable capacity to foster the development and differentiation of a range of mammalian epithelial tissue [3]. The prevention of experimental carcinogenesis in both *in-vivo* and *in-vitro* animal models is significantly supported by retinoids [4]. Additionally, the clinical remission of some malignancies has shown some progression, notably with the treatment of acute promelocytic leukemia (AML) [5] due to the fact that retinoids can block or reverse the altered phenotype of cancer cells in *in-vitro* experimental carcinogenesis [6]. However, the acquired cancer resistance has made it necessary to search for novel synthetic retinoids that could overcome the retinoids’ toxicity and resistance issues [7]. Retinoids have been shown to be effective in treating certain premalignant lesions and in lowering the occurrence of second primary tumors in patients who had previously developed head and neck, lung, or liver cancer [8]. However, it is still unknown whether retinoids can prevent primary cancers at these locations. Retinoids and rexinoids have undergone extensive testing in numerous preclinical studies and for the treatment of malignancies, as previously discussed, due to the discovery that retinoids activate nuclear retinoid receptors (RAR-α, RAR-β, RAR-γ, and RXRs) [9]. These nuclear receptors bind to their cognate active natural substrates, all-trans-retinoic acid (ATRA; RA) and its natural isomers 9-cis-RA and 13-cis-RA, and form heterodimers that act as transcription activators for specific target genes by modulating their gene expression pattern [10]. Once RA enters the cells, it binds to the cellular retinoic acid-binding protein (CRABP) 1 and 2 proteins, which perform a variety of functions. CRABP1 transports RA for oxidative metabolism by cytochrome P (CYP-450) enzymes, while CRABP2 translocates RA from the cytosol to the nucleus for the delivery of the ligand to nuclear RARs for transcriptional activity [11]. Ligand activation creates a hydrophobic surface for coactivators with the removal of co-repressors consequently. In cancer cells, activation of RARs by retinoid agonists promotes the transcription of different molecular mediators that can activate several pathways, including apoptosis, cell cycle arrest, differentiation, modulation of key inflammatory mediators of the immune system, activation of kinase pathways, and epigenetic modifications on the level of enzymes and miRNA (Fig. 1) [12, 13]. Therefore, RARs are important molecular targets for cancer therapy. However, the developed resistance and metastasis and the lack of information about the retinoids'localization and mechanism of action are still under scientific investigation [14]. Synthesis of novel retinoids has traditionally been based on data already available, which shows that substituent alterations may be applied to the ATRA molecule's nonpolar trimethyl cyclohexene ring, the opposite free carbonyl terminal, or both ends as shown in some examples (Fig. 2) [15]. Many of them have been discovered to potentially have cancer-preventing properties [16] with high binding affinity to the cytoplasmic retinoid receptor CRBPs. Previous studies on a series of conformationally restricted synthetic retinoids known as EC-synthetic retinoids showed biological activity in both the level of stem cell neuro-differentiation [17–19] as well as the retinoid suppression of tumor growth activity through the use of different *in-vitro* bioassays [20–22].

Additionally, researchers have been exerting remarkable effort to create"theranostic"molecules that might not only treat tumors but also identify or track their response to therapy [23]. Theranostic now relies heavily on nanotechnology [24] or conjugation with tumor-specific ligands [25], contrast agents [26], and anticancer medications [27]. These techniques have been demonstrated to be effective to some extent; however, there are some disagreements regarding their selectivity and specificity [23]. Researchers'attention has recently been drawn to small molecule-based fluorophores with distinctive intrinsic targeting, since these theranostic agents not only answer the targeting problem but also offer advantages for potential large-scale use [28]. For example, multifunctional heptamethine dyes have been created for tumor imaging and treatment that can be selectively enhanced in the mitochondria of different human tumor xenografts [23].

However, they still have certain undesirable side effects because of their poor water solubility and subpar anticancer efficacy. Hence, the best strategy currently is using an internal resonance structure with fluorescent properties that can benefit from imaging property without interference with biological activity. Therefore, the focus of our study was to evaluate the potential anticancer activity of new scaffolds of conformationally restricted synthetic fluorescent retinoids. The current study used different biological assays to determine their anti-proliferative activity and to validate the methods by investigating whether there are significant positive linear correlations between two or more bioassay data. Moreover, we developed these molecules with an expected fluorescent-based probe, which can be selectively enriched in the cytoplasm of cancer cells, thus promoting a novel theranostic agent for the diagnosis and treatment of cancer.

## Experimental

### Chemistry Synthesis

The starting ingredients, solvents, and reagents were procured from Sigma-Aldrich Fine Chemicals (St. Louis, MO, USA) and Merck KgaA (Darmstadt, Germany). The advancement of reactions was tracked using thin-layer chromatography (TLC) performed on silica gel-precoated aluminum sheets (type 60, F 254, Merck KgaA, Darmstadt, Germany). The spots were observed using a UV light lamp with a maximum wavelength of 254 nm. The eluent employed was a combination of hexane and ethyl acetate. NMR spectra were obtained using the Varian Mercury-300 NMR Spectrometer and the Bruker NMR spectrometer (Bruker BioSpin GmbH, Rheinstetten, Germany). The ^1^HNMR spectra were conducted at either 300 or 400 MHz, while the ^13^CNMR spectra were conducted at either 75 or 100 MHz. These experiments were performed using deuterated dimethyl sulfoxide (DMSO-d6) as the solvent. Chemical shifts are quantified using δ values (parts per million) with respect to TMS (tetramethylsilane). The values of all coupling constants (*J*) are provided in hertz (Hz). The acronyms used are “s” for singlet, “d” for doublet, “t” for triplet, “m” for multiplet, and “dd” for doublet-doublet. The electrospray ionization mass (ESI–MS) was acquired using LC–MS (Thermo Scientific Inc., Waltham, Massachusetts).

The analytical purity of target compounds was determined by reversed-phase HPLC in conjunction with product analysis by ESI–MS. UV absorption was detected from 200 to 800 nm using a diode array detector. The microanalytical technique was used to assess the integrity of all the produced substances. ^1^*H*NMR, ^13^*C*NMR and MS spectra (the details are explained in the experimental section and associated Additional file 1).

**6-iodo-1,2,3,4-tetrahydroquinoline (1a)** 
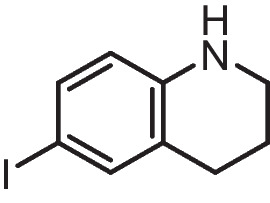


NaHCO_3_ (1.11 mmol, 3.0 equiv) in water (10 mL) and iodine (0.55 mmol, 1.5 equiv) were added to a solution of 1,2,3,4-tetrahydroquinoline **I** (0.37 mmol, 1.0 equiv) in tetrahydrofuran (6.5 mL) at 0 °C. The reaction mixture was agitated at an ambient temperature for one night. Subsequently, it was extracted using dichloromethane in three separate aliquots of 30 mL each. The organic layer, a combination of several substances, was washed using sodium thiosulfate. It was then dehydrated using anhydrous Na2SO4, filtered, and condensed. The unrefined residue underwent purification using column chromatography using hexane as the eluent, resulting in the production of iodide THQ **1a** as a yellowish oil (Yield 60%) comparable to those reported in the literature.

**6-iodo Chromane (1b)** 
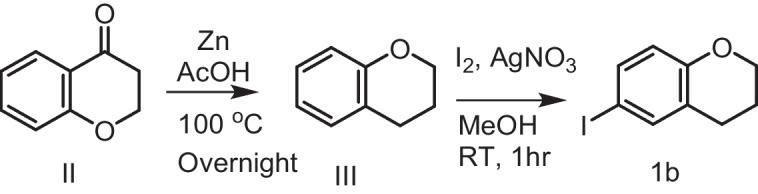


The compound chromanone **II** (0.978 g, 6.60 mmol) was dissolved in acetic acid (5 mL) and added to a suspension of zinc dust (10.8 g, 1.65 mol) in acetic acid (50 mL). The mixture was subjected to heating at a temperature of 100 °C and agitated for a night. It then passed through Celite®, washed with 100 mL of ethyl acetate. The resulting solution was diluted with 300 mL of phenylmethane and subsequently concentrated, resulting in the formation of intermediate chromane **III**.

Sequentially, 1.2 g (equivalent to 7.06 mmol) of AgNO_3_ and 1.58 g (equivalent to 6.23 mmol) of I_2_ were added to a mixture containing 0.845 g (equivalent to 6.3 mmol) of chromane **III** and 20 ml of MeOH. The mixture was stirred for approximately 1 h and then passed through a filter containing Celite®. The resulting liquid was then concentrated by reducing the pressure. The remaining substance was mixed with ethyl acetate (25 mL) and then rinsed with a solution of saturated sodium thiosulfate (25 mL). The aqueous layer was rinsed with 20 mL of water, dehydrated using Na_2_SO_4_, filtered, and then concentrated. The remaining substance was additionally refined using flash column chromatography using silica gel and a mixture of 5% ethyl acetate in hexanes, resulting in the formation of 6-iodochroman 1b (Yield 73% over 2 steps). All spectroscopic and analytical properties were identical to those reported in the literature [29].

**Methyl 6-(4,4,5,5-tetramethyl-1,3,2-dioxaborolan-2-yl)−2-naphthoate 2** 
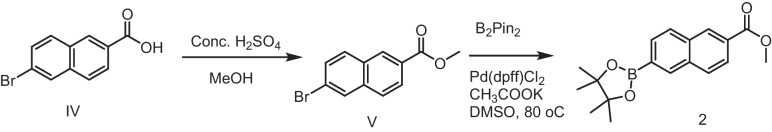


6-Bromo-2-naphthalenecarboxylic acid (1.2 g, 5 mmol) was dissolved in 20 mL of anhydrous methanol. Subsequently, a volume of 1 mL of concentrated sulfuric acid was carefully introduced dropwise, and the system was subjected to reflux for the duration of the entire night. The progress of TLC was monitored until the raw material conversion was finished, and then the heating ceased. Once the temperature had decreased to room temperature, a concentrated solution of sodium carbonate in water was introduced. The reaction system was modified to achieve a neutral state. It was then extracted using ethyl acetate. The resulting organic phase was subsequently washed three times with anhydrous sodium sulfate. **V** was obtained through concentration under lowered pressure (1.2 g, 96% yield) as a white solid. M.p. = 125–126 as reported [30].

Methyl 6-bromonaphthalene-2-carboxylate (1.00 g, 3.77 mmol), B2Pin2 (1.05 g, 4.15 mmol), KOAc (1.11 g, 11.32 mmol), and Pd(dppf)Cl_2_.CH_2_Cl_2_ (0.1 g, 0.113 mmol) were dissolved with anhydrous DMSO (10 mL, degassed using the freeze–pump–thaw method) in an atmosphere of argon. The resulting suspension was agitated at a temperature of 80 ◦C for the entire night. It was subsequently cooled, filtered through Celite® (which was washed with 100 mL of ethyl acetate), and then diluted with water. After that, it was extracted with ethyl acetate three times. The mixture of organic compounds was washed with water and brine, dehydrated using magnesium sulfate, and then concentrated to obtain an impure solid substance. The crude substance is subjected to flash column chromatography using silica gel and a mixture of 5% ethyl acetate in hexanes as the eluent. This process resulted in the isolation of methyl 6-boroate-2-naphthoate 2, with a yield of 1.00 g (87%), in the form of a white solid. All spectroscopic and analytical properties were identical to those reported in the literature [31].

**Methyl 6-substituted-2-naphthoate (3a-b)**

Compound 6-iodo-1,2,3,4-tetrahydroquinoline **1a** (0.180 g, 0.7 mmol), and 6-iodochromane **1b** (0.182 g, 0.7 mmol) were dissolved in DMSO/H2O (6 mL, 5:1), and the resultant solution was degassed via freeze–pump–thaw. Under Ar, compound **2a** (0.240 g, 0.77 mmol), K3PO4 (0.326 g, 1.54 mmol), and Pd(dppf) Cl_2_ (19 mg, 0.0231 mmol) were added, and the resultant suspension was stirred at 80 °C for 48 h. The solution was cooled, passed through celite to remove palladium, diluted with H_2_O, and extracted with EtOAc (3 ×). The organics were washed with H_2_O and brine, dried (MgSO_4_), and evaporated to give a crude solid. This was purified by silica gel chromatography (hexane/EtOAc) to give compound **3(a-b).**

**Methyl 6-(1,2,3,4-tetrahydroquinolin-6-yl)−2-naphthoate (3a)** 
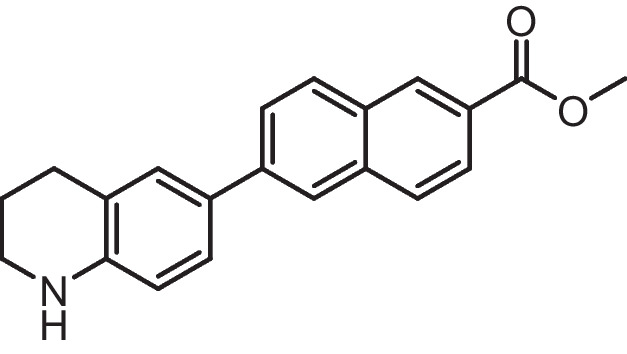


Orange solid (Yield = 72%), ^1^H NMR (400 MHz, DMSO) δ 8.58 (s, 1H), 8.12 (d, *J* = 5.5 Hz, 1H), 8.09 (s, 1H), 8.01 (d, *J* = 8.7 Hz, 1H), 7.94 (dd, *J* = 8.6, 1.6 Hz, 1H), 7.87 (dd, *J* = 8.7, 1.7 Hz, 1H), 7.41 (d, *J* = 7.7 Hz, 2H), 6.56 (d, *J* = 8.4 Hz, 1H), 6.00 (s, 1H, D2O exchangeable), 3.92 (s, 3H), 2.78 (t, *J* = 6.2 Hz, 2H), 2.29 (t, *J* = 7.4 Hz, 2H), 1.88–1.77 (m, 2H).

**Methyl 6-(chroman-6-yl)−2-naphthoate (3b)** 
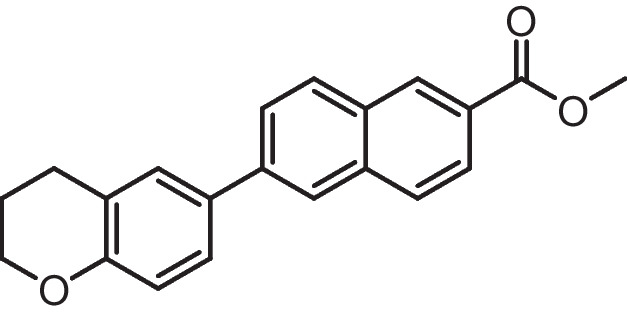


White solid (Yield = 74%), ^1^H NMR (400 MHz, DMSO) δ 8.63 (s, 1H), 8.23 (s, 1H), 8.18 (d, *J* = 8.7 Hz, 1H), 8.07 (d, *J* = 8.7 Hz, 1H), 7.99 (dd, *J* = 8.6, 1.6 Hz, 1H), 7.95–7.86 (m, 1H), 7.58 (d, *J* = 8.0 Hz, 2H), 6.88 (d, *J* = 8.2 Hz, 1H), 3.93 (s, 3H), 2.86 (t, *J* = 6.4 Hz, 2H), 2.29 (m, 2H), 1.98 (t, 2H).

**6-(substituted)−2-naphthoic acid (4a-b)**

A solution of Methyl 6-substituted-2-naphthoate **3a-b** (0.35 g, 1 mmol) in THF (20 ml) was treated with aqueous 20% NaOH (20 ml). After heating at 70 ***◦***C for 20 h, the reaction mixture was diluted with ethylacetate (10 ml) and water (10 ml), and then 1 M HCl solution was added until the mixture reached pH 1. The organic layer was separated, washed with ethylacetate three times, then dried with (MgSO_4_) and evaporated to give the afforded compounds. This was purified by silica gel chromatography (hexane/EtOAc/0.1 acetic acid) to give compound **4(a-b).**

**6-(1,2,3,4-tetrahydroquinolin-6-yl)−2-naphthoic acid (4a)** 
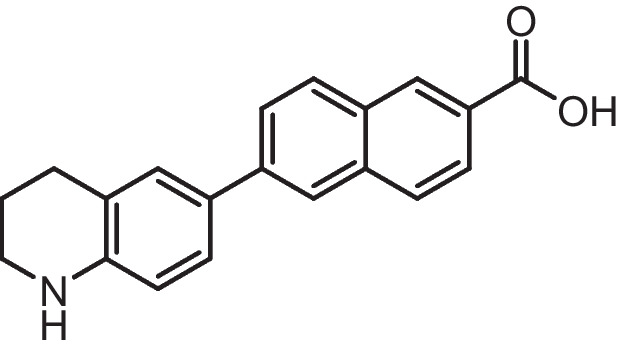


Brown solid (Yield = 69%), ^1^H NMR (400 MHz, DMSO) δ 8.51 (s, 1H), 8.18 (d, *J* = 5.5 Hz, 1H), 8.09 (s, 1H), 8.01 (d, *J* = 8.7 Hz, 1H), 7.94 (dd, *J* = 8.6, 1.6 Hz, 1H), 7.87 (dd, *J* = 8.7, 1.7 Hz, 1H), 7.41 (d, *J* = 7.7 Hz, 2H), 6.56 (d, *J* = 8.4 Hz, 1H), 6.00 (s, 1H, D_2_O exchangeable), 2.78 (t, *J* = 6.2 Hz, 2H), 2.29 (t, *J* = 7.4 Hz, 2H), 1.88–1.77 (m, 2H). ^13^C NMR (101 MHz, DMSO) δ 168.18, 146.02, 140.90, 136.11, 135.32, 132.53, 130.84, 130.60, 130.09, 129.64, 128.83, 128.64, 128.51, 128.09, 128.03, 127.35, 126.19, 125.87, 125.79, 122.65, 121.04, 114.26, 41.15, 27.24, 25.16. ESI–MS (M + Na) + calcd for C_20_H_17_NO_2_ 325.1259, found 325.2571.

**6-(chroman-6-yl)−2-naphthoic acid (4b)** 
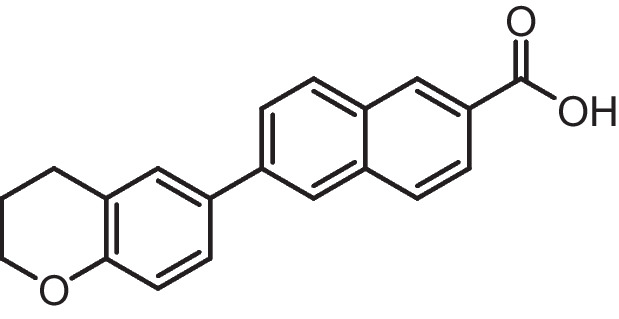


White solid (Yield = 72%), ^1^H NMR (400 MHz, DMSO) δ 13.05 (s, 1H, D2O exchangeable), 8.59 (s, 1H), 8.19 (s, 1H), 8.13 (d, *J* = 8.7 Hz, 1H), 8.03 (d, *J* = 8.6 Hz, 1H), 7.98 (d, *J* = 8.5 Hz, 1H), 7.87 (d, *J* = 8.5 Hz, 1H), 7.56 (s, 1H), 6.86 (d, *J* = 8.2 Hz, 1H), 4.17(t, *J* = 6.2 Hz, 2H), 2.84 (t, *J* = 6.2 Hz, 2H), 1.96 (dd, *J* = 10.5, 5.4 Hz, 2H). ^13^C NMR (101 MHz, DMSO) δ 167.91, 155.26, 140.11, 135.91, 131.60, 131.38, 130.71, 130.26, 129.01, 128.75,128.11, 126.30, 126.14, 125.98, 124,34, 123.38, 117.40, 66.58, 29.49, 24.86. ESI–MS (M + H) + calcd for C_20_H_16_O_3_ 305.1099, found 305.1356.

### In-vitro Evaluations

#### Retinoids and Chemical Reagents

DMSO (Sigma-Aldrich, St. Louis, MO, USA) was used to produce stock solutions of the synthetic and natural retinoids, ATRA, to a final concentration of 10 mM, and divided into aliquots, then stored at −20 °C. During storage and experimentation, the ATRA stock solution and the aliquots of working concentrations were kept out of the direct light of the laboratory. Piochem provided malachite green, ammonium molybdate, polyvinyl alcohol, and ATP. The materials were maintained in accordance with the manufacturer's instructions.

#### Cell Culture

All cancer cell lines, as well as normal fibroblast cells (WI-38), were purchased from the cell culture bank in the tissue culture unit at the appropriate institute. The cell lines were tested for mycoplasma contamination and maintained in the research center, according to the standard protocol [20, 22, 32–34].

#### Assay for Anti-Proliferative Activity

The researchers tested all synthetic retinoids (**3a**, **3b**, **4a**, and **4b**) in addition to ATRA as a standard retinoid positive control. The antiproliferation and cytotoxicity were assessed using 3-(4,5-dimethylthiazol-2-yl)−2,5-diphenyltetrazolium bromide (MTT, Serva) colorimetric assay [35]. All tested cancer cell lines were seeded at a density of 20,000 cells per well in 96-well plates and allowed to adhere overnight. The attached cells were treated with five serial dilutions (100, 50, 5, 0.5, and 0.1 μM) in triplicate. These concentrations were prepared by diluting the stock solutions with serum-free culture media. The negative control cells were those treated with 0.1% DMSO solvent alone. The treated cells were handled in reduced light and maintained under standard culture growth conditions for 24 h. After the incubation period, MTT powder was prepared as a stock solution (5 mg/mL), and 20 μL of the MTT working reagent (final concentration 0.5 mg/mL) was added to each well. This was followed by incubation with MTT reagent for 4 h in a humidified atmosphere (37 °C, 5% CO_2_) with subsequent addition of 150 μL of DMSO solubilizing solvent and incubation for 20 min. The absorbance of solubilized violet formazan crystals was measured at 570 nm with a Biotek 800 TS microplate reader. IC_50_ was calculated as the concentration of retinoid that produced 50% cell growth inhibition. For cytotoxicity assessment, WI-38 normal human fibroblast cells were used for the calculation of SI, which is the ratio of IC_50_ retinoid (WI-38)/IC_50_ retinoid (cancer cell line) [36]. Increasing the SI value above 1 indicates a more effective and safer drug as an anticancer compared to normal tissues [37].

#### Apoptosis Assay Using Annexin V (AV)/Propidium Iodide (PI)

The standard protocol used in HSBR was performed [20, 22]. Briefly, Caco-2 cancer cells were seeded into 6-well plates at a density of 1 × 10^6^ cells and left overnight to attach, followed by treatment for another 24 h with the IC_50_ doses of ATRA or synthetic retinoid analogues (**3a**, **3b**, **4a**, and **4b**). Cells were rinsed with 1 mL of binding buffer; the cells were again suspended in phosphate-buffered saline (PBS) (Lonza). 100 µL of cell suspension was incubated with 1 µL of FITC-labeled Annexin-V and 5 µL of PI for 15 min at 4 °C in the dark. The suspension was then mixed with 400 µL of ice-cold binding buffer, and each sample's apoptotic cells were analyzed using a Cytoflex flow cytometer (Beckman Coulter, USA), and CytExpert software (version 2.4.0.28) was employed for data analysis.

#### Cell Cycle Assay Using Propidium Iodide (PI) Staining

The standard protocol used in HSBR was performed [20, 22]. Briefly, 1 × 10^6^ Caco-2 cancer cell lines were seeded into 6-well plates overnight to attach, followed by treatment for another 24 h with the IC_50_ doses of ATRA as a positive control or synthetic retinoid-analogues (**3a**, **3b**, **4a**, and **4b**). Cells were concentrated at 1500 xg and suspended in 50 μg/ml propidium iodide (PI) staining solution and 20 g/ml RNaseA to identify cells with a sub-G1 DNA content. Cytoflex flow cytometer (Beckman Coulter, USA) was used, and CytExpert software (version 2.4.0.28) was employed for data analysis. The gating strategy used to eliminate doublets depends on plotting the population against a 2D contour plot in four quadrants, referred to as Q1(LL), Q2 (LR), Q3 (UL), and Q4 (UR), that illustrates the spread of the Annexin-FITC-H core cell population against PI PE-H.

#### Gene Expression Analysis Using RT-qPCR

Apoptosis-linked genes (Caspase-3 and Cytochrome-C), retinoic acid receptors (RAR-α, RAR-β, RAR-γ), and retinoic acid binding proteins (CRABP I and II) were analyzed after applying a treatment of the Caco-2 cancer cell line with ATRA and new synthetic retinoids for 24 h using their IC_50_ dose. The gene expression analysis was performed using real-time quantitative PCR (RT-qPCR), as previously documented [38]. After incubation, cells were harvested, and total RNA was extracted using the Favor-PrepTM Blood/Cultured Cell Total RNA Purification Mini kit (Favorgen Biotech Corp., Ping-Tung, Taiwan). The purified RNA was then reverse transcribed into the first-strand cDNA using the Revert Aid First-Strand cDNA Synthesis Kit (Thermo Scientific, Waltham, MA, USA). All qPCR reactions were carried out using the HERAPLUS SYBR® Green qPCR Kit (Willowfort, Nottingham, UK). All primer sequences with corresponding accession numbers/weblinks, including any reference, are listed in Table 1. Differential gene expression was performed using the 2ˆ^−ΔΔCT^ method utilizing GAPDH as a reference gene [39].

**Table 1:** Primer’s sequence of key modulatory genes used for gene expression analysis

Gene	Forward primer sequence	reverse primer sequence	GenBank accession number	References
Caspase-3	5'- ACATGGAAGCGAATCAATGGACTC −3'	5'- AAGGACTCAAATTCTGTTGCCACC −3'	NM_001354783.2 NM_032991.3 NM_001354779.2 NM_001354777.2 NM_001354780.2 NM_001354784.2 NM_001354781.2 NM_001354782.2 NM_004346.4	[40, 41]
Cyt-c	5'- GAGGCAAGCATAAGACTGGA −3'	5'- TACTCCATCAGGGTATCCTC −3'	NM_018947.6	[42]
CRABP 1	5'- AGGTCGGAGAAGGCTTTGAGGA −3'	5'- TCACGGGTCCAGTAGGTTTTGG −3'	NM_004378.3	https://www.origene.com/catalog/gene-expression/qpcr-primer-pairs/hp207699/crabp1-human-qpcr-primer-pair-nm_004378
CRABP 2	5'- GGGTGGAGTTTGACGAACACACAA −3'	5'- TTTGAACACTTGTCGGCACACCTG −3'	NM_004164.3	[43]
RAR-α	5'- GGGCAAATACACTACGAACAACA −3'	5'- CTCCACAGTCTTAATGATGCACT −3'	NM_001024809.4 NM_001145302.3 NM_001145301.3 NM_000964.4	[20, 44–46]
RAR-β	5'- TCGGCACACTGCTCAATC −3'	5'- GAAGCAGGGTTTGTACACTCG −3'	NM_000965.5 NM_001290277.1	[20, 47, 48]
RAR-γ	5'- CTGGAGATGGATGACACC −3'	5'- GTTCTCCAGCATCTCTCG −3'	NM_001243730.2 NM_001042728.3 NM_001243731.2 NM_001243732.2 NM_000966.6 NM_011244	[20, 49, 50]
GAPDH	5'- CTGACTTCAACAGCGACACC −3'	5'- TAGCCAAATTCGTTGTCATACC −3'	NM_001357943.2 NM_001256799.3 NM_001289745.3 NM_001289746.2 NM_002046.7	[20, 51, 52]

#### Western Blotting Analysis

Using the previously outlined protocol, the western blot technique was used to determine the expression of RAR-α (Cat. No. E6Z6 K, cell signaling, USA) [53], RAR-β (Cat. No. PP-H4338-00, R&D systems, USA) [54] and RAR- γ (Cat. No. D3 A4, cell signaling, USA) [55] in Caco-2 cells. Briefly, cells in triplets were seeded with 1 × 10^6^ per well in 6-well plates, and they were subsequently treated for 24 h with the IC_50_ dosage of ATRA as a positive control and synthetic retinoids (**3a**, **3b**, **4a**, and **4b**). Following incubation, floating adhered cells were separated and lysed. The Caco-2 cell lysate was immediately collected, subjected to sonication, and centrifuged for 15 min at 12,000 × g. The total soluble protein content was measured colorimetrically using the PierceTM 660 nm Assay (Thermo Scientific, Waltham, MA, USA) after the resultant supernatant was separated. The Gel documentation system (Geldoc-it, UVP, England) was applied for data analysis using Totallab analysis software (Ver.1.0.1) (ww.totallab.com).

#### Assessment of ATPase Activity

The total activity of ATPase and calcium-independent ATPase activity was quantified colorimetrically as described by our previous protocol [22]. Briefly, Caco-2 cells were exposed to individual IC_50_ doses of ATRA as a positive control or synthetic retinoids (**3a**, **3b**, **4a,** and **4b**) for 24 h. The inorganic phosphate (Pi) liberated after the hydrolysis of ATP by ATPase enzymes was complexed into a colored product in one step after the addition of a mixed reagent containing malachite green, ammonium molybdate, and polyvinyl alcohol (2:1:1). The intensity of absorbance at 630 nm was measured using the BioTek 800 TS absorbance plate reader (USA) and was directly proportional to the quantity of liberated Pi. In order to eliminate any color due to the non-enzymatic hydrolysis of ATP, control reactions were made using only ATP in the reaction buffer, and the absorbance intensity was then subtracted from the readings of the samples and standards [56].

#### IL-6 and IL-10 Inhibitory Activity

For IL-6 and IL-10 estimations, Caco-2 cells were subjected to the IC_50_ of the ATRA as a positive control or synthetic retinoids (**3a**, **3b**, **4a,** and **4b**) for 24 h. For IL-10. Moreover, IL-6 measurements and cell pellets were lysed using RIPA buffer, and the total lysate was collected. The total protein was estimated to use commercially available ELISA kits as per the manufacturer’s instructions. ELISA plates were coated and used for analysis according to the previously used protocol using capture antibody (IL-6, Cat. No.: RAB0307 [57] and IL-10, Cat. No.: RAB1060 [58]), followed by blocking using 5% BSA in PBS-Tween buffer for 1 h. After incubating with samples for 2 h, a secondary HRP-detection antibody was added and kept for incubation for 2 h. Streptavidin-HRP reagent was then added to the plates, followed by the addition of TMB solution. Finally, the reaction was stopped using 2 N H_2_SO_4_, and the color was measured using the BioTek 800 TS absorbance plate reader (USA) at 450 nm wavelengths, and the protein was quantified.

#### Fluorescent Cellular Characterization

Carl Zeiss LSM 710 confocal microscope laser scanning confocal microscope with a sensitive detector used to capture cellular fluorescent changes. Using the IC_50_ dose of ATRA as a positive control or synthetic retinoids (**3a**, **3b**, **4a**, and **4b**), cells were treated and followed by fixation with paraformaldehyde/PBS (PFA, 4%). Using 405 nm, 488 nm, 594 nm, or 633 nm lasers, samples were imaged and scanned for full spectra using Plan-Apochromat Zeiss 63 × oil phase and suitable working distance objective lens according to previously used protocols [59, 60].

#### Fluorescent Cellular Co-Localization

Co-staining of cells attached to the coverslip inside chambered plates was implemented utilizing IC_50_ doses of ATRA as a positive control or new synthetic retinoids (**3a**, **3b**, **4a,** and **4b**) for 24 h, followed by using 10 μg mL^−1^ of Nile Red (Fisher Scientific, Cat. No. 11549116) in a way similar to the previously used hypothesis [61, 62]. Cells were washed twice with PBS (pH 7.2) and visualized using the Axio Observer 7 fluorescent microscope. Co-localization analysis was performed using ZEN software after the background was removed.

### In-silico Studies

#### Prediction of Bioactivity Profile of New Retinoids Studied

SwissADME® (https://swissadme.ch) was the online tool used to perform the computational prediction for new synthetic retinoids. This tool allowed for the retrieval of relative results pertaining to physicochemical parameters (lipophilicity (logP), molecular weight, polar surface area, number of hydrogen bond donors and acceptors, number of rotary bonds, and solubility in water), drug-likeness profile, and pharmacokinetic profile (absorption, distribution, metabolism, excretion, and toxicity) of the molecules and the bioactivity profile (pharmacodynamics).

#### Toxicological Prediction of the New Synthetic Retinoids

Using the ProTox-II webserver (https://tox.charite.de/protox3/), created by Drwal et al. [63], an *in-silico* study was conducted using parameters such as rat oral acute toxicity, with particular reference to the median lethal dose (LD_50_) as mg/kg, organ toxicity (particularly hepatotoxicity), immunotoxicity, genetic toxicity endpoints, and cytotoxicity.

#### Similarity Search and Target Prediction

PubChem (https://pubchem.ncbi.nlm.nih.gov/) and ChEMBL (https://www.ebi.ac.uk/chembl/) interfaces were used to perform the similarity search [64]. Each server's built-in sketcher feature was used to create sketches of the novel synthesized retinoid compounds. After assessing each of the top-ranked hits for biological activity, comparable compounds with cytotoxic action were ruled out [65].

#### Molecular Docking Simulation

The potential binding mode of ATRA and other new synthetic retinoids was explored using the active sites of RAR-α (PDB = 3 KMR), RAR-β (PDB = 1XAP), and RAR-γ (PDB = 2LBD) that were retrieved from the protein data bank (https://www.rcsb.org/). All these proteins were downloaded, and original crystallized bound ligands were extracted and utilized as a positional reference for validation of the docking protocol. ChemBioDraw 2014 was used to create 3D structures. In addition, ATRA and acid forms were protonated, as previously mentioned [19]. The docking study employed Autodock Vina [66] and OpenBabel [67] tools, while the results visualization employed Discovery Studio [68]. Autodock Vina employs a united-atom scoring function and an amber force field. Furthermore, Vina employs a gradient-based local search genetic algorithm, a global optimization algorithm, to forecast the binding mode of small molecules to their target. The ligands and proteins were initially prepared and stored in the pdbqt format using OpenBabel tools. Subsequently, the active sites of RAR-α, RAR-β, and RAR-γ were identified by the binding of the co-crystallized ligand (AM580, TTNPB, and ATRA) with the dimensions of 20*20*20 Å in the x, y, and z orientations. The validation procedure, as well as redocking, was performed using the original co-crystallized ligands in each RAR protein, and binding energy (S), RMSD scoring function, and interacting residues were retrieved for comparison with ATRA and new synthetic retinoid docked structures.

#### Molecular Dynamic Simulation

The complexes with the most favourable docking scores (**4a**-RAR-α, **3b**-RAR-β, and **3b**-RAR-γ) were further evaluated through molecular dynamics (MD) simulations using previously used protocols [69, 70] to assess the stability of their interactions under more realistic, dynamic conditions. The topology parameters of the ligands were generated using the ACPYPE server (https://www.bio2byte.be/acpype/) with the General Amber Force Field (GAFF). For the proteins’ topology, GROMACS 2024.2 was employed, utilizing the AMBER99SB force field [71]. Each protein–ligand complex was solvated in a cubic box with a simple point charge (SPC) water model and neutralized by adding Na^+^ counter ions. The neutralized systems underwent energy minimization using the steepest descent algorithm in 50,000 steps or Fmax < 100 kJ/mol. Subsequently, the minimized systems were equilibrated in two steps: first, the Number of particles, Volume, and Temperature (NVT) ensemble, where the temperature was controlled at 300 K for 200 ps using the V-rescale thermostat, followed by the Number of particles, Pressure, and Temperature (NPT) ensemble, where the pressure was controlled by the Parrinello-Rahman barostat algorithm for another 200 ps. Finally, the MD simulations were performed for 100 ns under an NPT ensemble with a time step of 2 fs. Long-range electrostatic interactions were calculated using the Particle Mesh Ewald (PME) algorithm. Hydrogen bond lengths were constrained employing the Linear Constraint Solver (LINCS) algorithm.

Following the MD simulations, periodic boundary conditions were removed, and the trajectories were analyzed using GROMACS tools like *rms* for Root Mean Square Deviation (RMSD), *rmsf* for Root Mean Square Fluctuation (RMSF), *gyrate* for Radius of Gyration (RG), *sasa* for Solvent Accessible Surface Area (SASA) and *hbond* to calculate the number of hydrogen bonds.

### Statistical Analysis

Statistical analyses were performed using GraphPad Prism, version 7 (GraphPad Software, Inc.). Data are presented as one-sided tests from triplicates as the mean ± SD. ANOVA test was used with a post hoc test. *P* < *0.05* was considered to indicate a statistically significant result.

## Results and Discussion

### Concept of Designing Synthetic Retinoid Fluorescent Probes

The majority of fluorescent probes used in cellular imaging are typically created by attaching a biocompatible fluorophore via an appropriate linker to the target protein ligand's bioactivity-tolerable region [72, 73]. Nevertheless, the resultant fluorescent probes frequently have large molecular sizes, which impairs their ability to bind molecular targets with a weak affinity and low cell membrane permeability [74]. Therefore, it is desirable to have architecturally fluorescent probes without an extra connected fluorophore. Since most synthetic retinoids have highly conjugated structures by nature, it is acceptable to alter these scaffolds to create stable, naturally highly fluorescent retinoids that might provide information about their immediate environment. This concept's realization is demonstrated by replacing the electron-rich dihydroquinoline in some active retinoid’s hydrophobic region of pan-RAR agonists, such as EC23, by lone pair electron-donating atoms such as oxygen (chroman) or nitrogen (tetrahydroquinoline) to propagate and enhance conjugation. This enhances the diphenylacetylene structure's donor–acceptor character and results in a notable bathochromic shift in λmax within the retinoid structure to acquire intrinsic fluorescent properties.

### Chemistry

**Fig. 3:**
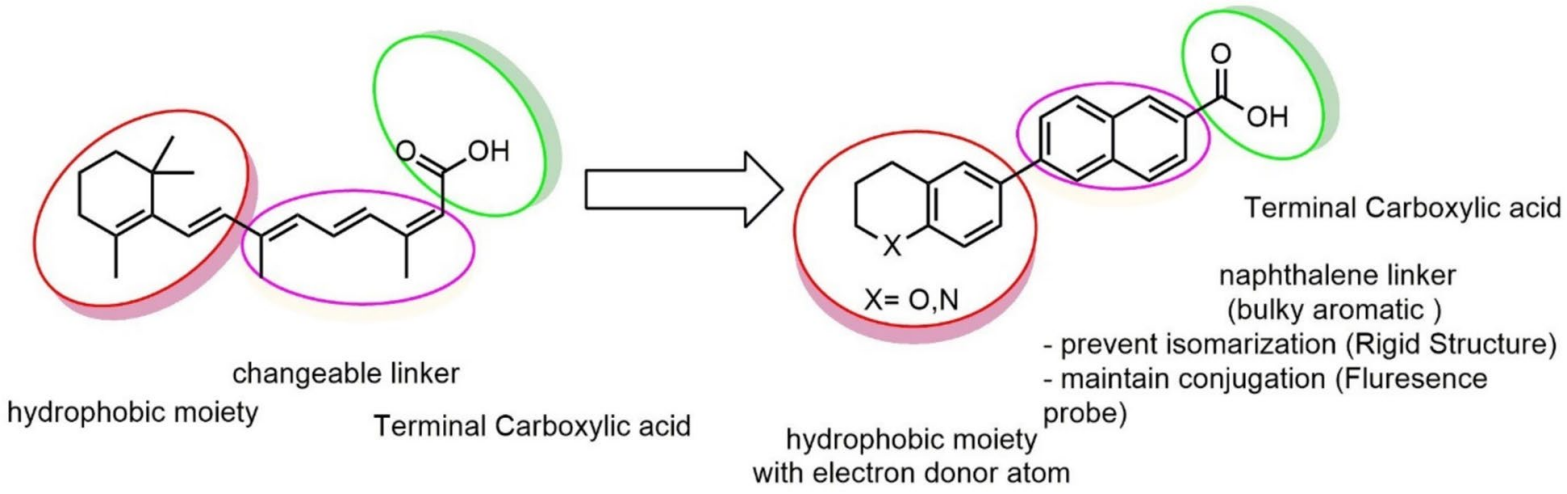
Rational design of the novel fluorescent synthetic retinoids

ATRA is susceptible to isomerization and oxidation, which can result in substantial alterations to the compound's activity and selectivity [75]. These modifications may be particularly intriguing when examining its poisonous impact on non-tumor cells. Enhancing the chemical stability of synthetic analogues could be a solution to address these issues and potentially make them valuable pharmacological agents with reduced toxicity. The substantial chemical alterations to the primary key pharmacophore structure of retinoids include a hydrophobic moiety, a changeable linker, and an acidic function. Substituting the polyene chain with an aromatic ring can help to augment the chemical and physical durability of the synthetic retinoid in comparison to the parent retinoic acids, i.e., unstable heat and light under ambient environmental conditions. Consequently, it was suggested that the inclusion of a naphthalene moiety would not only offer an appropriate linear arrangement but would also offer a non-isomerizable linker unit and enhance the internal resonance structure and fluorescence property. Thus, it was suggested to design and synthesize isomeric compounds with terminal carboxylic or ester groups that could imitate various natural retinoic acid isomers by altering the position of trimethyl cyclohexene with either tetrahydroquinoline or chromane (Fig. 3).

**Scheme 1:**
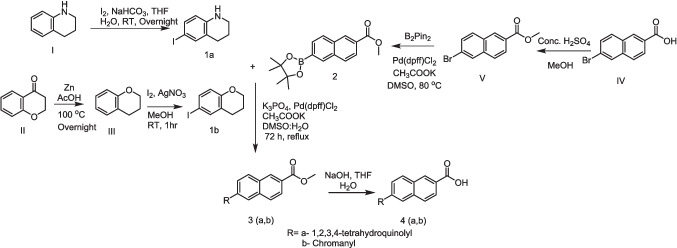
Synthesis of naphthoaic acid Derivatives

Iodide derivatives **1a** and **1b** were efficiently prepared using an effective method [76], resulting in yields of 60% and 73%, respectively (Scheme 1). The Miyaura borylation procedure facilitates the production of boronate derivatives **2** through the cross-coupling of bis(pinacolato)diboron (B_2_pin_2_) with methyl 6-bromo-2-naphthoate [77]. The selection of KOAc as a base is essential for the successful execution of the borylation reaction. The synthesis of isomeric retinoid esters **3a-b** was achieved by coupling highly lipophilic, electron-rich iodo tetrahydroquinolines (THQ) and iodo chromane using the Suzuki–Miyaura methodology. The reaction took place at 80 °C temperature with a 3% Pd catalyst, as iodide reacts faster with conjugated boronate acceptors (2). This resulted in an 88% yield of the desired esters. The esters were hydrolyzed using an aqueous solution of sodium hydroxide in tetrahydrofuran (THF) to obtain the required retinoid structures **(4a-b).** (Scheme 1).

The ^1^H-NMR spectra of compounds **3a-b** exhibited a single signal at 3.92 and 3.93 ppm, which corresponds to the methyl ester group. Furthermore, the aliphatic proton peaks for THQ and chromanyl, such as the methylene group near the aromatic ring, exhibited triplet peaks at 2.29 and 2.84 ppm, respectively. Additionally, the methylene group near the heteroatoms nitrogen and oxygen displayed triplet peaks at 2.78 and 4.17 ppm, respectively. Moreover, a distinct singlet peak at 6.00 ppm, which is exchangeable with deuterium oxide (D_2_O), corresponded to the NH protons of compound **3a**. Compound **4b** exhibited a notable exchangeable broad singlet peak at 13.05 ppm in D_2_O, which corresponds to the presence of acidic OH protons.

The ^13^C-NMR spectra of compounds **4a-b** exhibited peaks at 168.18 and 167.91 ppm, respectively, which corresponded to the carbon atoms in the carbonyl group of carboxylic acid. Furthermore, the peaks corresponding to the aliphatic ring of THQ and the chromanyl group were observed within the anticipated range for compounds containing these functional groups. Compound **4a** exhibited three peaks in the aliphatic area at 41.15, 27.24, and 25.16 ppm, which corresponded to the THQ group. In contrast, compound **4b** presented three peaks in the aliphatic region at 66.58, 29.49, and 24.86 ppm, which corresponded to the chromanyl group.

### Biological Evaluations

#### Anti-Proliferative Evaluation

All the newly synthesized synthetic retinoid analogues were biologically screened in this study using ATRA as the standard positive reference of the retinoids'comparable mode of action. All compounds were examined for their *in-vitro* anti-proliferative activity against a panel of cancer cell lines, including Caco-2, MDA-MB231, HCT-116, HepG-2, MCF-7, and PC-3 in comparison to the WI-38 normal cell line. The evaluated anti-proliferative activity was measured using IC_50_ (in µM) utilizing a 4-parameter non-linear logistic model (Online resource 1) as well as a calculation of the safety index (Table 2). ATRA showed IC_50_ doses in cancer cells ranging from 2.21 µM (PC-3) to 38.77 µM (HCT-116), which are lower than its IC_50_ on WI-38, suggesting its anticancer activity [78]. Table 2 demonstrates that compounds (**3a**, **3b**, **4a**, and **4b**) show variable IC_50_ doses across different cancer cell lines, which illustrates that both natural and new synthetic retinoids retain differential inhibitory effects on tumor cell proliferation. Compounds **3a** and **3b** showed significant anti-proliferative potential with IC_50_ values mostly ≤ 50 µM compared to compounds **4a** and **4b**, suggesting the ester form with hydrophobicity has more substantially optimized cellular uptake than the polar acid form. Compound **3a** showed much lower IC_50_ across different cancer cell lines compared to ATRA and other synthetic retinoids with IC_50_ < 20 µM, followed by compound **3b**, which showed comparable effects except on HCT-116 (52.94 µM). Compound **4a** demonstrated a low IC_50_ dose on MDA-MB231 (2.42 µM) and PC-3 (2.78 µM). Similarly, compound **4b** showed a low IC_50_ dose on MDA-MB231 (2.28 µM), PC-3 (2.71 µM) in addition to Caco-2 (14.96 µM). Comparably, the cytotoxicity of compounds **3a** and **3b** was shown to be minimal with SI mostly > 1 compared to compounds **4a** and **4b,** indicating that these analogues may be more effective, safer, and more selective during any future i*n-vivo* treatment as anticancer agents in a given dose. Caco-2 cells showed previously known sensitivity as an *in-vitro* model for testing synthetic retinoid activity [20, 22]**.** Additionally, colorectal cancer is a resistant cancer cell type with limited treatment options [79]. Therefore, Caco-2 will be selected as a sensitive *in-vitro* model for further biological assessment of all new synthetic retinoids and to understand their potential mechanistic effect.

**Table 2:** Measured anti-proliferative activity for four new synthetic retinoid analogues (**3a**, **3b**, **4a**, and **4b**) compared to ATRA as the standard positive reference of the retinoids. Data presented as IC_50_ (µM) using different types of cancer cell lines panels as well as normal fibroblast cells (WI-38) with calculated Safety Index (SI) as IC_50_ of compound (WI-38)/IC_50_ of compound (cancer cell line). IC_50_ was presented as mean ± SEM as triplets

Measured IC_50_ (µM)											
Cell lines	ATRA			3a		3b		4a		4b	
	IC_50_	SI	IC_50_		SI	IC_50_	SI	IC_50_	SI	IC_50_	SI
WI-38	46.86 ± 2.8			39.23 ± 4.7		29.23 ± 2.1		43.73 ± 4.2		33.51 ± 3.4	
Caco-2	11.41 ± 0.3	4.11	9.58 ± 1.1		4.09	14.40 ± 0.36	2.03	47.27 ± 6.2	0.93	14.96 ± 0.4	2.24
MDA-MB231	10.37 ± 0.2	4.52	2.33 ± 0.6^**^		16.81	2.09 ± 0.1^**^	14.00	2.42 ± 1.3^**^	18.06	2.28 ± 0.2^**^	14.71
HCT-116	38.77 ± 0.9	1.21	10.16 ± 3.8^**^		3.86	52.94 ± 4.3	0.55	132.7 ± 6.8	0.33	36.87 ± 2.4	0.91
HepG-2	16.03 ± 1.3	2.92	6.37 ± 1.0^*^		6.16	12.00 ± 2.6	2.44	53.01 ± 4.9	0.82	59.52 ± 3.5	0.56
MCF-7	4.93 ± 0.4	9.51	13.82 ± 2.4		2.84	12.23 ± 3.2	2.39	64.81 ± 5.4	0.67	53.12 ± 5.4	0.63
PC-3	2.21 ± 0.1	21.17	18.90 ± 4.0		2.08	4.60 ± 1.7	6.36	2.78 ± 0.3	15.75	2.71 ± 0.2	12.35

#### Apoptosis Assay (Annexin-V Staining Assay)

Retinoids have been demonstrated to control cellular differentiation, proliferation, and apoptosis in a wide range of cell types [80]. Therefore, the newly synthetic retinoid analogues-induced cell death was assessed in Caco-2 cancer cells using the annexin-FITC/propidium iodide (PI) staining apoptosis assay. The apoptosis assay is used to identify the stage of apoptosis caused by drug treatment in four distinct 3D-plot quadrants: live cells (bottom left), early apoptotic cells (bottom right), late apoptotic cells (top left), and necrotic cells (top right) [81].

**Fig. 4:**
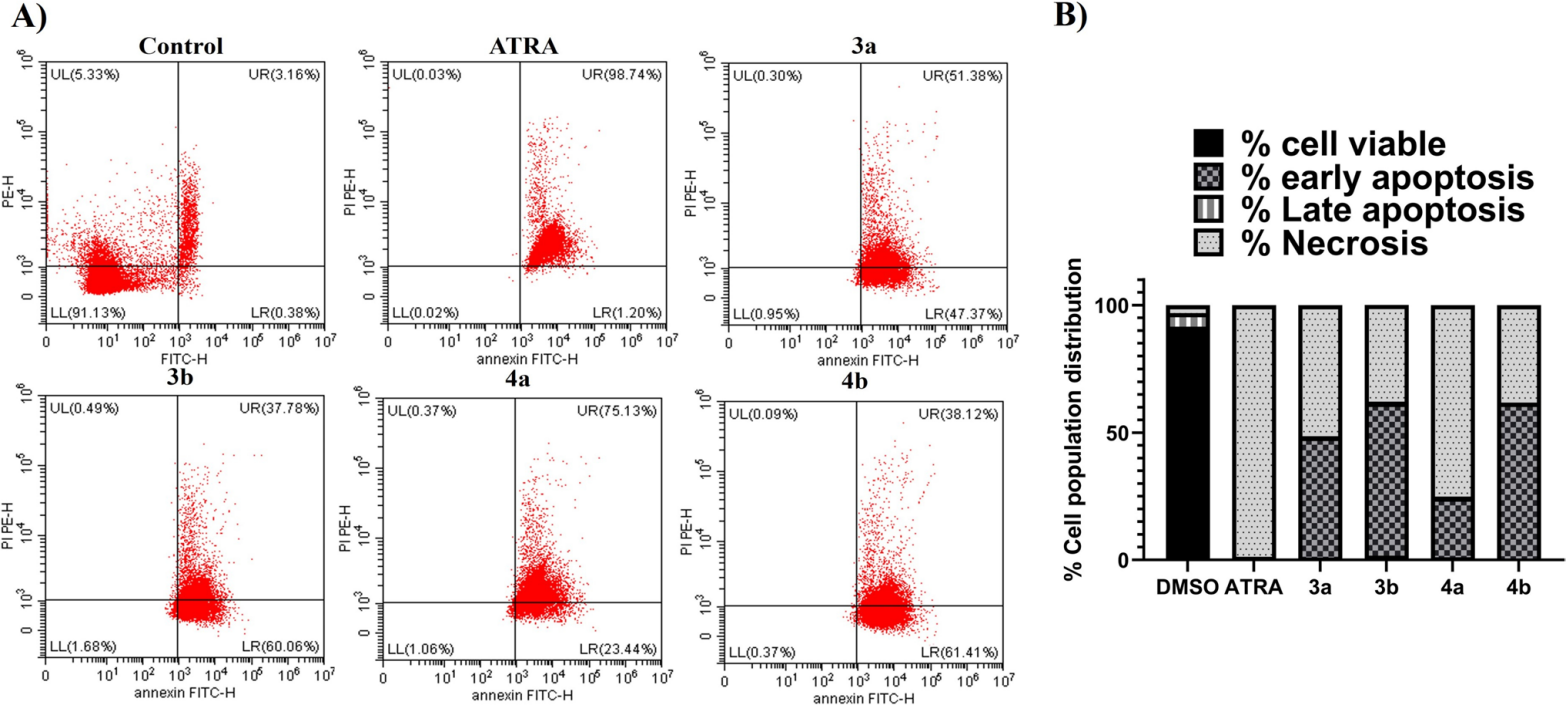
Annexin-V staining assay showing the apoptotic effect of ATRA and new synthetic retinoids (**3a**, **3b**, **4a**, and **4b**) on Caco-2 cells compared to the 0.1% DMSO negative control. **A) 2D** plots measuring the percentage of viable (LL), early apoptotic (LR), late apoptotic (UL), and necrotic cells (UR) by AV/PI assay using flow cytometry. **B)** Stacked bar columns of the apoptotic effect showing ATRA-induced cellular necrosis, while new synthetic retinoids induced mixed early apoptosis and necrosis with variable percentages (color not required in print)

Table 3 and Fig. 4 show the distribution of cells at each stage (quadrant) for determining the apoptotic potency of compounds with overlaid 2D plots. 0.1% DMSO negative control showed typical cell growth with 91% viable cells, which coincided with previously observed studies [82, 83]. ATRA as a positive control was able to induce a significant increase in necrosis with 98.74% compared to the negative control [22, 34, 84]. All new synthetic retinoids were able to induce early apoptosis, ranging from 23.44% by compound **4a** to 61.41% by compound **4b**. This was previously observed with several synthetic retinoids mediated through early DNA damage response and chromatin condensation, a mitochondrial process that is not dependent on caspase [85]. Additionally, the new synthetic retinoids were able to induce necrosis in a way similar to ATRA, ranging from 37.78% by compound **3b** to 75.13% by compound **4a**. Necrosis causes cells to lose their integrity as membranes break and internal components leak out, some of which act as warning signs to trigger inflammation. [86]. Hence, the presented data may suggest new synthetic retinoids induced cell death primarily through mixed early-stage apoptosis and necrosis. This needs to be confirmed by changes in multiple signaling pathways affecting apoptosis to confirm the apoptotic potency of the new synthetic retinoids on Caco-2 cells.

**Table 3:** Apoptosis assay measuring the percentage of viable, apoptotic, late apoptotic, and necrotic cells by AV/PI assay using flow cytometry. The assay was performed after the treatment of Caco-2 (colon cancer) for 24 h with ATRA (positive control), synthetic retinoids **3a**, **3b, 4a,** and **4b** compared to 0.1% DMSO negative control. Data are represented as mean ± standard error of the mean (SEM), n = 3

Tested compound	% Viable cells (LL)	% Early apoptotic cells (LR)	% Late apoptotic cells (UL)	% Necrotic cells (UR)
0.1% DMSO Control	91.13 ± 8.1	0.38	5.33 ± 0.1	3.16 ±
ATRA	0.02	1.20 ± 0.1	0.03	98.74 ± 8.7^***^
3a	0.95	47.37 ± 3.9^***^	0.30	51.38 ± 3.6^***^
3b	1.68 ± 0.1	60.06 ± 5.4^***^	0.49	37.78 ± 2.7^***^
4a	1.06 ± 0.1	23.44 ± 2.3^***^	0.37	75.13 ± 6.8^***^
4b	0.37	61.41 ± 5.3^***^	0.09	38.12 ± 2.8^***^

#### Cell Cycle Analysis

**Fig. 5:**
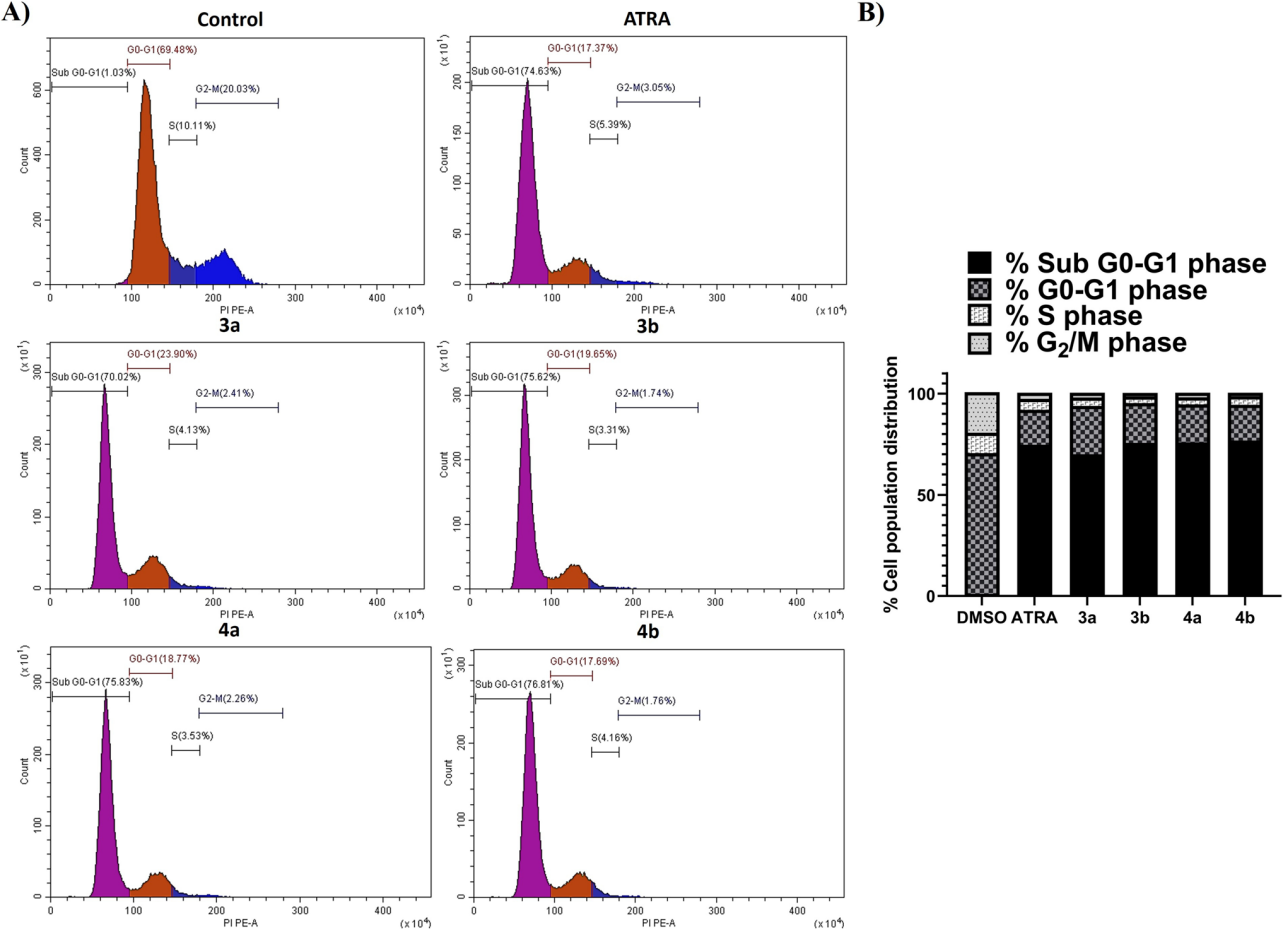
Cell cycle analysis using flow cytometry of treated Caco-2 cells for 24 h with ATRA (positive control) and new synthetic retinoids (**3a**, **3b**, **4a**, and **4b**) compared to 0.1% DMSO negative control; **A)** histogram analysis of cell population distribution for each treated compound showing cell cycle arrest mainly in the subG_0_-G_1_ phase and **B)** Stack bar showing the distribution of each cell population in percentage (color is not required in print)

Cell cycle analysis is another crucial tool with the apoptosis assay for evaluating the anticancer effect on the various cell cycle phases [87]. Cell cycle phases are crucial checkpoints for ongoing cellular proliferation [88]. Therefore, examining the effectiveness of particular new synthetic retinoid analogues in inducing cell cycle arrest in comparison to ATRA as a positive control is essential. Several studies have shown that new synthetic retinoids are able to induce cell cycle arrest through subG_0_-G_1_ phase arrest [20, 22, 89]. Hence, all new synthetic compounds were tested for their efficacy in cancer cell cycle arrest using their IC_50_ dose obtained from the MTT assay after 24 h. Table 4 and Fig. 5 indicate that the percentage of the arrested Caco-2 cells treated with **3a**, **3b**, **4a**, and **4b** was significantly accumulated in the SubG_0_-G_1_ phase (Fig. 5) than those in the control Caco-2 cells treated with 0.1% DMSO (**3a**; 70.02%, **3b**; 75.62%, **4a**; 75.83, and **4b**; 76.81% and control 0.1% DMSO; 1.03%, Table 4). The synthesized retinoids showed cell cycle arrest at the subG_0_-G_1_ phase similar to cells treated with ATRA and matched with earlier observations of our first generation of synthetic retinoids [20, 22]. This observation was accorded with the study of the adapalene's effect on the G_0_-G_1_ phase on colorectal carcinoma through inhibition of cyclin-dependent kinase 2 (CDK2), responsible for the cell cycle's G1-to-S phase transition, and cancer is characterized by its dysregulation [89]. This may suggest a similar mode of action for the new synthetic retinoids. In addition, all treated cells showed a significantly reduced percentage of G_0_-G_1_ phase compared to the negative control without effect on S-phase cells (Table 4), indicating that there was a comparable effect to ATRA on these phases of the cell life cycle. This observation was consistent with the data presented and might provide a possible explanation of cell cycle arrest observed at the G_0_-G_1_ phase in the cells treated with tested new synthetic retinoids [90, 91]. This may suggest that our new retinoids exert their anti-proliferative effects through induction of both apoptosis/necrosis and cell cycle arrest.

**Table 4:** Cell cycle analysis of Caco-2 (colon cancer) treated for 24 h with ATRA (positive control) and new synthetic retinoids (**3a**, **3b**, **4a**, and **4b**) compared to 0.1% DMSO negative control showing the DNA content at different cycle phases

Tested compound	% SubG_0_-G_1_	% G_0_-G_1_	% S	% G_2_M
0.1% DMSO Control	1.03 ± 0.1	69.48 ± 5.4	10.11 ± 1.7	20.03 ± 2.4
ATRA	74.63 ± 5.8^***^	17.37 ± 6.1	5.39 ± 1.4	3.05 ± 0.7
3a	70.02 ± 4.7^***^	23.90 ± 8.4	4.13 ± 1.2	2.41 ± 0.3
3b	75.62 ± 7.4^***^	19.65 ± 2.7	3.31 ± 1.1	1.74 ± 0.1
4a	75.83 ± 6.4^***^	18.77 ± 4.8	3.53 ± 1.3	2.26 ± 0.4
4b	76.81 ± 6.7^***^	17.69 ± 3.2	4.16 ± 1.5	1.76 ± 0.1

#### Quantitative Expression Analysis of Key Apoptosis-Related Genes

**Fig. 6:**
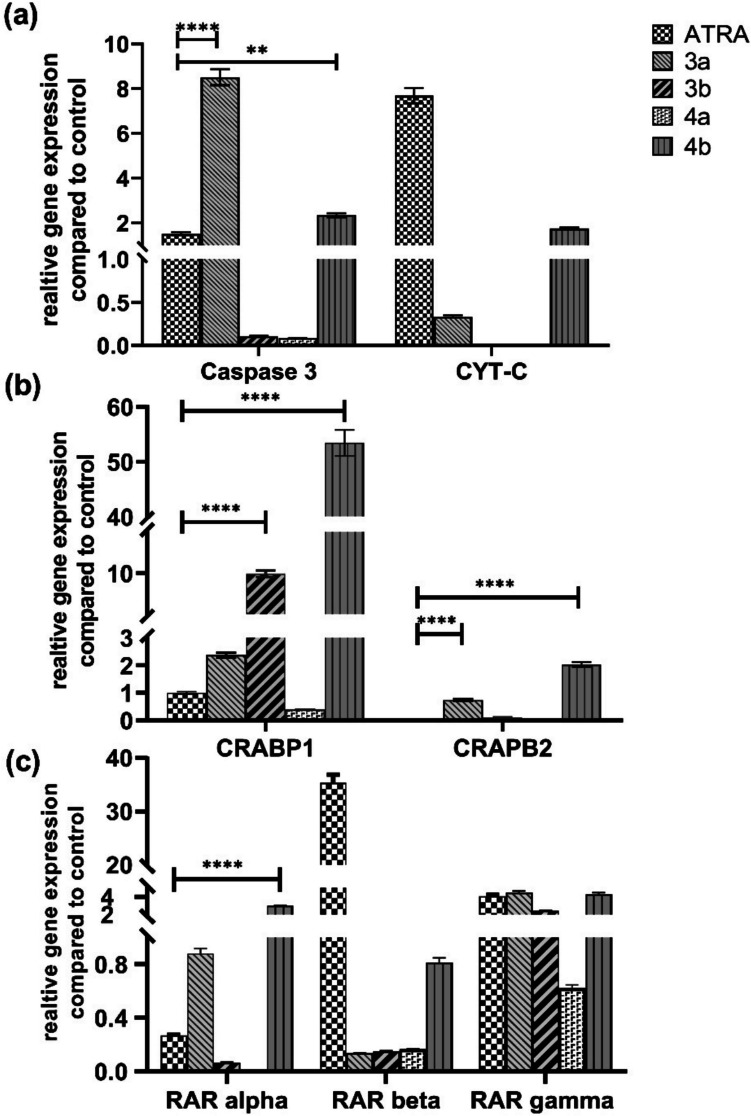
Analysis of different key genes expressions after treatment of Caco-2 cells with IC_50_ of new retinoids for 24 h. Shown are **A**) Key regulatory genes for extrinsic and intrinsic apoptosis, **B**) key cellular retinoic acid and, **C**) *RARs* (-*α*, -*β* and -*γ*). The quantification of target mRNA after retinoid treatment was relative to Caco-2 cells incubated with 0.1% DMSO vehicle for 24 h and was normalized to the internal reference gene glyceraldehyde 3-phosphate dehydrogenase (*GAPDH*). Relative gene expression was calculated by the 2^.^–ΔΔCt^ method and presented as an average of three independent experiments. The values are considered statistically significant compared to the solvent-treated control at ** *p* < 0.01, **** *p* < 0.0001. Data are represented as mean ± SEM, *n* = 3 (color is not required in print)

Apoptosis is an essential mechanism of action for retinoids as anticancer drugs via extrinsic or intrinsic mechanisms [92]. These two mechanisms always overlap to activate the caspase/protease family to eliminate cancer cells. Hence, it was essential to gain more insight into the activity mechanisms of new retinoids by assessing the alterations at the transcriptome level, especially apoptotic mediators, compared to ATRA as a positive control. *Caspase-3* and *Cytochrome-C* (*Cyt-C*) genes were screened as apoptotic genes in Caco-2 cells responsible for provoking apoptosis [93] and were evaluated on the mRNA levels as shown in Fig. 6.

Figure 6a demonstrates that ATRA induced a borderline expression of *caspase-3* with a 1.6 ± 0.1-fold change, while compounds **3a** and **4b** were able to significantly overexpress *caspase-3* compared to ATRA with 8.5 ± 0.4-fold and 2.3 ± 0.1-fold, respectively. Compounds **3b** and **4a** did not show any significant increase in the *caspase-3* level. On the other side, ATRA showed overexpression of the *Cyt-C* gene level with a 7.7 ± 0.3-fold change, and compound **4b** showed a 1.7 ± 0.1-fold change similar to ATRA, while compounds (**3a**, **3b**, **4a**) did not show any overexpression of the *Cyt-C* gene level. Cyt-C is released into the cytosol for caspase activation to occur since there is a significant correlation between the time courses of Cyt-C release from the mitochondria and caspase-3 activation [94]. This may explain the lower *caspase-3* expression level of compounds **3b** and **4a**. On the other side, some studies have reported an apoptosis-independent pathway of caspase activation regardless of Cyt-C release, and this may explain the behavior of compound **3b** for induction of apoptosis [95]. Another set of key genes are cellular retinoic acid binding proteins (*CRABPs*) that can bind with both natural and synthetic retinoids with different binding affinities [96]. CRABPs appear to perform unique variable roles, such as transporting retinoic acid to the nuclear receptors, guiding retinoic acid toward catabolism, and producing non-canonical activities without significant differences in roles between the two types [97]. All retinoids showed variable induction to any of CRABPs, and ATRA showed a 1.2-fold change in CRABPI with no effect on *CRABPII*. Besides, compounds **3a**, **3b**, and **4b** showed significant overexpression of *CRABPI* compared to *ATRA*: 2.4 ± 0.1, 9.9 ± 0.4, and 53.5 ± 2.4-fold changes, respectively (Fig. 6b). Moreover, compound **4b** was only able to induce *CRABPII* with a 2.0 ± 0.1-fold change expression. Additional activation of *CRABPII* is essential since it was demonstrated to aid in transporting retinoids from CRABPII to RAR in the nucleus and directly bind RAR, which is crucial since free retinoids are a strong uncoupler [98]. Finally, RARs mediate the growth inhibitory response in different cancer models relative to retinoid therapy since RARs are transcriptional transactivators, and their activation is an important step in figuring out the molecular mechanisms by which retinoids limit the growth of cancer [14, 99]. Retinoids bind to RARs preferentially, interacting with retinoic acid response elements (RAREs) to influence cellular response by forming homodimers or heterodimers with RARs [100]. For the *RAR-α* gene level, compound **4b** was the only synthetic retinoid able to induce a 2.9 ± 0.1-fold change with a significant increase compared to ATRA. This may explain the potency of compound **4b** since activation of RAR-α was demonstrated to inhibit cell growth, induce G1 arrest, and stimulate apoptosis [101]. For the *RAR-β* gene level, ATRA was the only retinoid able to induce a 35.3 ± 1.6 -fold change overexpression, and not for any other synthetic retinoids. For *RAR-γ* gene level, all synthetic compounds were able to induce gene overexpression except **4a** in a manner similar to ATRA without a significant difference (**3a**; 4.5 ± 0.2, **3b**; 2.4 ± 0.1, **4b**; 4.3 ± 0.2 and ATRA; 4.1 ± 0.2) (Fig. 6c). *RAR-γ* modulation is associated in human colorectal cancer with increased cell proliferation, rapid tumor progression, and a poor prognosis [102, 103]. Hence, *RAR-γ* plays a role in retinoid-induced apoptosis. To conclude, the gene expression data may suggest the retinoid-like activity and potency of compound **4b** to induce both *Caspase-3* and *Cyt-C* in a manner similar to ATRA. Moreover, compound **4b** was able to induce overexpression of both *RAR-α* and *RAR-γ*.

#### Western Blotting Analysis of RARs Proteins

**Fig. 7:**
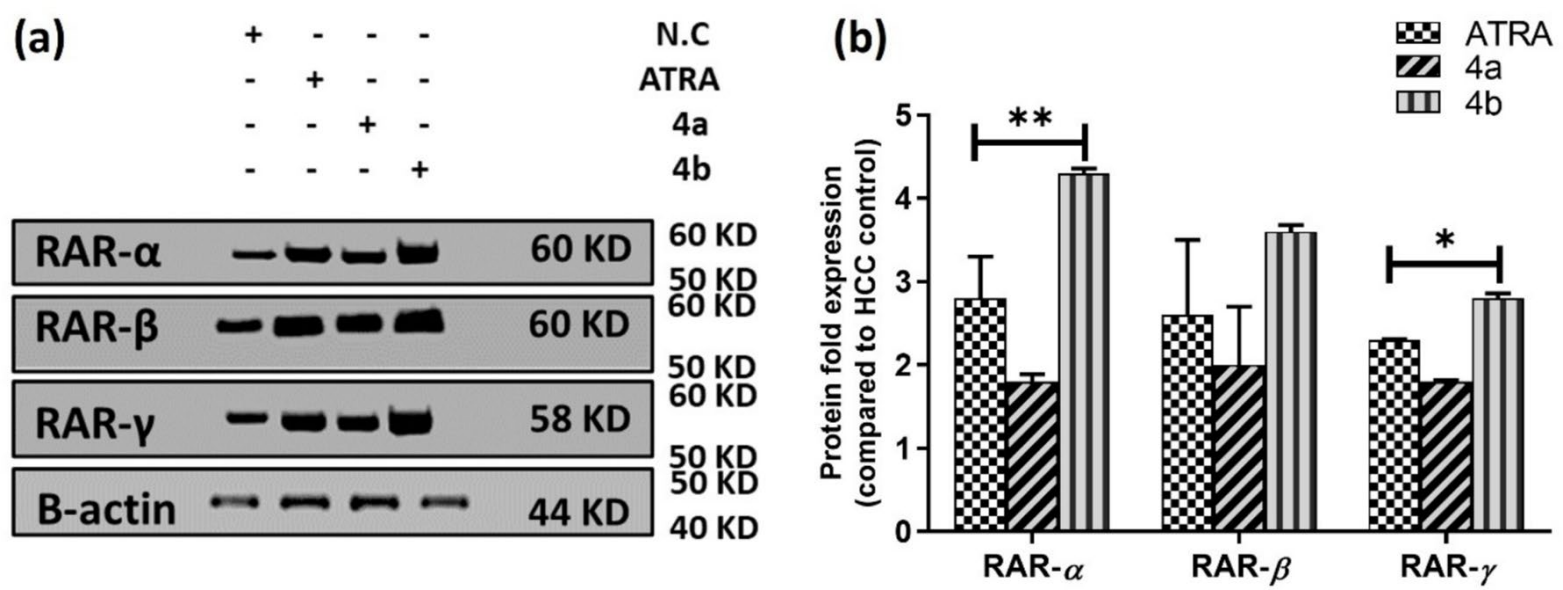
Western blotting analysis of RAR proteins in Caco-2 cells. (**A**) Representative immunoblotting images demonstrate the effect of the IC_50_ dose of ATRA, compounds **4a** and **4b**, on the protein expression levels of RARs in Caco-2 cells treated for 24 h. (**B**) Quantification of the tested proteins in Caco-2 cell lysates, with normalization to the β-actin protein. The expression of all recorded proteins in the control group is set to (“1”), and all data from three separate experiments are the fold change of protein expression to control and shown as mean ± SEM. * *p* < 0.05, ** *p* < 0.01 indicates a statistically significant difference from the matching ATRA group. The RAR protein blots were cropped from the original image according to their molecular weights (RAR-α was cropped between 50–60 KD, RAR-β was cropped between 50–60 KD, RAR-γ was cropped between 50–60 KD and, β-actin was cropped between 40–50 KD). The expected molecular weights for proteins are RAR-α: 60 KD, RAR-β: 60 KD, RAR-γ: 58 KD, and β-actin: 44 KD. The full-length blots/gels are presented in supplementary figures as follows: RAR-α: Fig. 2-4S, RAR-β: Fig. 5-7S, RAR-γ: Fig. 8-10S and β-actin: Fig. 11-13S. The Gel documentation system (Geldoc-it, UVP, England) was applied for data analysis using Totallab analysis software, ww.totallab.com, (Ver.1.0.1). (color is not required in print)

**Fig. 8:**
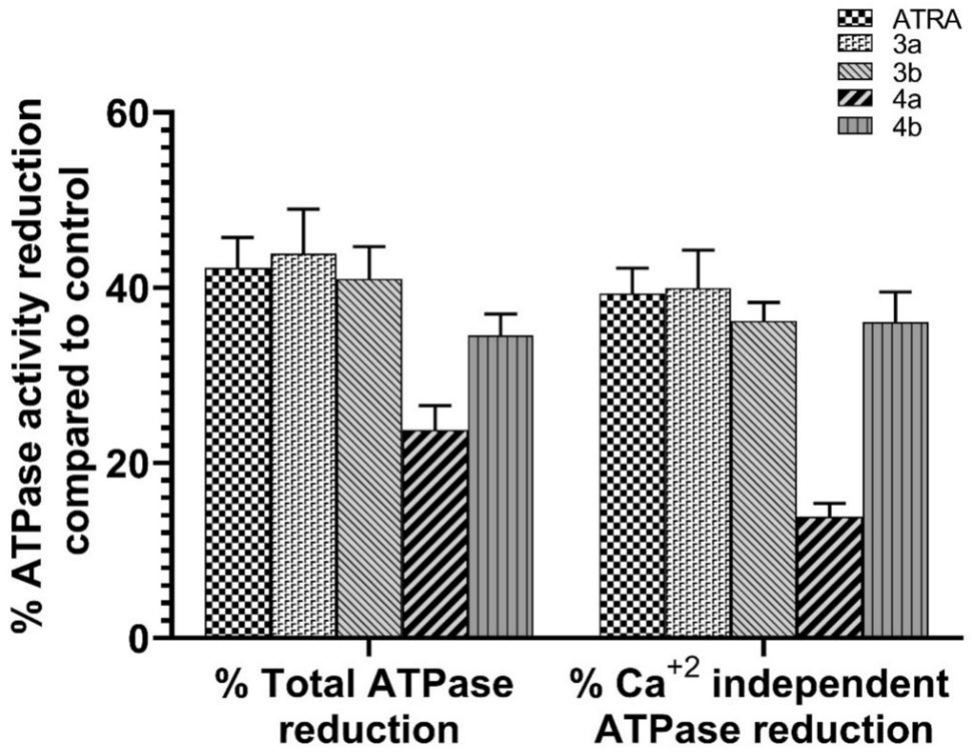
The total activity of ATPase and calcium-independent ATPases after the exposure of Caco-2 cells to new synthetic retinoids. Caco-2 cells were treated with the IC_50_ concentration of retinoids for 24 hs, and the enzymatic activity was measured colorimetrically. The ATPase activity observed in 0.1% DMSO control was normalized to 100%. The activity in the presence of synthetic retinoids was then detected and compared to the control and ATRA. Shown is the mean change in ATP-degrading activity ± SEM of three independent experiments (*n* = 3) (color is not required in print)

**Fig. 9:**
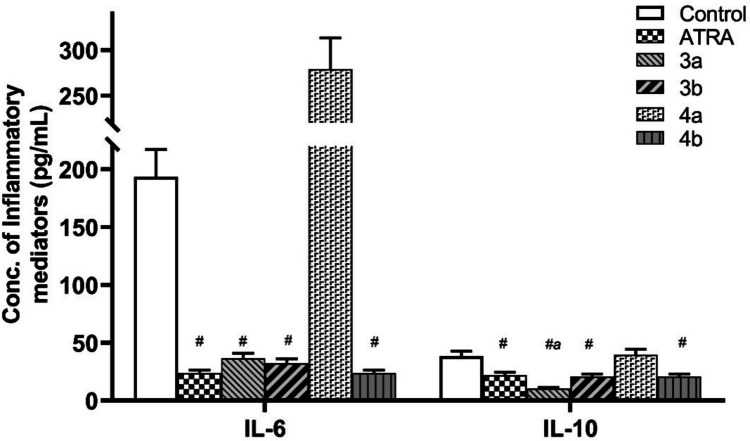
Effect of the new synthetic retinoids using their IC_50_ dose for 24 h on intracellular IL-6 and IL-10 expression levels in Caco-2 cells. Shown is the mean change in concentration of inflammatory mediators ± SEM of three independent experiments (*n* = 3) ^#^ indicate significance against negative control, ^a^ indicate significance against ATRA (color is not required in print)

**Fig. 10:**
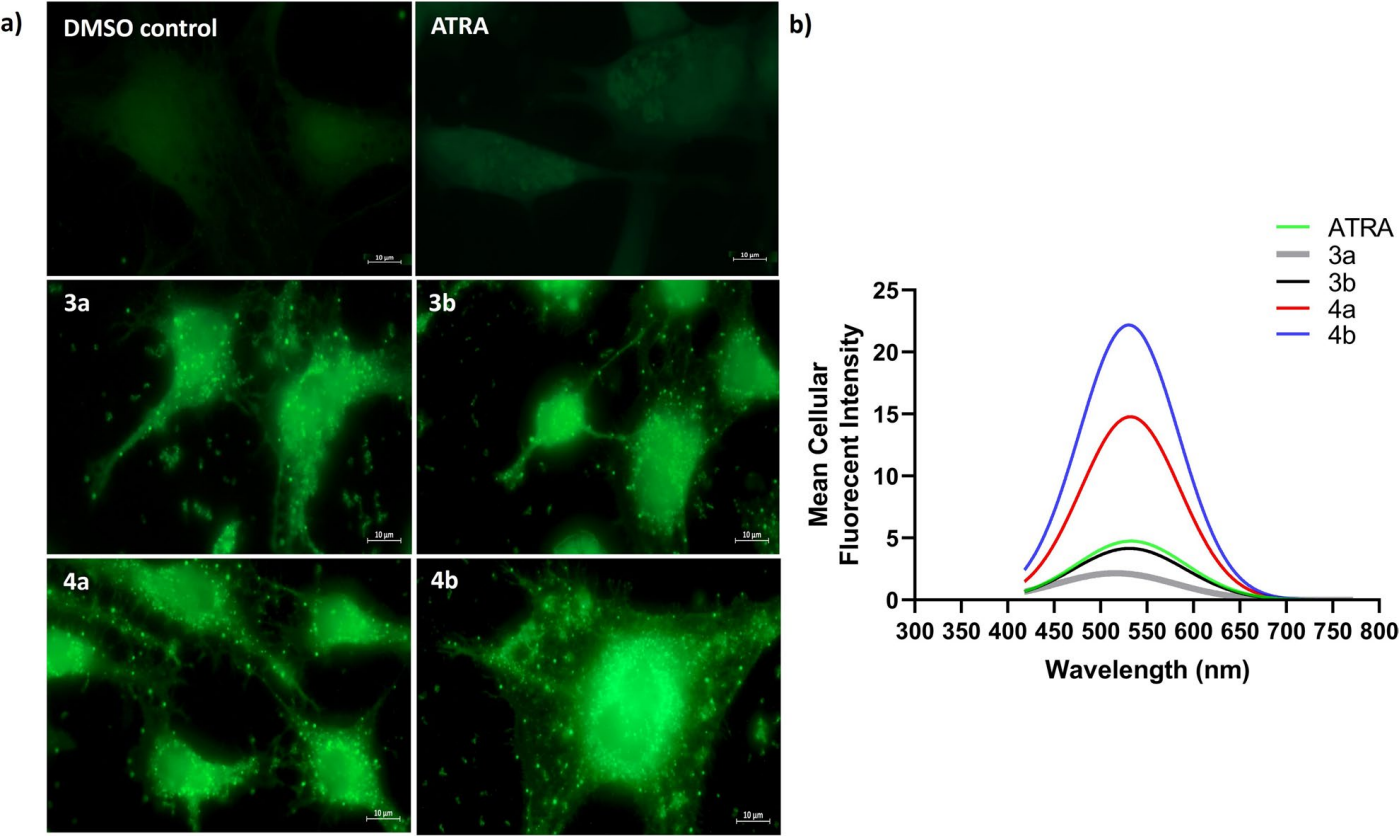
Intracellular fluorescent characterization of Caco-2 cells treated with **3a**, **3b**, **4a**, **4b**, and ATRA for 6 h at their IC_50_ dose by cell fluorescence emission scanning. A confocal microscope was used to record the emission using a lambda scan and a 405 nm laser for excitation (color is not required in print)

**Fig. 11:**
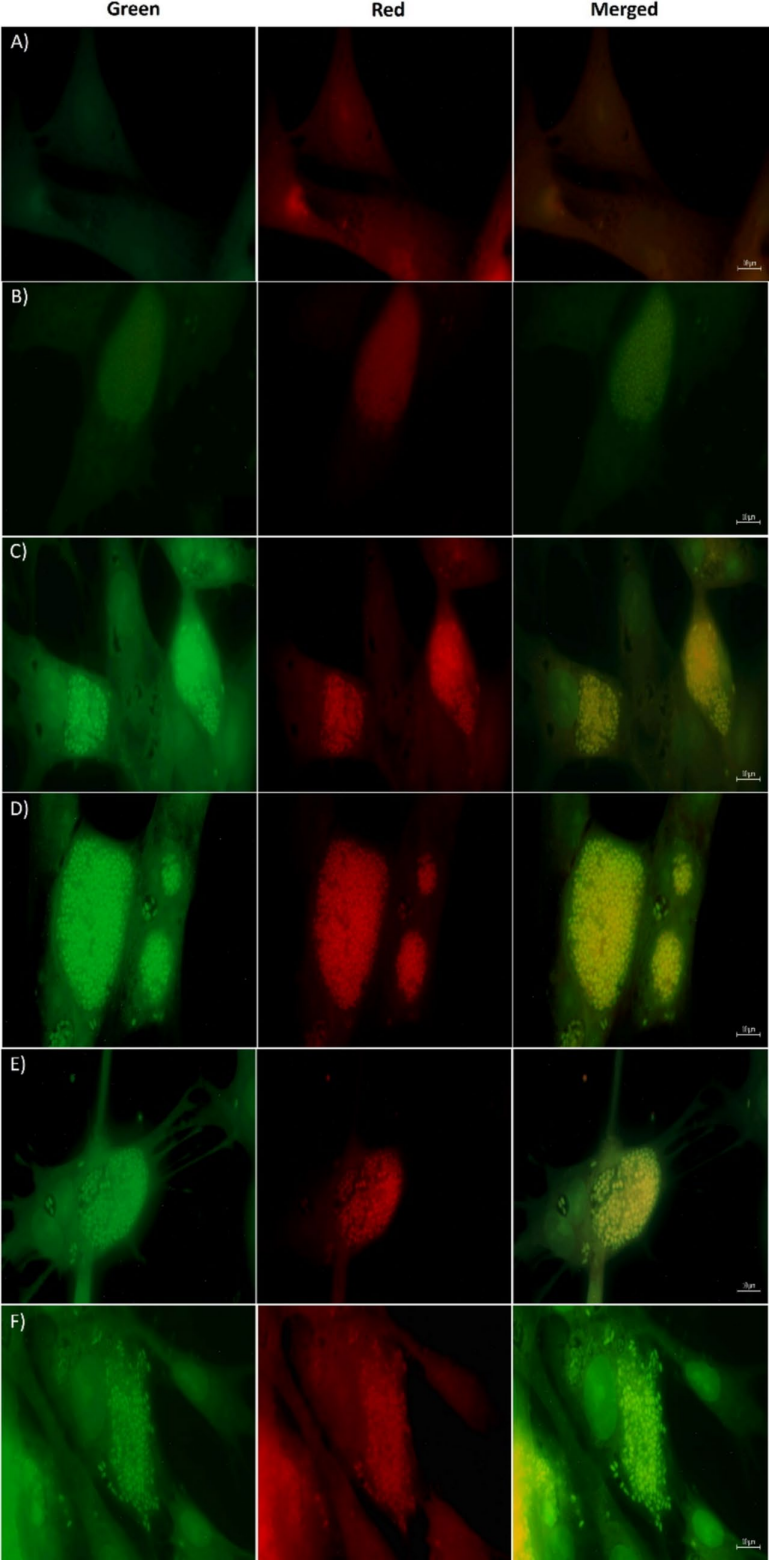
Co-localization images of new synthetic retinoids (**3a**, **3b**, **4a**, **4b**), and ATRA with Nile Red stain. **A**) DMSO control, **B**) ATRA, **C**) **3a**, **D**) **3b**, **E**) **4a** and, **F**) **4b**. *In-silico* evaluation (color is not required in print)

**Fig. 12:**
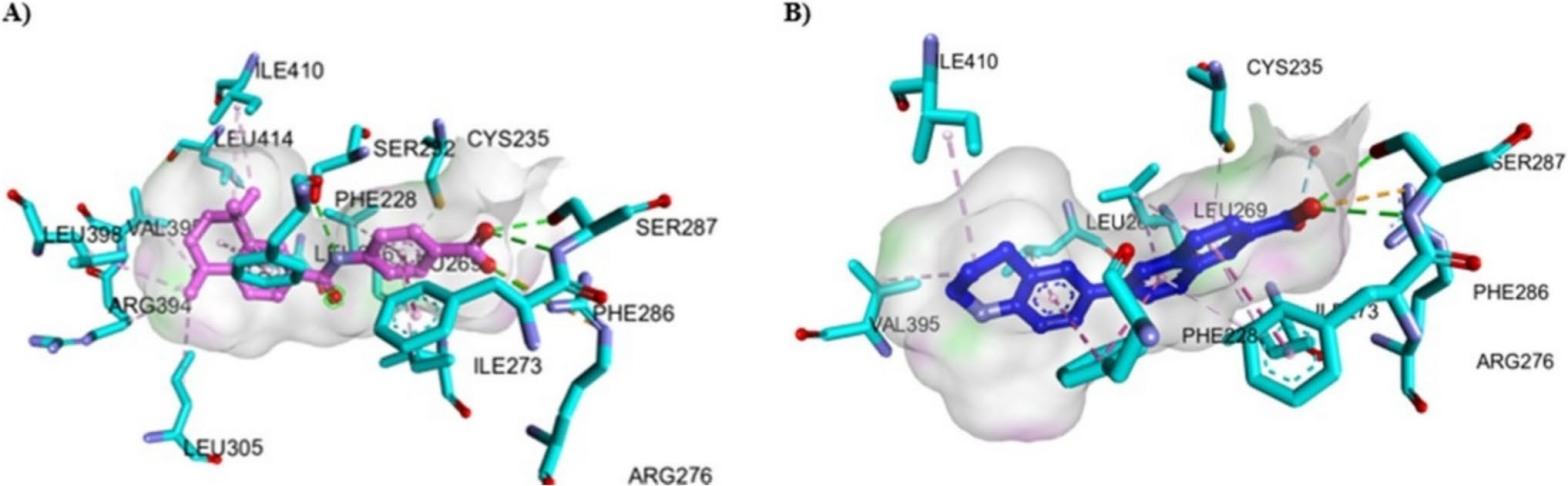
Represented the 3D and 2D binding modes of **A)** ATRA, and **B)** compound **4a** inside the RAR-α binding pocket (PDB: 3 KMR)

**Fig. 13:**
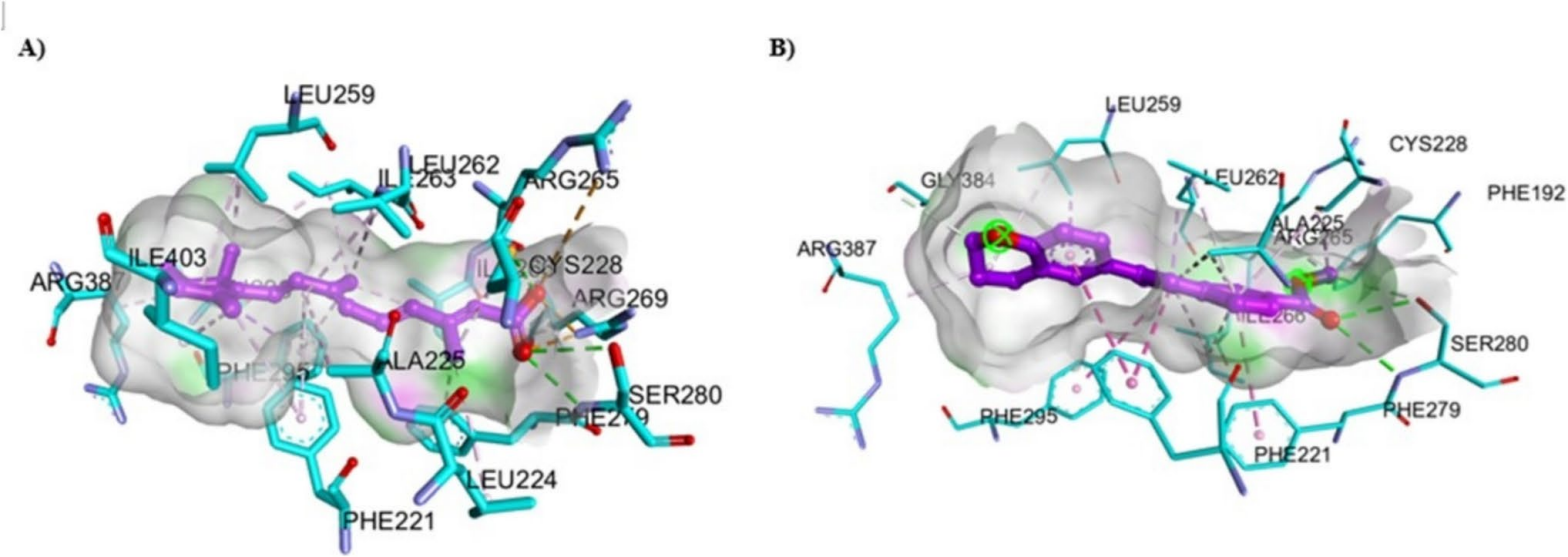
Represented the **3D** and **2D** binding modes of **A)** ATRA, and **B)** compound **3b** inside the RAR-β binding pocket (PDB: 1XAP) (color not required in print)

To determine the effects of the tested compounds in the Caco-2 cell line on the protein level, two compounds with variable *RAR* gene expression were selected for further RAR proteins assessment using western blotting **(**Fig. 7, supplementary data Fig. 2-13S). Compound **4b** showed overexpression of all RAR subtypes **(**Fig. 7a**)** with a significant difference for RAR-α and RAR-γ compared to ATRA as a positive control (Fig. 7b), consistent with the gene expression profile of *RAR* genes. Compound **4a** showed overexpression of all RARs (Fig. 7a), similar to ATRA (Fig. 7b). RAR proteins are transcription factors that bind to specific regions of DNA and help modulate the activity of different key genes by activation or repression [104]

#### New Synthetic Retinoids Interfere with the ATP-Catabolizing Activity of Calcium-Independent ATPases in Caco-2 Cells

ATPases is a part of the P-type pump superfamily, crucial for preserving low, nanomolar levels of cytoplasmic Ca^+2^ during rest and carcinogenesis [105]. There is growing evidence that dysregulated Ca^+2^-ATPase expression plays a significant role in breast, colon, lung, and other types of malignancies [106, 107]. The tumor may use these enzymes as unique targets for therapeutic interventions or as indicators of distinction. Therefore, the whole lysate of Caco-2 was used to study the impact of a single IC_50_ dose of new synthetic retinoids after 24 h on the activity of total calcium-independent ATPases. The amount of inorganic phosphate (Pi) released from the ATPase enzymes after new synthetic retinoid treatment was measured and compared to both the DMSO control and the single IC_50_ dose of parent ATRA as a positive control after 24 h. Data showed that all new synthetic retinoids were able to reduce ATPase activity in a way comparable to ATRA (**3a**; 43.9%, **3b**; 41%, **4b**; 34.6%, and ATRA; 42.2%), and compound **4a** showed the superior activity in the reduction of ATPase activity to (23.7%) (Fig. 8).

Furthermore, the ca^+2^- independent ATPase activity was markedly decreased after applying a treatment with the new synthetic retinoids in the same way as ATRA, and compound **4a** had the highest percentage of reduction in activity (**3a**; 40.0%, **3b**; 36.2%, **4a**; 13.9, **4b**; 36.1%, and ATRA; 39.4%) (Fig. 8). ATP is essential for all living cells, and recently there is a correlation between intracellular ATP level and apoptosis activation. The range of ATP depletion serves as a threshold for identifying the cause of cell death. Severe ATP depletion (less than approximately 15% of control) results in uniform cell death by necrosis, while moderate ATP depletion (greater than approximately 25% of control) causes cell death by apoptosis [108]. Also, ATP depletion often leads to reactive oxygen species generation that induces apoptotic cascade and caspase-3 activation; hence, ATPase has anti-apoptotic and carcinogenic functions, and inhibiting it has been identified as a possible treatment option for cancer [109]. Additionally, ATPase alterations in its expression and activity may have a role in the pathogenesis of different cancer types, such as invasiveness, metastasis, and multidrug resistance; therefore, this pump is a promising target for treatment [110].

#### Determination of Intracellular IL-6 and IL-10 Proteins Level

The presence of inflammation and its function in cancer are indicated by the identification of the characteristic pro-inflammatory cytokines, IL-10, and IL-6 [111]. The overall anti-inflammatory action is partially attributed to the decrease in intracellular levels of these inflammatory indicators and hence carcinogenesis. In this study, intracellular concentrations of both IL-10 and IL-6 after implanting a treatment with IC_50_ of new synthetic retinoid compounds **(3a, 3b, 4a**, and **4b)** for 24 h were measured and compared to both “DMSO” negative control and ATRA positive control (Fig. 9). All tested compounds showed a reduction in intracellular concentrations of IL-6 (% inhibition = 23.7–36.6%) and IL-10 (% inhibition = 19.640–61.561%) while compound **4a** did not show any significant reduction in both interleukins’ levels. Compound **3a** showed additional significant reduction in IL-10 level compared to ATRA (Fig. 9). There is a remarkable correlation between apoptosis induction and the anti-inflammatory activity of retinoids. Activation of caspases aids in the disassembly and packaging of cellular constituents into membrane-bound apoptotic bodies during apoptosis [112]. These apoptotic structures stop cells from leaking their harmful contents into the surrounding environment; thus, cells become hyporesponsive to outside stimuli and diminish their pro-inflammatory potential and oxidative stress. This facilitates the removal of non-phlogistically from an inflammatory site in a process called efferocytosis [113]. Additionally, the elimination of apoptotic cells is sufficient to stop secondary necrosis and release cell contents that might cause inflammation.

### Fluorescent Characterization

#### Cellular Photoimaging Characterization

The next step was to characterize the fluorescent activity of these new synthetic retinoids using some imaging studies to assess and understand the compounds'cellular photoactivity and localization and to correlate their behaviors with the structural variations. Following 6 h of incubation at the IC_50_ dose of each compound, Caco-2 cells were imaged using Carl Zeiss LSM 710 confocal microscopes. DMSO negative control showed minimal auto-fluorescence activity while all new synthetic retinoids were able to penetrate the plasma membrane and enter the cell spontaneously, distributed and accumulated in both the cell membrane and cytoplasm of the Caco-2 cells with remarkable green fluorescence (Fig. 10a) much more intense than ATRA as a positive control. All new synthetic fluorescent retinoids were observed in the cytoplasm and most likely in lipid-like areas, such as lysosomal vesicles, the nucleus, and non-polar cell membrane, suggesting that it might localize nonspecifically due to its extremely lipophilic nature. This may suggest these new molecules can be used as an intracellular probe for bioimaging. The whole cell fluorescence emission scan (lambda scan) study was performed at wavelength (300–700 nm) with each compound carefully to minimize photobleaching. Each molecule exhibited a wide emission spectrum typical of the donor–acceptor excitation state that gives typical emission spectra for each molecule, as shown in Fig. 10b, which illustrates the most notable distinctions between the compounds with maximum emission at ~ 525 nm. The most notable is the blue-green shift in the acids **4a**/**4b**'s emission at higher mean cellular fluorescence intensity when compared to the equivalent esters **3a**/**3b,** and this was compared to ATRA in acid form. The acid form of tetrahydroquinoline and chroman derivatives exhibited higher fluorescence intensity emission spectra than their corresponding esters, which is consistent with the hypothesis that acids can initiate and propagate resonance more efficiently than their corresponding esters at physiological pH due to shifting to a more polar environment [114]. Moreover, the fluorescent activity of the new synthetic retinoids may have some implications on the anticancer activity observed and especially cell death induction due to the formation of a specific reactive oxygen species (ROS) upon photoactivation [115]. The irradiation or light-activated effect from the external environment is sufficient to induce strong electron movement from π-donor and extended to π-acceptor moieties to induce a bathochromic shift with a movement from the S_0_ ground state to the S_1_ excited state, inducing the formation of ROS and cell death [116]. The length of the molecules of new synthetic retinoids fits with retinoid structure criteria. Hence, the expected biological activity may be mediated dually through the induction of retinoid signaling modulations as well as the photo-activating apoptosis effect [19].

#### Fluorescent Co-Localization

After understanding the fluorescent behavior over the entire cell, the researchers performed an imaging study to learn more about how the structure of the drug affected the localization behavior. Caco-2 cells treated with compounds **3a**, **3b**, **4a**, **4b**, and ATRA as a positive control were co-treated with commercially available Nile red as a general lipophilic stain with absorption/emission characteristics as a part of the co-localization strategy. Figure 11 illustrates that new green, fluorescent retinoids were able to localize into the cell membrane, cytoplasm, and nucleus. Nile red stain and the new synthetic retinoids were able to localize in the same cellular area of the endoplasmic reticulum (ER), characterized as non-polar vesicles containing oil droplets as shown in the merged images (Fig. 11). This may suggest the lipophilic tendency of the new retinoid compounds as well as their localization in ER due to cellular uptake by endocytosis and being ready for any cellular release.

### In-Silico Evaluation

#### In-Silico ADME and Toxicity Predictions (Drug Likeness and Medicinal Chemistry Prediction)

The Swiss ADME online tool (http://swissadme.ch/index.php) was used to analyze the *in-silico* computational evaluation for new synthetic retinoids and the ATRA positive control as previously described. Table 5 displays the outcomes of the expected parameters, which include molecular characteristics, pharmacokinetics, drug-likeness, and medicinal chemistry [117]. The outcome showed that all synthetic retinoids adhere to the Veber and Lipinski rules without breaking any of them. The only exception to this is ATRA, which broke the rule one time due to *Log P* being greater than 4, indicating that ATRA has high metabolic turnover and metabolism, low solubility, and poor oral absorption [118]. For new fluorescent synthetic retinoids, there are enough increases in *log P* due to the hydrophobic moiety and ester group's presence to result in some hydrophobic interactions with more lipophilic cellular compartments. Furthermore, all the synthetic retinoids and ATRA showed moderate soluble tendencies in aqueous solvents, with log S (ESOL) values varying between −5.06 and −5.34. Furthermore, all synthetic retinoids and ATRA revealed no alarms for pan-assay interference compounds (PAINs) in their structure.

**Table 5:** Prediction of molecular properties, pharmacokinetics, drug-likeness, and medicinal chemistry of new synthetic retinoids (**3a**, **3b**, **4a**, and **4b**) and ATRA using the Swiss ADME online tool

Retinoids derivatives and positive control ATRA						
Test items		ATRA	3a	3b	4a	4b
Swiss ADME	Molecular properties					
	M *log P*	4.28	3.89	3.89	3.66	3.66
	TPSA (Å2)	37.30	38.33	35.53	49.33	46.53
	M. Wt	300.44	317.38	318.37	303.35	304.34
	nHBA (NO)	2	2	3	2	3
	nHBD (OHNH)	1	1	0	2	1
	NRB	5	3	3	2	2
	Pharmacokinetics					
	GI absorption	High	High	High	High	High
	BBB permeant	Yes	Yes	Yes	Yes	Yes
	P-gp substrate	No	No	No	Yes	Yes
	Skin penetration (log Kp) cm s − 1	−3.66	4.66	−4.68	−4.81	−4.83
	Drug likeness and medicinal chemistry					
	log S (ESOL)	−5.34	−5.27	−5.27	−5.06	−5.06
	Solubility	Moderate	Moderate	Moderate	Moderate	Moderate
	PAINS	0	0	0	0	0
	Synthetic accessibility	4.1	2.29	2.57	2.14	2.42
	Bioavailability score	0.85	0.55	0.55	0.85	0.85
	Lipinski rule (violation)	Yes (1)	Yes (0)	Yes (0)	Yes (0)	Yes (0)
	Veber rule (violation)	Yes (0)	Yes (0)	Yes (0)	Yes (0)	Yes (0)

Additionally, the evaluated derivatives and positive control showed respectable bioavailability ratings, with the ATRA value being 0.85 and the remaining derivatives being 0.55. Furthermore, all synthetic retinoids demonstrated easy synthetic accessibility in the range of 2.14–2.42, which is less than that of ATRA (4.1). Also, all synthetic retinoids showed high GI absorption and BBB clearance, which suggests the best potential use of these derivatives as drugs due to the improvement of pharmaceutical (drug form and its release), pharmacokinetic (drug transport), and pharmacodynamic phase (interaction with the receptor) [119]. Moreover, it was shown that synthetic compounds and ATRA are not substrates for P-glycoprotein (P-gp) except for compound **4a**, which is reliable for drug excretion. One of the main contributors to the multidrug-resistant phenotype in cancer is P-gp by limiting the amount of anticancer drugs available for cytotoxic and apoptotic effects [120].

#### Toxicological Studies

Protox II (https://toxnew.charite.de/protox_II/) was used to forecast the toxicity of the most active molecules. Using Protox II as a 2D-computational toxicity prediction model can help to reduce the number of animal experiments, materials used, and testing costs. Table 6 shows that the median lethal dose (LD_50_ = 1000–2188 mg.kg^−1^, class IV-V) for the new synthetic retinoids is high, suggesting their lower animal toxicity and potential use for *in-vivo* studies, as it may be harmful if ingested orally in doses (300 < LD_50_ ≤ 2000) according to Hazard Communication Standard [121].

**Table 6:** *In-silico* toxicity prediction of the new synthetic retinoid derivatives using ProTox II prediction

New synthetic Retinoids derivatives					
Oral toxicity prediction		3a	3b	4a	4b
ProTox II prediction	**LD**_**50**_** mg.kg**^**−1**^	2188	1460	1000	1460
	Toxicity class	V	IV	IV	IV
	Hepatotoxicity (*p* value)	Active 0.66	Inactive 0.72	Inactive 0.68	Inactive 0.68
	Carcinogenicity (*p* value)	Inactive 0.65	Active 0.54	Inactive 0.60	Active 0.53
	Immunotoxicity (*p* value)	Inactive 0.57	Inactive 0.76	Inactive 0.99	Inactive 0.95
	Mutagenicity (*p* value)	Inactive 0.76	Inactive 0.70	Inactive 0.69	Inactive 0.75
	Cytotoxicity (*p* value)	Inactive 0.83	Inactive 0.82	Inactive 0.59	Inactive 0.71

The main obstacle in using any previous synthetic retinoid analogue is the low therapeutic: Toxic ratios and high pharmacologic dosages are necessary, which results in side effects often associated with the drug's mechanism of action [122]. However, the current new synthetic retinoids have relatively higher LD_50_ doses, suggesting their safety margin.

As a result, throughout the preclinical or clinical stages, medication safety is among the main reasons for many synthetic retinoid withdrawals, especially due to liver toxicity [123]. Herein, the majority of new synthetic retinoids showed minimal activity in the five toxicity classes: **3a** (Hepatotoxicity), **3b**, and **4b** (Carcinogenicity). Based on these data, new synthetic retinoids showed good LD_50_ values and toxicity class IV-V, and they did not contain any of the known specific toxic fragments, such as benzimidazole, perfluoroterephthalonitrile, 4,5,7-trichloro-6-nitro-2-(trifluoromethyl), and chloroflurazole [124] demonstrating a relatively non-toxic profile on organ toxicity and toxicity endpoints for promising testing in *In-vivo* studies.

#### Molecular Docking Simulation

**Fig. 14:**
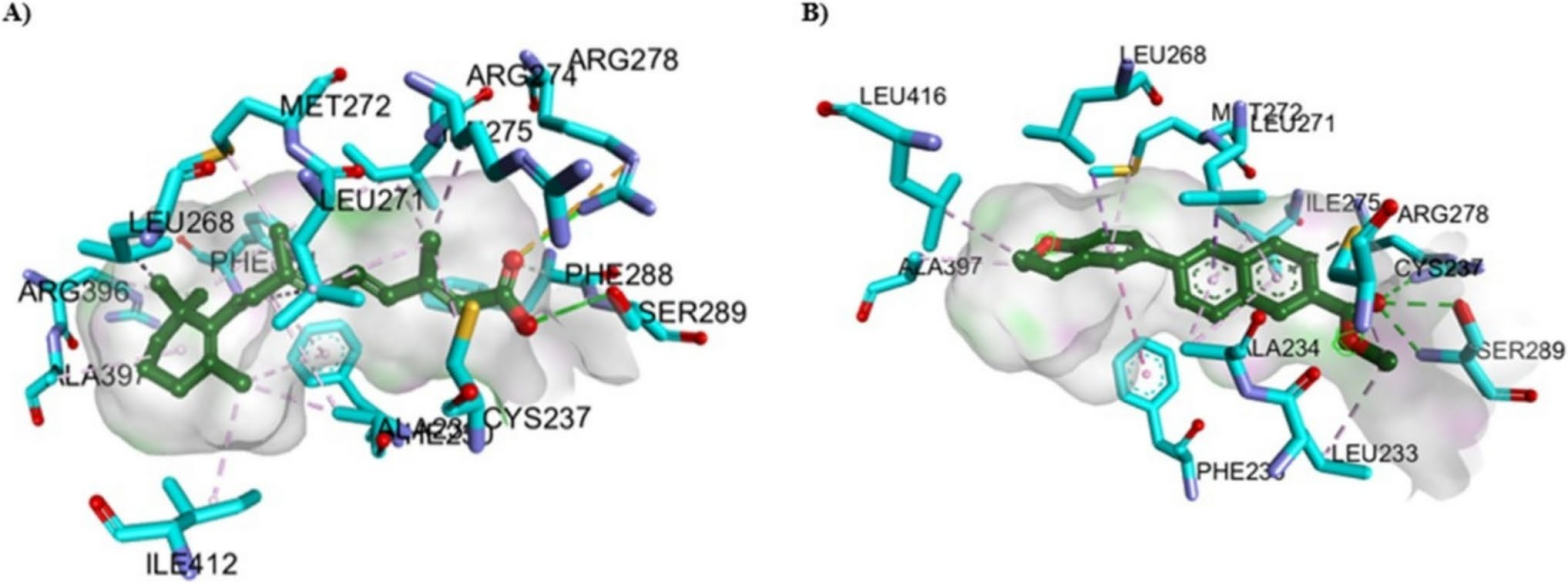
Represented the **3D** and **2D** binding modes of **A)** ATRA, and **B)** compound **3b** inside the RAR-γ binding pocket (PDB: 2LBD) (color not required in print)

The molecular docking simulation for ATRA and new synthetic retinoids was performed in the ligand binding pocket of RAR-α (PDB = 3 KMR), RAR-β (PDB = 1XAP), and RAR-γ (PDB = 2LBD), retrieved from Protein Data Bank (https://www.rcsb.org/), in order to understand the appropriate anticancer mechanism activity and explain the experimental result obtained previously. These PDB IDs for the crystal structures were selected based on the best values of Resolution (RAR-α; 1.80, RAR-β; 2.10, and RAR-γ; 2.06 Å), R-Value Free (RAR-α; 0.237, RAR-β; 0.253, and RAR-γ; 0.313), R-Value Work (RAR-α; 0.197, RAR-β; 0.213, and RAR-γ; 0.210), and R-Value Observed (RAR-α; 0.199, RAR-β; 0.213, and RAR-γ; 0.210). These parameters help to ensure the most ordered portions of the crystal structures of RARs, a fitted atomic model of the electron density map, and minimal uncertainty of atomic positions. First, the validation procedure was performed by the redocking process of the co-crystallized ligands in the different RARs (RAR-α: AM580, RAR-β: TTNPB, and RAR-γ: ATRA) with their proteins using the docking protocol, and data of binding energy (S) in kcal/mol, RMSD score in (Å), and interacting amino acid residues were shown (Table 7 and Fig. 14S). For RAR-α, the AM580 standard reference showed S = −13.352 kcal/mol with RMSD = 1.109 Å and several hydrogen bonding interactions through the carboxylate group of AM580 with Ser232 and Arg276. Also, AM580 showed hydrophobic interaction between the stacked layer of the phenyl group with Phe228.

**Table 7:** Molecular docking analysis of ATRA and new synthetic retinoids in the ligand binding pockets of different types of RARs in comparison to the original co-crystalized ligands using MOE docking software

Compounds	Binding energy (S) (kcal/mol)	RMSD Score (Å)	Interacting amino acids	
			Hydrogen bonding	Hydrophobic forces
Docking results with RAR-α (PDB = 3 KMR)				
Docking of crystalized structure (AM580) (For validation)	−13.352	1.109	1. Ser232 = 3.22 2. Ser287 = 2.00 3. Ser287 = 1.99 4. Ser287 = 2.32 5. Arg272 Through H_2_O = 1.99 and 2.28 6. Arg276 = 2.08	1. Phe228 ~ 3.60
ATRA	−11.659	0.612	1. Ser287 = 2.31 2. Ser287 = 2.21 3. Arg272 Through H_2_O = 2.09 and 2.28 4. Arg276 = 2.32 5. Arg276 = 2.43	1. Phe228 ~ 3.60
3a	−8.219	0.818	1. Ser287 = 2.33 2. Ser287 = 2.17 3. Ser287 = 2.59 4. Arg272 Through H_2_O = 2.00 and 2.28	1. Phe228 ~ 3.95
3b	−8.066	0.510	1. Arg276 = 2.58 2. Arg272 Through H_2_O = 1.99 and 2.28	1. Phe228 ~ 3.49
4a	−7.815	0.761	1. Ser287 = 2.31 2. Ser287 = 1.98 3. Arg272 Through H_2_O = 2.03 and 2.28 4. Arg276 = 2.67 5. Arg276 = 2.66	1. Phe228 ~ 3.53
4b	−8.207	1.010	1. Ser287 = 2.20 2. Ser287 = 2.02 3. Arg272 Through H_2_O = 2.00 and 2.28 4. Arg276 = 2.52	1. Phe228 ~ 3.53
Docking results with RAR-β (PDB = 1XAP)				
Docking of crystalized structure (TTNPB) (For validation)	−12.121	1.192	1. Arg269 = 2.15 2. Ser280 = 2.10 3. Ser280 = 1.87	1. Leu259 ~ 3.83
ATRA	−11.506	0.774	1. Arg269 = 2.41 2. Arg269 = 2.45 3. Ser280 = 2.40 4. Ser280 = 2.14	1. Phe295 ~ 4.94
3a	−8.807	1.140	1. Ser280 = 2.47 2. Ser280 = 2.30	1. Leu259 ~ 3.88 2. Leu262 ~ 3.72 3. Leu262 ~ 4.02 4. Leu262 ~ 4.36
3b	−8.675	1.103	1. Ser280 = 2.46 2. Ser280 = 2.29	1. Leu262 ~ 4.2 2. Leu262 ~ 4.46
4a	−9.213	1.025	1. Arg269 = 2.63 2. Ser280 = 2.20 3. Ser280 = 2.11	1. Leu259 ~ 3.85 2. Leu262 ~ 4.00
4b	−8.883	1.332	1. Arg269 = 2.57 2. Ser280 = 2.26 3. Ser280 = 2.07	1. Leu259 ~ 3.75 2. Leu262 ~ 3.99
Docking results with RAR-γ (PDB = 2LBD)				
Docking of crystalized structure (ATRA) (For validation)	−11.666	1.102	1. Arg278 = 2.14 2. Arg278 = 2.45 3. Ser289 = 1.99 4. Ser289 = 2.37	1. Phe304 ~ 4.94
3a	−8.668	0.952	1. Arg278 = 2.14 2. Arg278 = 2.45 3. Ser289 = 1.77 4. Ser289 = 2.25	1. Leu271 = 3.13
3b	−8.574	0.532	1. Arg278 = 1.72 2. Ser289 = 2.19 3. Ser289 = 2.38	1. Leu271 = 3.14 2. Phe304 ~ 3.53
4a	−9.003	1.063	1. Arg278 = 1.82 2. Ser280 = 1.93 and 2.38 3. Ser280 = 2.32	1. Leu271 = 3.10 2. Met415 = 4.00 3. Phe304 ~ 4.02
4b	−8.756	0.650	1. Arg278 = 1.82 2. Ser280 = 1.96 and 2.33 3. Ser280 = 2.32	1. Leu271 = 3.11 2. Met415 = 4.9 3. Phe304 ~ 4.59

**Fig. 15:**
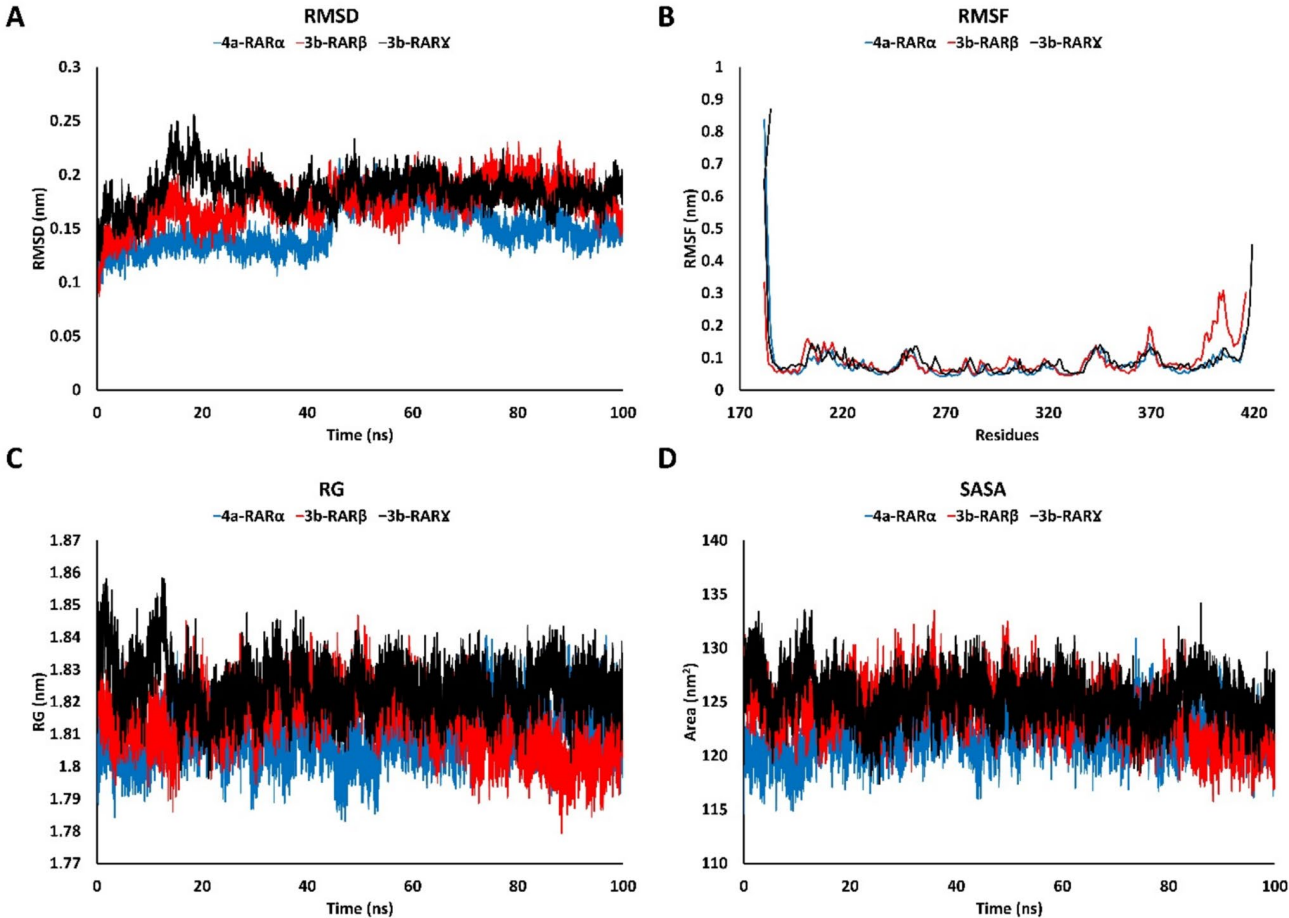
Molecular dynamic simulation results of the complexes **4a**-RAR-α, **3b**-RAR-β, and **3b**-RAR-γ. **A**) RMSD plot of the backbone atoms of the proteins complexed with the different compounds. **B**) RMSF plot of the backbone atoms of the proteins complexed with the different compounds. **C**) RG plot, and **D**) SASA plot

ATRA binds at the same pocket of AM580 with S = −11.659 kcal/mol and a lower RMSD score (0.612 Å). In addition, all new synthetic retinoids showed comparable binding energy for RAR-α ranging from −8.219 to −7.815 kcal/mol and RMSD score (0.5097 to 1.0099 Å) with typical hydrogen bonding and phenyl-stacking interactions, compared to the positive control ATRA and AM580 (Fig. 12 and Fig. 15S).

Regarding RAR-β, redocking the co-crystallized ligand TTNPB showed S = −12.121 kcal/mol and RMSD = 1.109 Å with hydrogen bonding between the carboxylate group of TTNPB and key amino acid residues Ser280 and Arg269. Also, hydrophobic van der Walls forces were observed with Leu259 (Table 7 and Online resource 2). ATRA showed binding energy with S = −11.506 kcal/mol and RMSD = 0.612 Å and typical hydrogen bonding interaction and hydrophobic interaction with Phe295. The new synthetic retinoids occupied the same binding pocket as a co-crystallized ligand with binding energy −9.213 to −8.675 kcal/mol with an RMSD score (1.025 to 1.332 Å), respectively. New synthetic retinoids showed additional hydrophobic interactions with both Leu259 and Leu262 in the RAR-β binding pocket (Fig. 13 and Online resource 3).

Finally, for RAR-γ, edocking the co-crystalized ligand completely overlaid the crystallized molecules with a binding energy of −11.666 kcal/mol and RMSD = 1.102 Å with hydrogen bonding between the carboxylate group and key amino acid residues Ser289 and Arg278 in the RAR-γ binding pocket (Table 7 and Online resource 1). The new synthetic retinoids showed a promising binding affinity with an S-value ranging from −9.003 to −8.574 kcal/mol, and the RMSD score ranged from 0.532 to 1.063. The new synthetic retinoids showed hydrophobic interactions with Phe304, Leu271, and Met415 in the RAR-γ binding pocket (Fig. 14 and Online resource 4).

Hence, the molecular docking study suggested RARs as potential molecular targets for the new synthetic retinoids, and ligands can act as modulators for RAR’s key regulatory genes. RARs are transcriptional regulators for several subsets of other genes in a ligand-dependent manner. Although studies using structural crystallography showed that the RAR LBDs'structures are quite similar, binding with retinoids induces ligand-binding pocket conformational changes [125]. The ligand binding pocket consists of the hydrophobic residues, originating mostly from helices H3, H5, H11, and the β-sheet [126]. The ligand binding pocket (LBP) maximizes hydrophobic interactions and enhances ligand binding selectivity by matching the ligand's volume with its shape [127]. Furthermore, there are some polar residues (Arg and Ser) responsible for ligand selectivity and high binding affinity via the formation of hydrogen bonding and salt bridge networks [128]. This was matched with our molecular docking data of the current new synthetic retinoids that bind to RARs subtypes through a network of hydrophilic and hydrophobic bonding. Several investigations showed that synthetic retinoid therapy that induces RAR genes or agonists to the proteins suppresses the proliferation of several cancer cell types rather than causing cell differentiation through different RAR-dependent and -independent pathways [22].

RAR-α increased ROS, which in turn aided in cell cycle arrest and the apoptotic process in cancer cells [129]. Elevated ROS triggers apoptosis in colorectal cancer cells by stimulating HIF-1α and IGFBP-3 proteins, which in turn triggers apoptosis and suppresses PI3 K/Akt/mTOR [130]. Additionally, ROS may damage proteins, nucleic acids, lipids, membranes, and organelles, all of which can trigger apoptosis [131]. Similarly, RAR-γ activation had a concentration- and time-dependent anti-proliferative impact through different mechanisms, including alteration in the expression of the Bcl-2/Bax ratio that induces apoptosis [132]. Also, RAR-β induction impacts the overexpression of several genes, such as *Waf1*/*Cip1*/*Sdi1*/*p21*, *chk1*, *p300*/*CBP*, *BAX*, *Bak*, *Apaf-1*, *Kip1*/*p27*, *caspase-3,* and *caspase-9* that all have growth-inhibitory effect on cancer cells [133]. Moreover, RAR activation was shown to downregulate cellular inflammatory mediators such as IL-6, TNF-α, IL-12, and PGE-2 and NF-κB activities, leading to reduced carcinogenesis, metastasis, and immune response [134, 135].

#### Molecular Dynamic Simulation

RMSD analysis was used to measure the structural stability of the three complexes, each involving a different compound (**4a** and **3b**) bound to three distinct isomers of the retinoic acid receptor (RAR-α, RAR-β, and RAR-γ) over the simulation time. From the plot in Fig. 15a, the complex **4a**-RARα (blue) exhibited the most stable conformation with the lowest RMSD values (average 0.15 ± 0.02 nm) throughout the simulation. This suggests that the **4a**-RAR-α complex maintained a stable conformation. The complex **3b**-RAR-β (red) also demonstrates significant stability, with an average RMSD of 0.17 ± 0.02 nm reflecting its structural stability. Lastly, the **3b**-RAR-γ complex (black) showed high fluctuations during the first 20 ns, where RMSD reached a peak of 0.25 nm, then started to stabilize with an average of 0.18 ± 0.01 nm for the remaining time. These observations suggest that compound **3b** induced structural changes in the RAR-γ receptor compared to the other complexes. Overall, the lower RMSD values of the **4a**-RAR-α, **3b**-RAR-β, and **3b**-RAR-γ are in the acceptable range for the stably formed complexes.

The RMSF plot (Fig. 15b) provides insight into the flexibility of individual residues within RAR across the three isomers (RAR-α, RAR-β, and RAR-γ) bound to the compounds (**4a** and **3b)** during the 100 ns simulation. In general, the majority of residues across all complexes exhibited low RMSF values, indicating relatively stable and minimal fluctuations, particularly in the middle regions (residues 220–370). However, the N-terminal and C-terminal regions displayed higher fluctuations, with peaks in flexibility seen for all three complexes. Compound **4a** with RAR-α (blue) maintained consistently lower fluctuations across most residues compared to the other complexes, suggesting a more rigid and stable interaction.

The Rg plot (Fig. 15c) illustrates the compactness of the three RAR isomers (RAR-α, RAR-β, and RAR-γ) in complex with the compounds (**4a** and **3b)** over the simulation period. In general, the Rg values for all complexes fluctuate around 1.81 ± 0.01 nm. These results suggest that the complexes maintained stable and compact structures throughout the simulation.

The SASA plot illustrated in Fig. 15d depicts the exposure of RAR-α, RAR-β, and RAR-γ to solvent in complexes with the compounds (**4a** and **3b)** over the course of the simulation. SASA values reflect how much of the protein–ligand complex is exposed to the solvent molecules, indicating the degree of interaction with the surrounding environment. In general, all three complexes exhibited fluctuating SASA values between 115 and 134 nm^2^, showing variations in solvent exposure over time. The **4a**-RAR-α complex (blue) consistently showed lower SASA values, around 122 ± 2 nm^2^, suggesting a more compact and less solvent-exposed structure throughout the simulation. The **3b**-RAR-β complex (red) and the **3b**-RAR-γ complex (black) demonstrated higher SASA values (averages 124 ± 2.5 and 125 ± 2.1 nm^2^, respectively), indicating increased solvent exposure and likely more flexibility in the protein–ligand interaction.

**Fig. 16:**
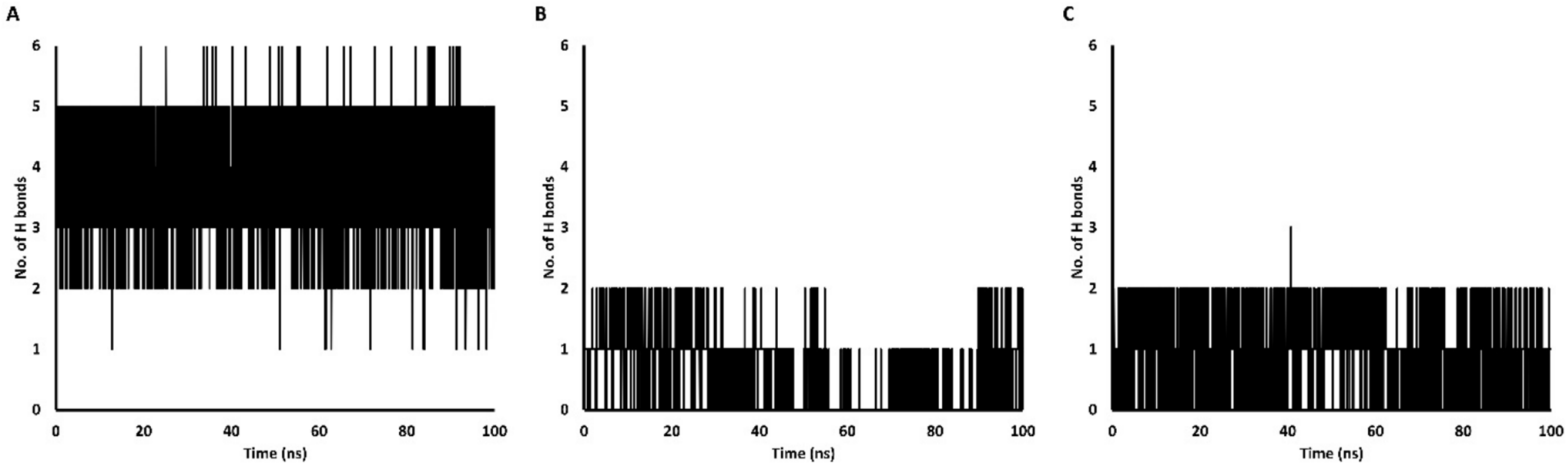
Hydrogen bond analysis of the compounds (**4a** and **3b**) with different RAR isomers over 100 ns of molecular dynamics simulation. **A**) Total number of hydrogen bonds formed between compound 4a and RARα. **B**) Total number of hydrogen bonds formed between compound 3b and RARβ. **C**) Total number of hydrogen bonds formed between compound 3a and RARγ

The hydrogen bond analysis illustrates the total number of hydrogen bonds formed between the compounds (**4a** and **3b)** and their respective RAR-α, RAR-β, and RAR-γ over the simulation course (Fig. 16). Hydrogen bonds play a crucial role in stabilizing protein–ligand interactions, and the number of hydrogen bonds formed can indicate the strength and stability of these interactions. Compound **4a** formed a stable and consistent hydrogen bond network with RAR-α, maintaining 3–6 hydrogen bonds throughout most of the simulation, which suggests a strong interaction contributing to the stability of the complex (Fig. 16a). Compound **3b** formed hydrogen bonds with RAR-β, between 0–2 bonds throughout the simulation (Fig. 16b), suggesting that the nature of the ligand binding pocket of RAR-β is slightly hydrophobic, and the interaction is mainly through hydrophobic interaction with weaker and more transient interaction through hydrogen bonds that are less frequently sustained over time. For compound **3b** with RAR-γ (Fig. 16c), the number of hydrogen bonds fluctuated between 0–3 bonds; this may indicate the same binding story as RAR-β. Overall, the current molecular dynamic simulation suggests that these compounds form more stable interactions with their cognate RARs, which matches with the molecular docking data and may correlate with conformational flexibility, making them potentially better candidates for RARs targeting therapeutic applications.

#### Structure–Activity Relationship

For the establishment of a preliminary structure–activity relationship (SAR) of the synthesized retinoid compounds, validation by redocking of ATRA and/or co-crystalized ligands in the RARs binding pocket was performed. The ATRA's observed conformation and/or original co-crystalized ligand showed superpositions in different binding pockets of RARs within the experimental crystal structures reported in the literature, demonstrating a generally acceptable match. It was noted that pockets of RAR-α and RAR-γ are matched thinner than that of RAR-β. However, there was a similar ligand length of the new synthetic retinoids to that of the ATRA molecule (~ 14 Å). The moderately twisted synthetic retinoid ligand conformations, the pocket's hydrophobic nature, and the tiny polar cluster at the end that binds to the retinoid carboxylate are among the structural characteristics of the RAR binding pocket. The aromatic rings in the middle of all synthetic structures are ideally positioned for hydrophobic interactions with the bottom of the pocket, while the substituents are tightly linked to the abundant hydrophobicity at the top of the pocket. The H12 moiety surrounds the pocket's entrance, trapping the retinoid within and interacting with its hydrophobic portion. In order to create a robust platform for the recruitment of coactivator peptides to the outer surface of H12, a strong hydrophobic interaction is necessary, and this was observed with all synthetic retinoid interactions. Near the polar cluster, the carboxylate is visible, enabling strong polar interactions with the conserved arginine and serine in this area. The current synthetic structures are ideal scaffolds for further development of new analogues for enhanced potency and selectivity.

## Conclusion

Four new ligands were synthesized as synthetic retinoids (**3a**, **3b**, **4a**, and **4b**) with potential dual activity as anticancer agents and intrinsic fluorescence probes for cellular imaging. The biological assessment demonstrated promising anti-proliferative activity against a wide range of cancer cell lines and potential minimal toxicity against normal fibroblast cells. The Caco-2 cell line was selected as the most sensitive cell. All compounds showed induction of cellular apoptosis at an early stage with a percentage of necrotic cells and cellular cycle arrest at the subG_0_-G_1_ phase. The new synthetic retinoid **4b** has the potential to modulate several key regulatory genes, such as *RARs*, *caspase-3*, *Cyt-C*, *CRABP I*, and *CRABP II*, and different RAR subtypes on the protein level. Also, new synthetic retinoids possess intracellular anti-inflammatory activity and can interfere with the ATP-catabolizing activity of calcium-independent ATPases with preferential activity to compound **4b**. Regarding the fluorescent characterization, the new compounds showed maximum emission at ~ 525 nm with preferential cellular fluorescence intensity for acid form compounds possibly due to the polarization effect in physiological pH. All compounds showed co-localization with Nile Red stain in non-polar regions, including lipid vesicles and nuclear and cell membranes suggesting their lipophilic property. The *in-silico* molecular docking suggested typical retinoid characteristics by best fitting the new ligands inside the ligand binding pocket of RAR subtypes through hydrogen bonding with Ser and Arg and various hydrophobic interactions. The molecular dynamic simulation supports complex stability within RARs binding pocket. This study was limited by lacking a full image of the drug response to realistic tumor microenvironment, tumor heterogeneity, stromal interactions, biodistribution, metabolism, immune response and so, transformation into *in-vivo* animal model (e.g., zebrafish) or ex-vivo (e.g., liver tissue, 3D cell culture) would validate the *in-vitro* results. Also, fluorescence can be affected by physiological pH, ionic strength, or quenching in different cellular compartments as well as intracellular photobleaching and phototoxicity and hence, the future optimization of photostability and brightness of compounds for long-term live-cell imaging is highly recommended. Despite these limitations and recommendations, the current *in-vitro* data findings provide valuable insights for rapidly developing class of new synthetic compounds with donor–acceptor fluorophore systems, which have potential anticancer activity and probes in cellular imaging investigations. This will open the gate to understand the intricate details of retinoid biological action, and their behaviors can be unlocked, therefore revealing the enormous therapeutic potential of both synthetic and natural retinoids.

## Supplementary Information

Below is the link to the electronic supplementary material.

Supplementary file1 (DOCX 254 KB)

Supplementary file2 (DOCX 654 KB)

Supplementary file3 (DOCX 1139 KB)

Supplementary file4 (DOCX 444 KB)

Supplementary file5 (JPG 881 KB)

Supplementary file6 (PDF 474 KB)

Supplementary file7 (PDF 639 KB)

## Data Availability

No datasets were generated or analysed during the current study.
