# Supplementary material for: New Fluorescent Synthetic Retinoids as Potential RAR Agonists with Anticancer, Molecular Docking and ADME Assessments
Source: J Fluoresc. 2025 May 23;35(11):11103–34. doi: 10.1007/s10895-025-04343-6 (PMC12718261; doi:10.1007/s10895-025-04343-6)
Supplement: Supplementary file 1 — Supplementary file1 (DOCX 254 KB) [file 10895_2025_4343_MOESM1_ESM.docx]

**New fluorescent synthetic retinoids as potential RAR agonists with anticancer, molecular docking and ADME assessments**

Esraa Ibrahim^1,2^, Yara E. Mansour^3^, Sameh Soror^1,2^ and Hesham Haffez^1,2*^

^1^Biochemistry and Molecular Biology Department, Faculty of Pharmacy, Helwan University, 11795, Cairo, Egypt.

^2^Center of Scientific Excellence “Helwan Structural Biology Research, (HSBR)”, Helwan University, 11795, Cairo, Egypt.

^3^Pharmaceutical Organic Chemistry Department, Faculty of Pharmacy, Helwan University, 11795, Cairo, Egypt.

* Corresponding authors:

Associate Prof. Hesham Haffez, [Hesham.haffez@pharm.helwan.edu.eg](mailto:Hesham.haffez@pharm.helwan.edu.eg), Biochemistry and Molecular Biology Department, Faculty of Pharmacy, Helwan University, 11795, Cairo, Egypt.


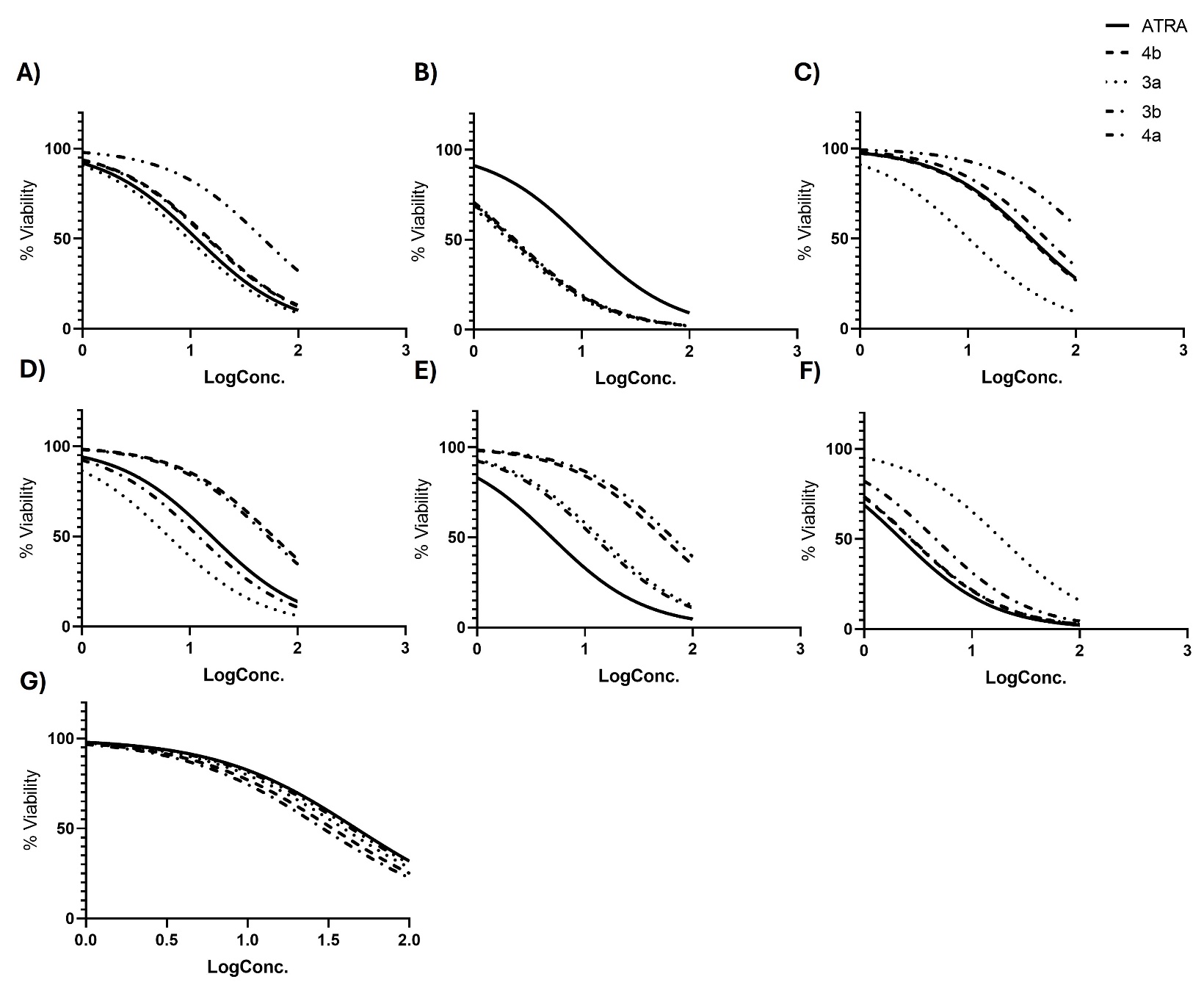


**Online resource 1** Cancer cell growth inhibition measured by MTT test. Sigmoidal dose-response curve of the investigated new synthetic fluorescent retinoids against seven cancer cell lines: A) Caco-2, B) MDA-MB231, C) HCT-116, D) HepG-2, E) MCF-7, F) PC-3 and G) WI-38. "Y-axes represents the percentage of cell viability (normalized to control), and X-axes represent the values of log [concentration]". The presented figures are representation of three replicates of the experiment.
