# Supplementary material for: New Fluorescent Synthetic Retinoids as Potential RAR Agonists with Anticancer, Molecular Docking and ADME Assessments
Source: J Fluoresc. 2025 May 23;35(11):11103–34. doi: 10.1007/s10895-025-04343-6 (PMC12718261; doi:10.1007/s10895-025-04343-6)
Supplement: Supplementary file 3 — Supplementary file3 (DOCX 1139 KB) [file 10895_2025_4343_MOESM3_ESM.docx]

**New fluorescent synthetic retinoids as potential RAR agonists with anticancer, molecular docking and ADME assessments**

Esraa Ibrahim^1,2^, Yara E. Mansour^3^, Sameh Soror^1,2^ and Hesham Haffez^1,2*^

^1^Biochemistry and Molecular Biology Department, Faculty of Pharmacy, Helwan University, 11795, Cairo, Egypt.

^2^Center of Scientific Excellence “Helwan Structural Biology Research, (HSBR)”, Helwan University, 11795, Cairo, Egypt.

^3^Pharmaceutical Organic Chemistry Department, Faculty of Pharmacy, Helwan University, 11795, Cairo, Egypt.

* Corresponding authors:

Associate Prof. Hesham Haffez, [Hesham.haffez@pharm.helwan.edu.eg](mailto:Hesham.haffez@pharm.helwan.edu.eg), Biochemistry and Molecular Biology Department, Faculty of Pharmacy, Helwan University, 11795, Cairo, Egypt.


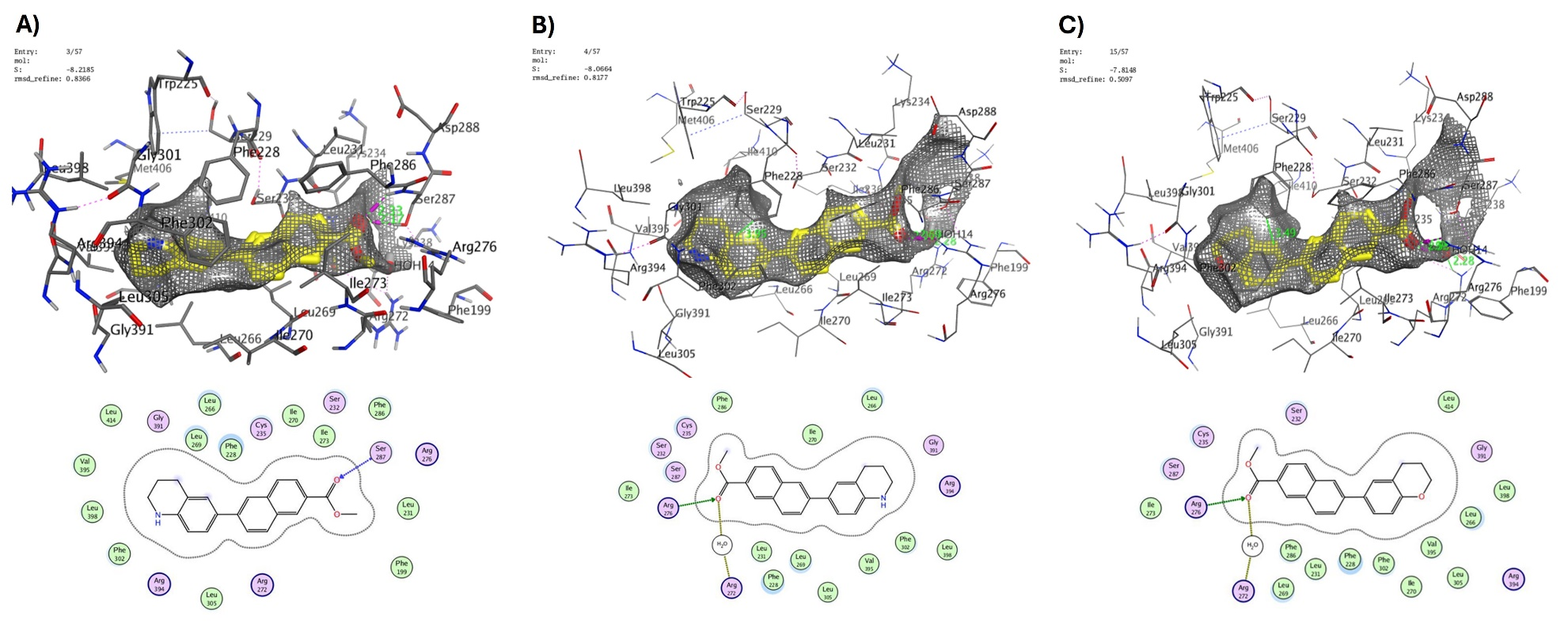


**Online resource 3** Represented the 3D and 2D of the binding modes of **A) compound 3a**, **B)** **compound 4b** and **C)** **compound 3b** in binding pocket of RAR-α
