# Supplementary figures and images for: New Fluorescent Synthetic Retinoids as Potential RAR Agonists with Anticancer, Molecular Docking and ADME Assessments
Source: J Fluoresc. 2025 May 23;35(11):11103–34. doi: 10.1007/s10895-025-04343-6 (PMC12718261; doi:10.1007/s10895-025-04343-6)

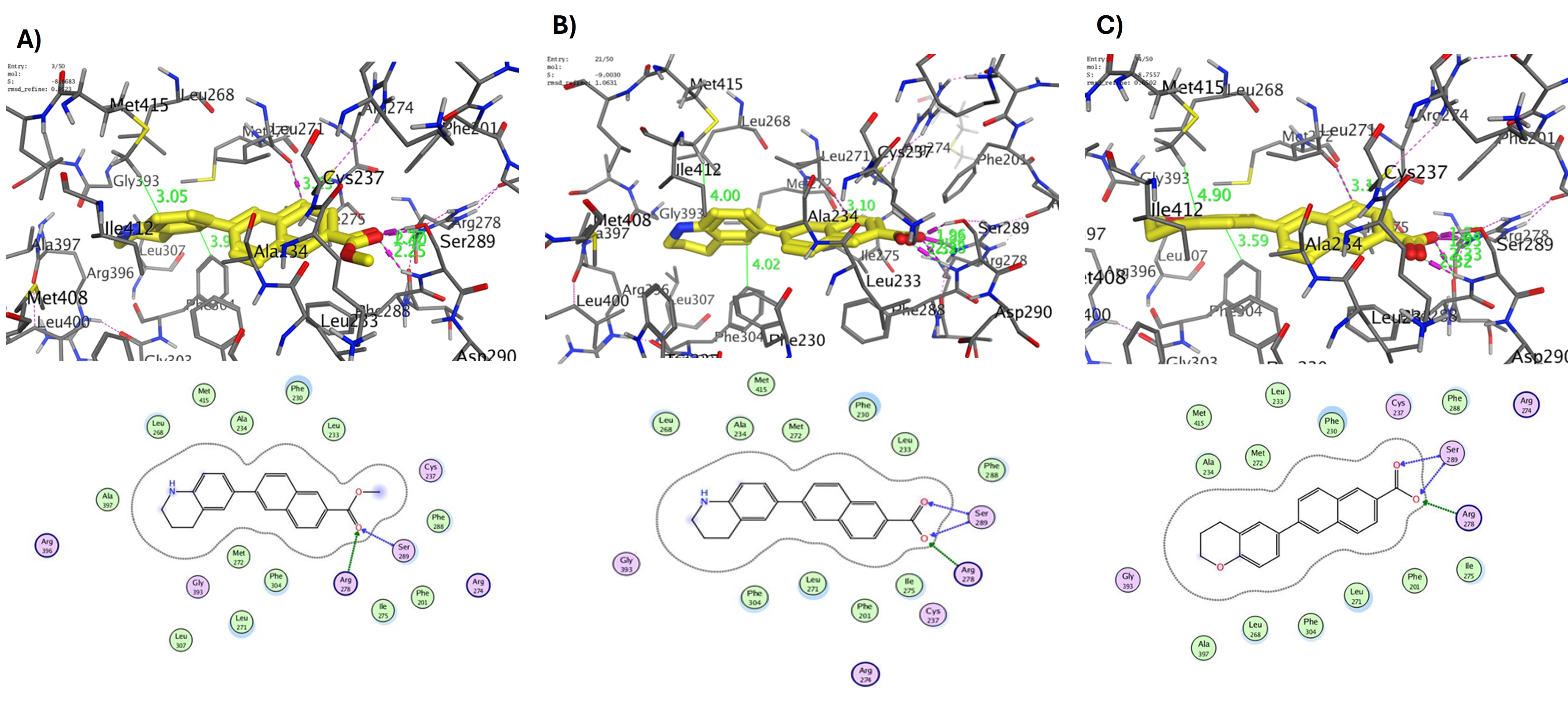

Supplement: Supplementary file 5 — Supplementary file5 (JPG 881 KB) [file 10895_2025_4343_MOESM5_ESM.jpg]
