# Supplementary material for: New Fluorescent Synthetic Retinoids as Potential RAR Agonists with Anticancer, Molecular Docking and ADME Assessments
Source: J Fluoresc. 2025 May 23;35(11):11103–34. doi: 10.1007/s10895-025-04343-6 (PMC12718261; doi:10.1007/s10895-025-04343-6)
Supplement: Supplementary file 6 — Supplementary file6 (PDF 474 KB) [file 10895_2025_4343_MOESM6_ESM.pdf]

Supplementary figures for New fluorescent synthetic retinoids as potential RAR agonists with anticancer, molecular docking and ADME assessments

Esraa Ibrahim[1,2], Yara E. Mansour[3], Sameh Soror[1,2] and Hesham Haffez[1,2*]

[1]Biochemistry and Molecular Biology Department, Faculty of Pharmacy, Helwan University, 11795, Cairo, Egypt.

[2]Center of Scientific Excellence "Helwan Structural Biology Research, (HSBR)", Helwan University, 11795, Cairo, Egypt.

[3]Pharmaceutical Organic Chemistry Department, Faculty of Pharmacy, Helwan University, 11795, Cairo, Egypt.

* Corresponding authors:

Associate Prof. Hesham Haffez, Hesham.haffez@pharm.helwan.edu.eg, Biochemistry and Molecular Biology Department, Faculty of Pharmacy, Helwan University, 11795, Cairo, Egypt.

A) B)

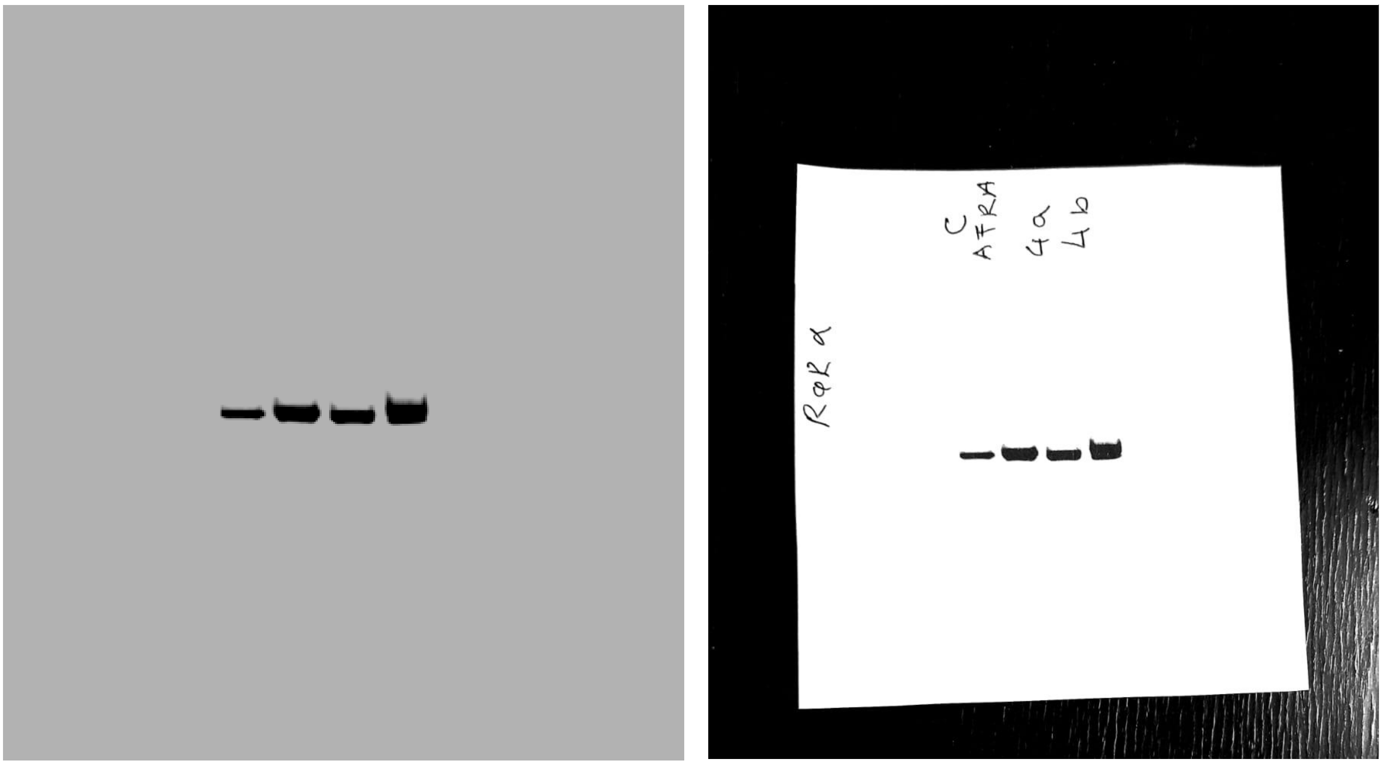

Fig. 2S: Original western blot for RARα proein expression level for Caco-2 cancer cell line samples showing **A)** original western blott image and **B)** original western blot membrane. (C: negative control, ATRA, 4a and 4b).

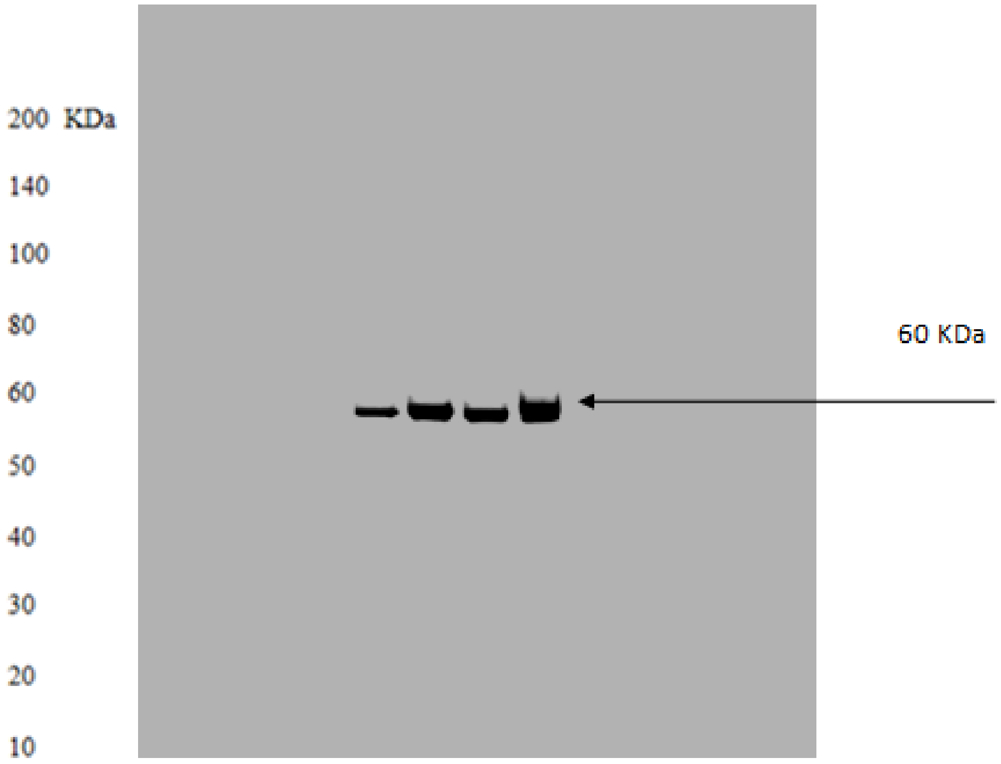

Fig. 3S: Original western blot for RARα proein expression level for Caco-2 cancer cell line samples showing the actual molecular weight of RARα proein.

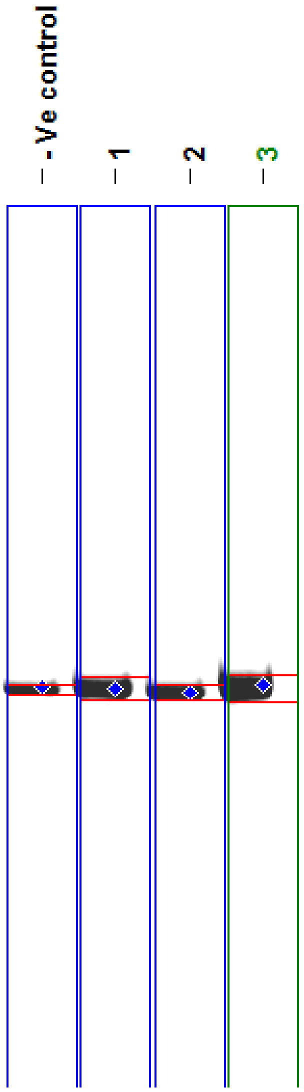

Fig. 4S: Computerized detection of RARα proein expression level for Caco-2 cancer cell line samples. (-ve control, lane 1: ATRA, lane 2: 4a and lane 3: 4b). Gel documentation system (Geldoc-it, UVP, England) was applied for data analysis using Totallab analysis software, ww.totallab.com, (Ver.1.0.1).

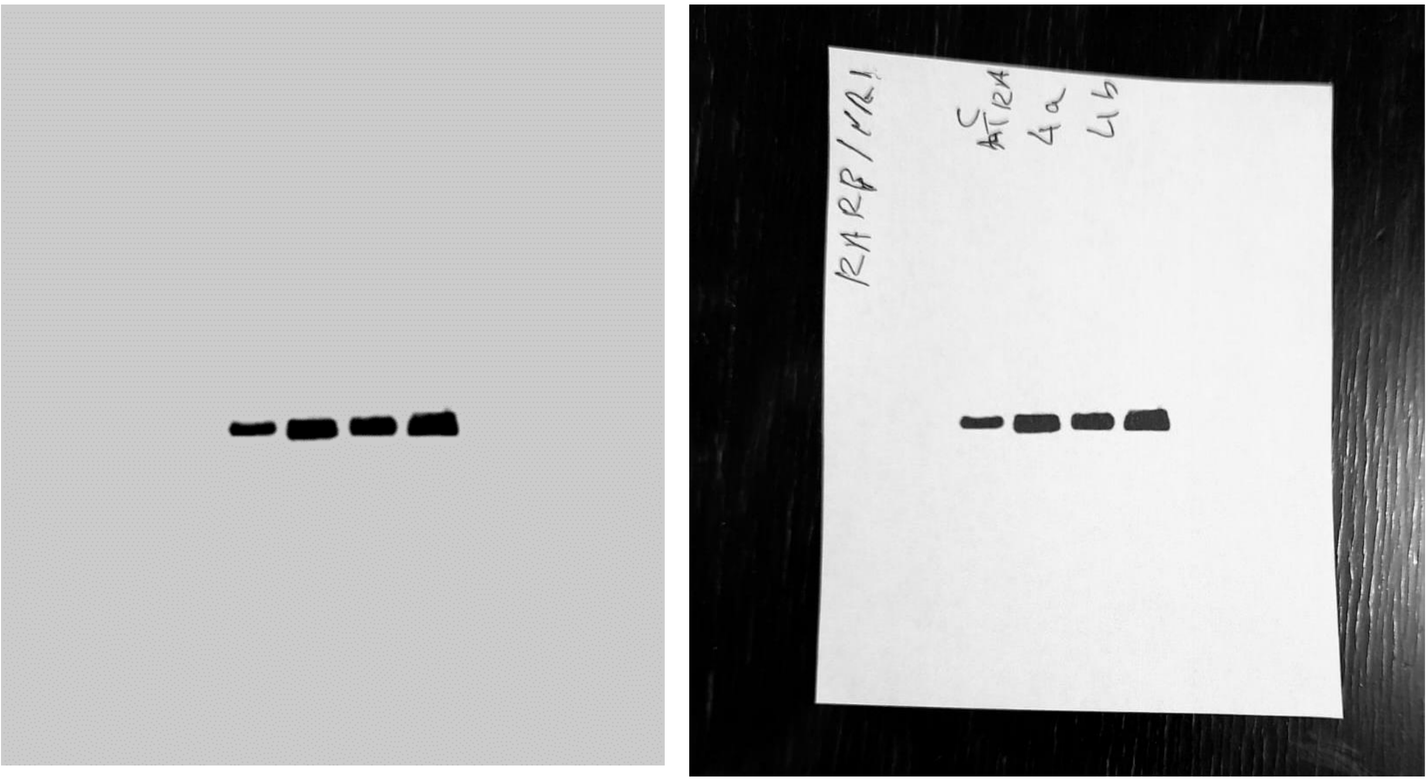

Fig. 5S: Original western blot for RARβ proein expression level for Caco-2 cancer cell line samples showing **A)** original western blott image and **B)** original western blot membrane. (C: negative control, ATRA, 4a and 4b).

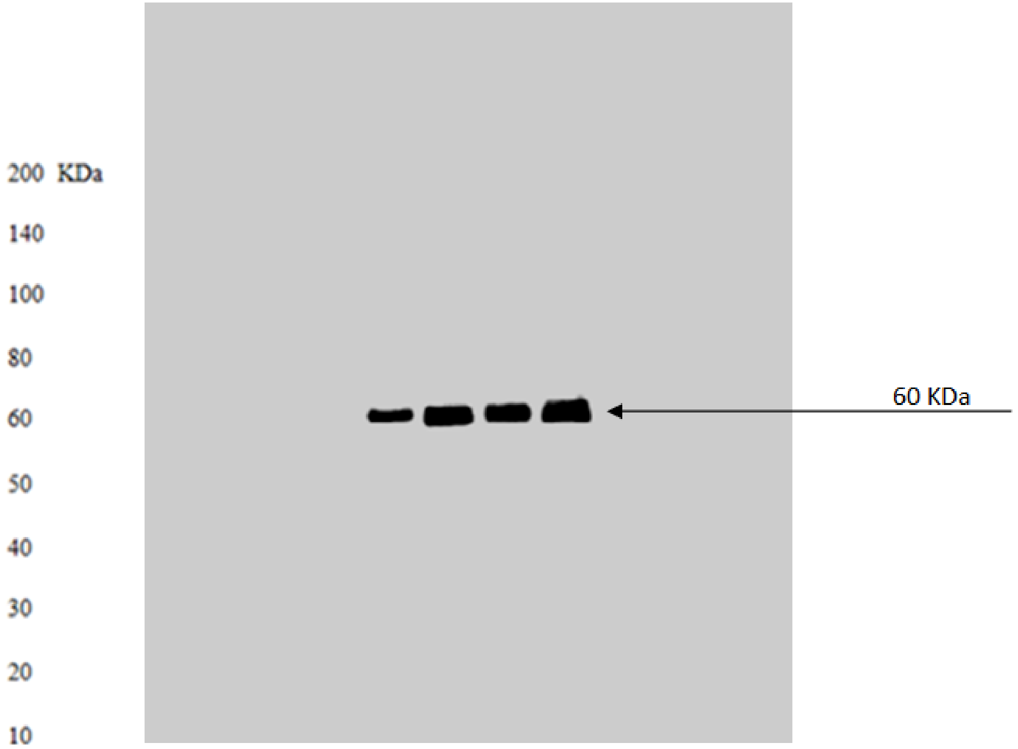

Fig. 6S: Original western blot for RARβ proein expression level for Caco-2 cancer cell line samples showing the actual molecular weight of RARβ proein.

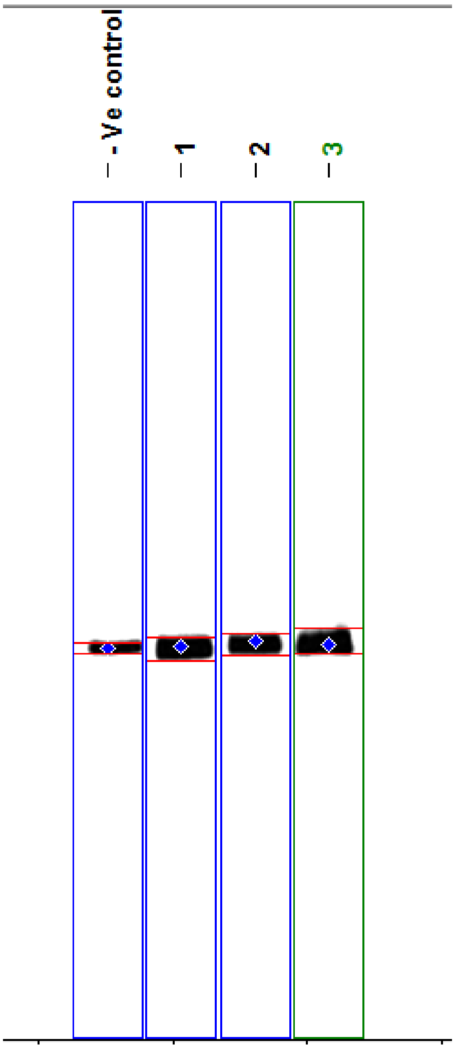

Fig. 7S: Computerized detection of RARβ proein expression level for Caco-2 cancer cell line samples. (-ve control, lane 1: ATRA, lane 2: 4a and lane 3: 4b). Gel documentation system (Geldoc-it, UVP, England) was applied for data analysis using Totallab analysis software, ww.totallab.com, (Ver.1.0.1).

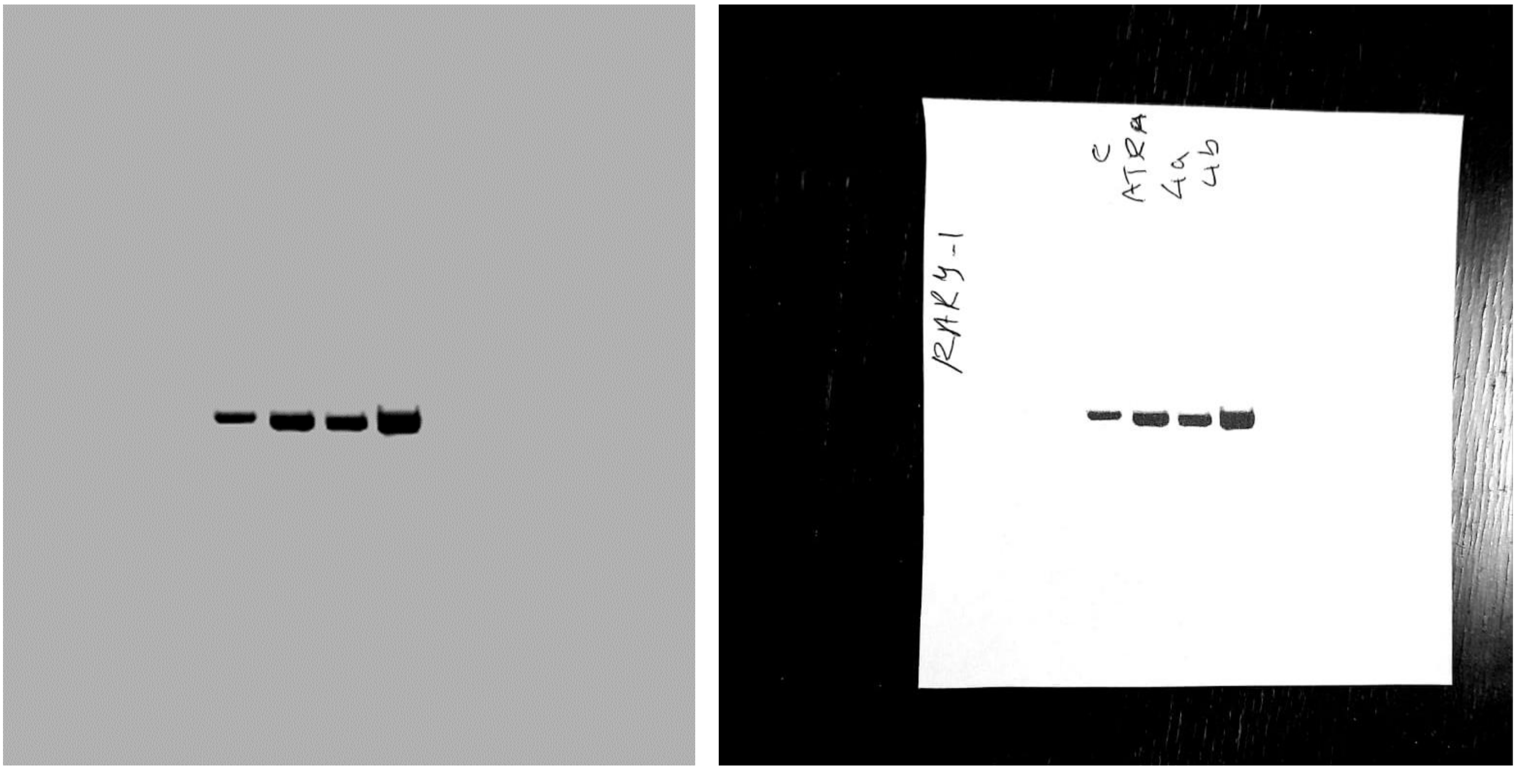

Fig. 8S: Original western blot for RARγ proein expression level for Caco-2 cancer cell line samples showing **A)** original western blott image and **B)** original western blot membrane. (C: negative control, ATRA, 4a and 4b).

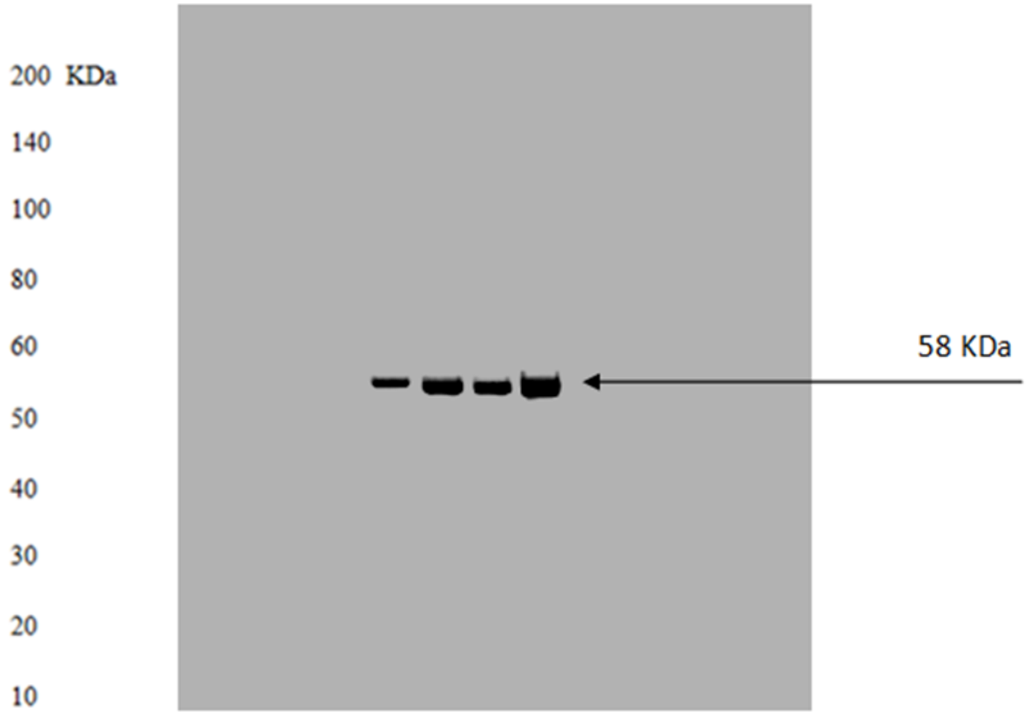

Fig. 9S: Original western blot for RARγ proein expression level for Caco-2 cancer cell line samples showing the actual molecular weight of RARγ proein.

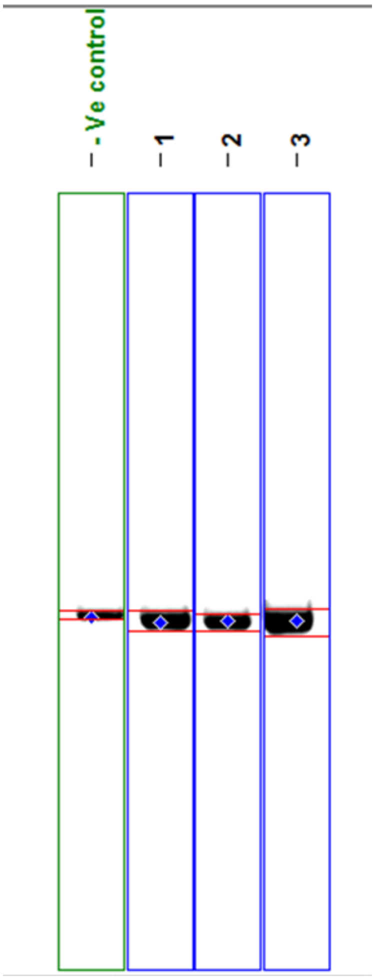

Fig. 10S: Computerized detection of RARγ proein expression level for Caco-2 cancer cell line samples. (-ve control, lane 1: ATRA, lane 2: 4a and lane 3: 4b). Gel documentation system (Geldoc-it, UVP, England) was applied for data analysis using Totallab analysis software, ww.totallab.com, (Ver.1.0.1).

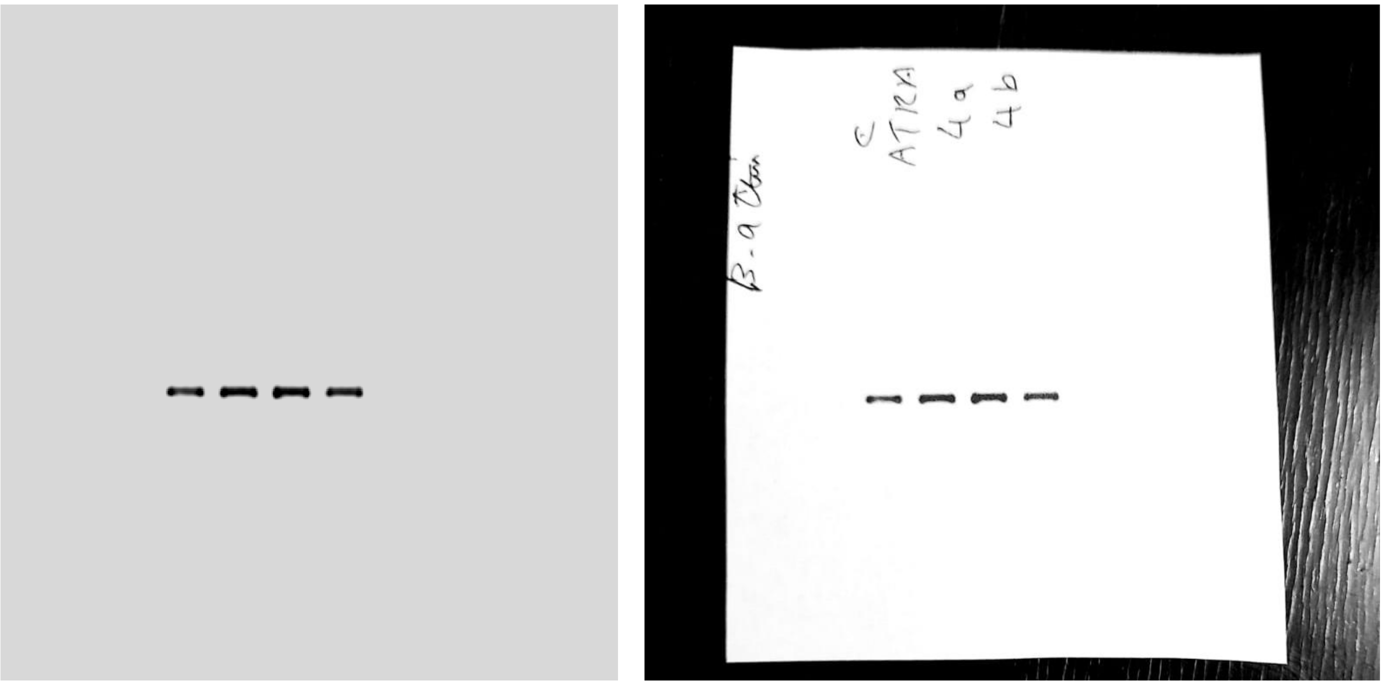

Fig. 11S: Original western blot for β-actin proein expression level for Caco-2 cancer cell line samples showing **A)** original western blott image and **B)** original western blot membrane. (C: negative control, ATRA, 4a and 4b).

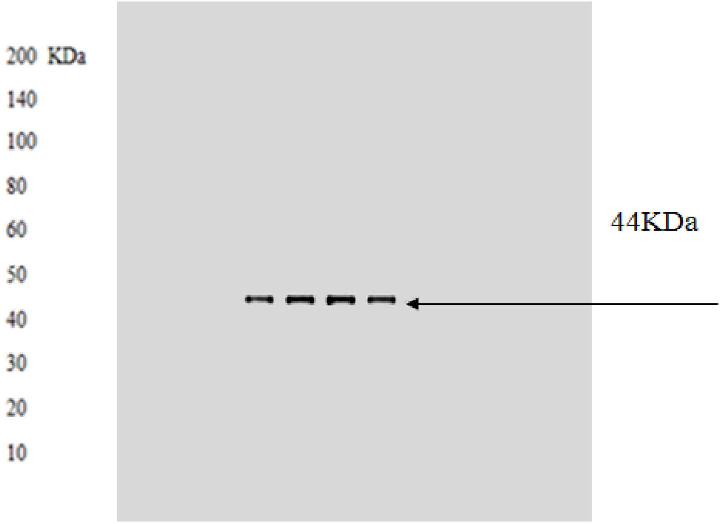

Fig. 12S: Original western blot for β-actin proein expression level for Caco-2 cancer cell line samples showing the actual molecular weight of β-actin proein.

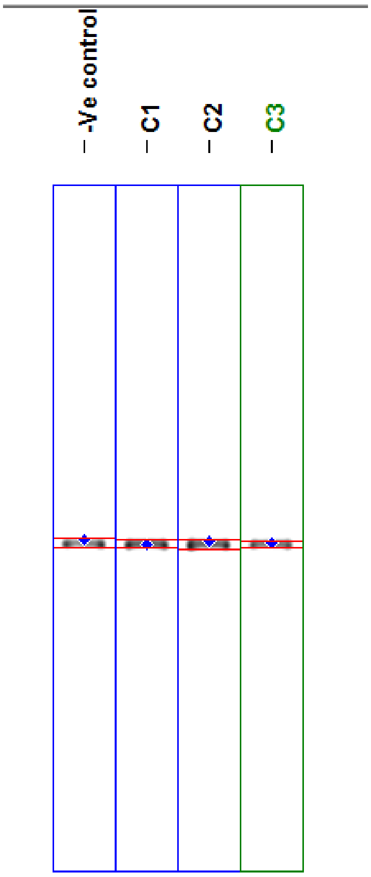

Fig. 13S: Computerized detection of β-actin proein expression level for Caco-2 cancer cell line samples. (-ve control, lane 1: ATRA, lane 2: 4a and lane 3: 4b). Gel documentation system (Geldoc-it, UVP, England) was applied for data analysis using Totallab analysis software, ww.totallab.com, (Ver.1.0.1).
