# Supplementary material for: New Fluorescent Synthetic Retinoids as Potential RAR Agonists with Anticancer, Molecular Docking and ADME Assessments
Source: J Fluoresc. 2025 May 23;35(11):11103–34. doi: 10.1007/s10895-025-04343-6 (PMC12718261; doi:10.1007/s10895-025-04343-6)

yara-essam-YR72P

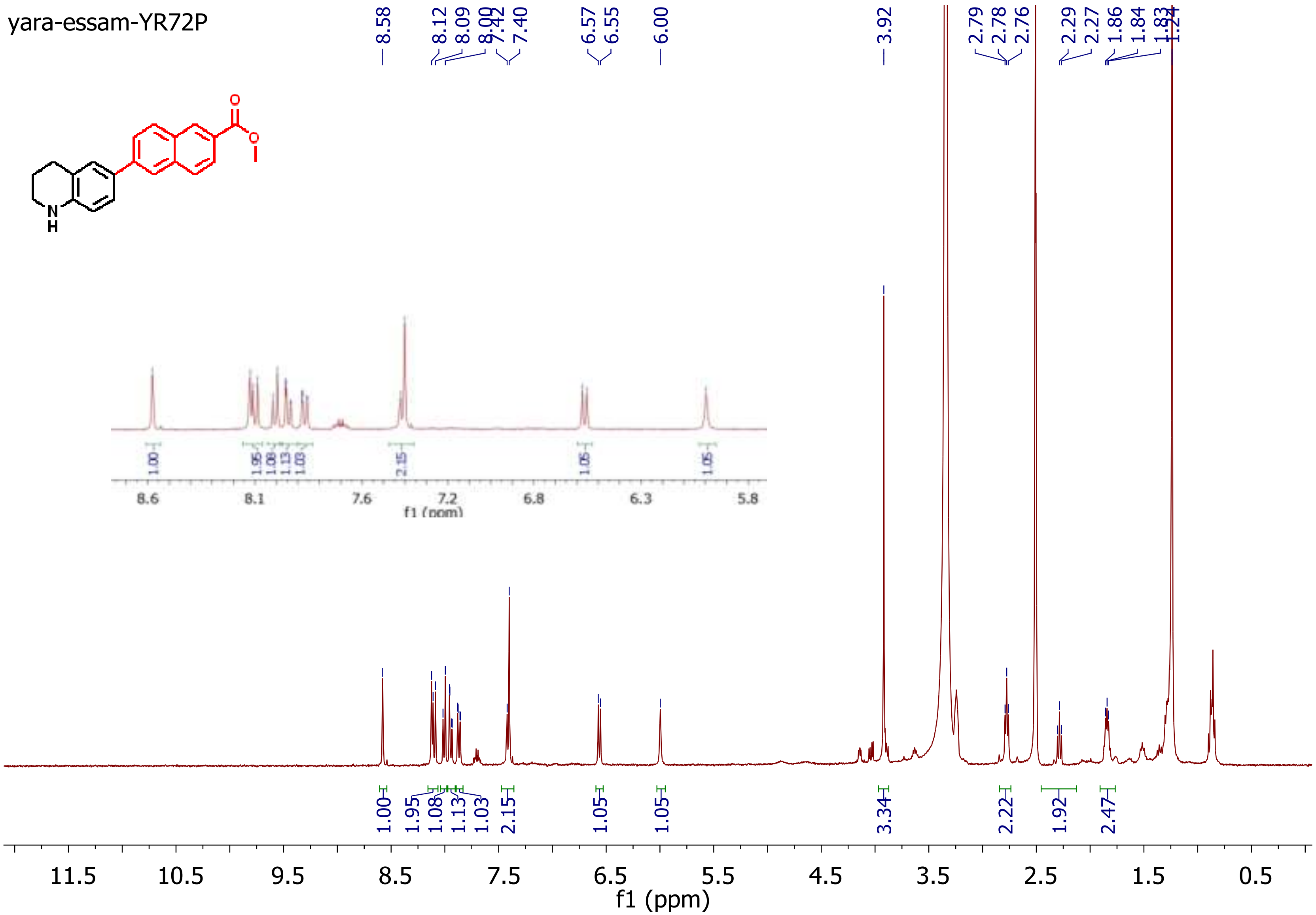

yara-essam-YR72P-d2o

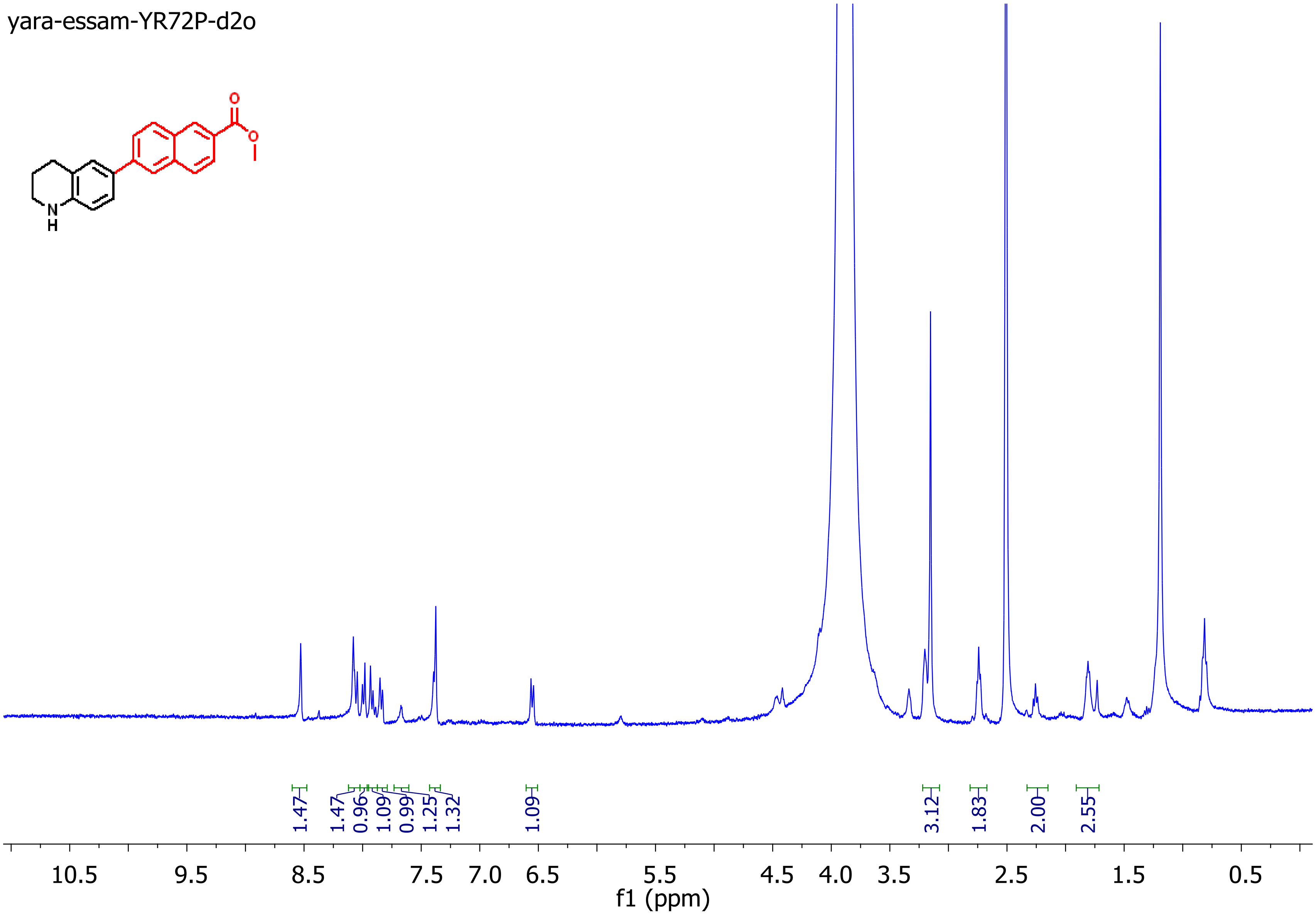

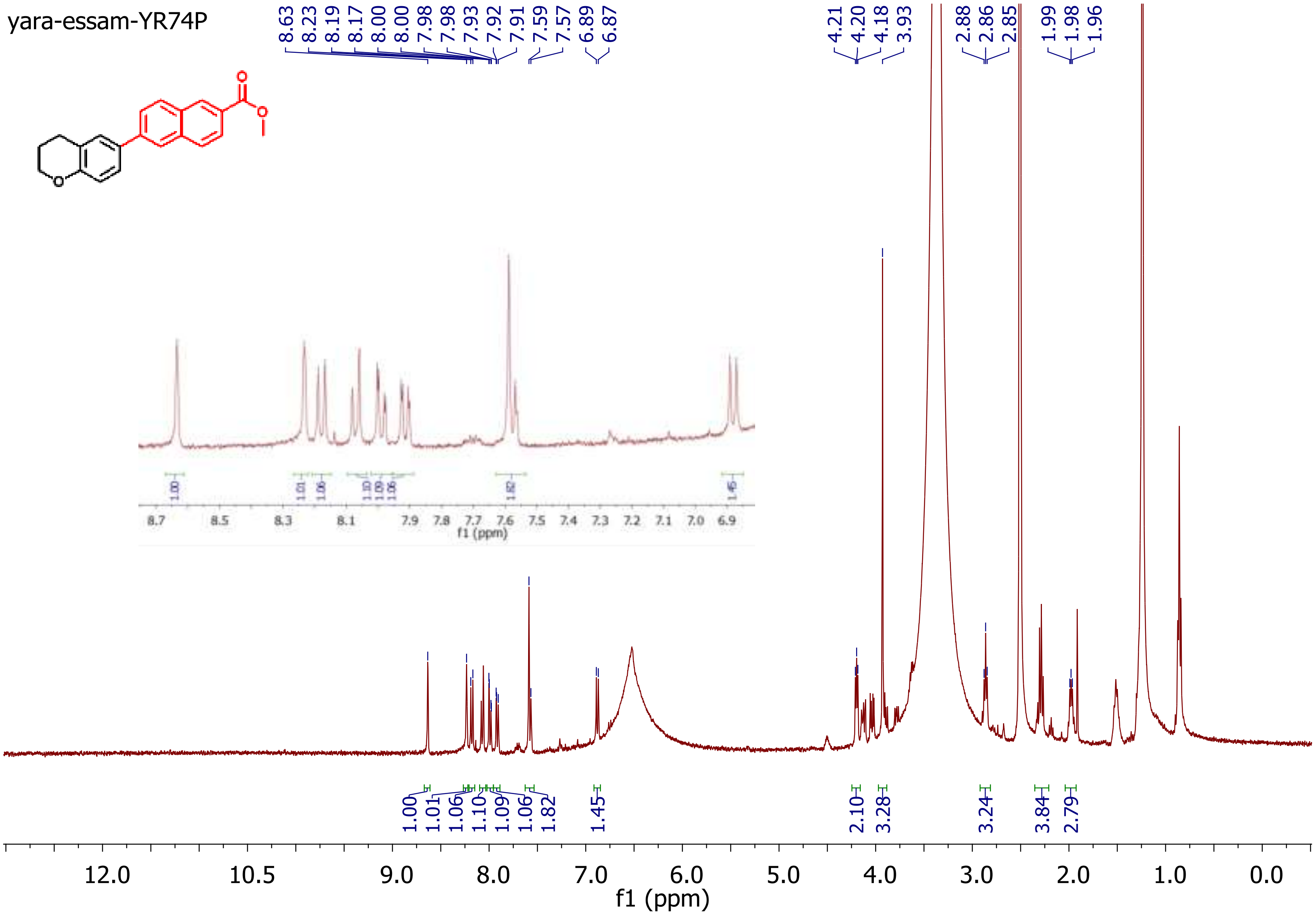

yara-essam-YR74P
8.63 8.23 8.19 8.17 8.00 8.00 7.98 7.98 7.93 7.92 7.91 7.59 7.57 6.89 6.87
4.21 4.20 4.18 3.93
2.88 2.86 2.85
1.99 1.98 1.96
1.00 1.01 1.06 1.10 1.09 1.06 1.82 1.45
2.10 3.28 3.24 3.84 2.79
12.0 10.5 9.0 8.0 7.0 6.0 5.0 4.0 3.0 2.0 1.0 0.0
f1 (ppm)

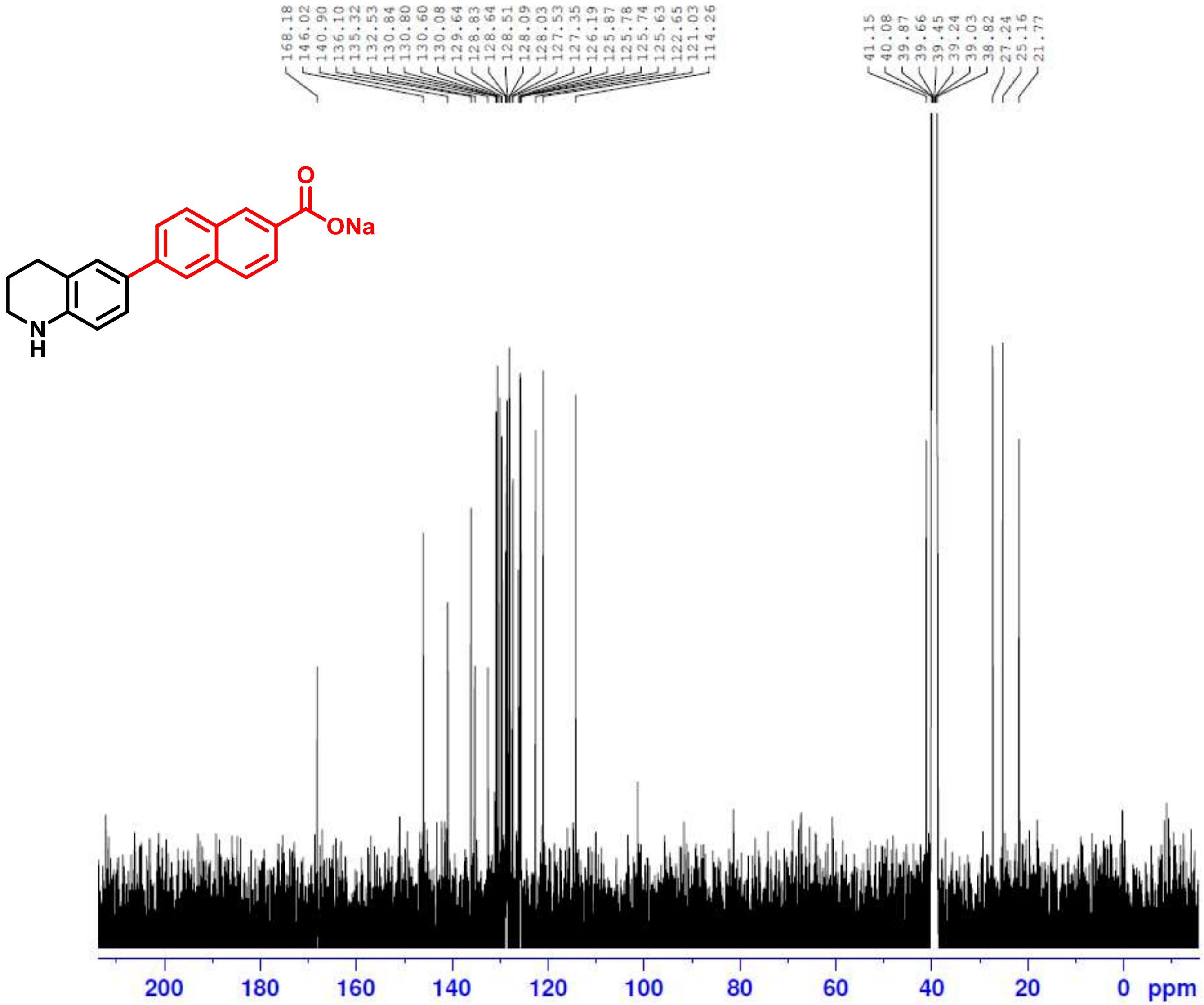

BRUKER

```
Current Data Parameters
NAME        yara-essam-YR-72
EXPNO                      2
PROCNO                     1

F2 - Acquisition Parameters
Date_               20230405
Time                   16.47
INSTRUM                spect
PROBHD     5 mm PABBO BB/
PULPROG              zgpg30
TD                     65536
SOLVENT                 DMSO
NS                      8196
DS                         4
SWH             24038.461 Hz
FIDRES           0.366798 Hz
AQ              1.3631488 sec
RG                    205.37
DW                20.800 usec
DE                  6.50 usec
TE                   300.0 K
D1             2.00000000 sec
D11            0.03000000 sec
TD0                        1

======== CHANNEL f1 ========
SFO1         100.6278588 MHz
NUC1                     13C
P1                 10.00 usec
PLW1          47.00000000 W

======== CHANNEL f2 ========
SFO2         400.1516006 MHz
NUC2                      1H
CPDPRG[2             waltz16
PCPD2              90.00 usec
PLW2          18.00000000 W
PLW12          0.34722000 W
PLW13          0.28125000 W

F2 - Processing parameters
SI                     32768
SF           100.6177975 MHz
WDW                       EM
SSB       0
LB                   1.00 Hz
GB        0
PC                      1.40
```

Peak ID	Time	Error PPM
19	11.34	

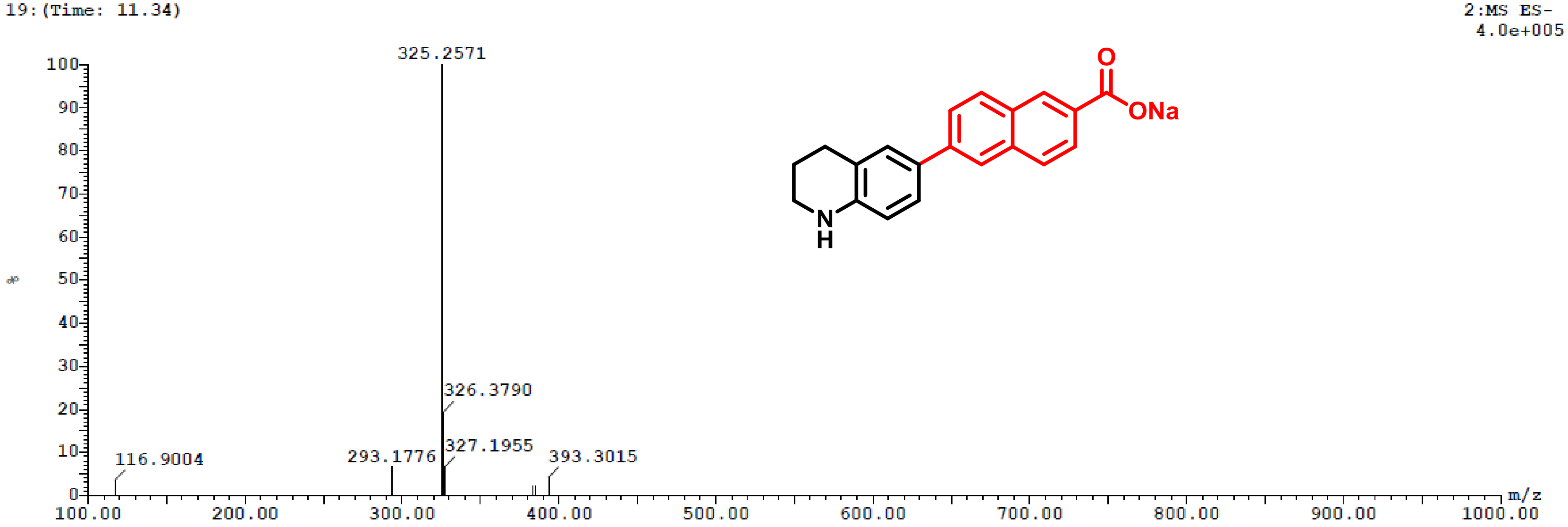

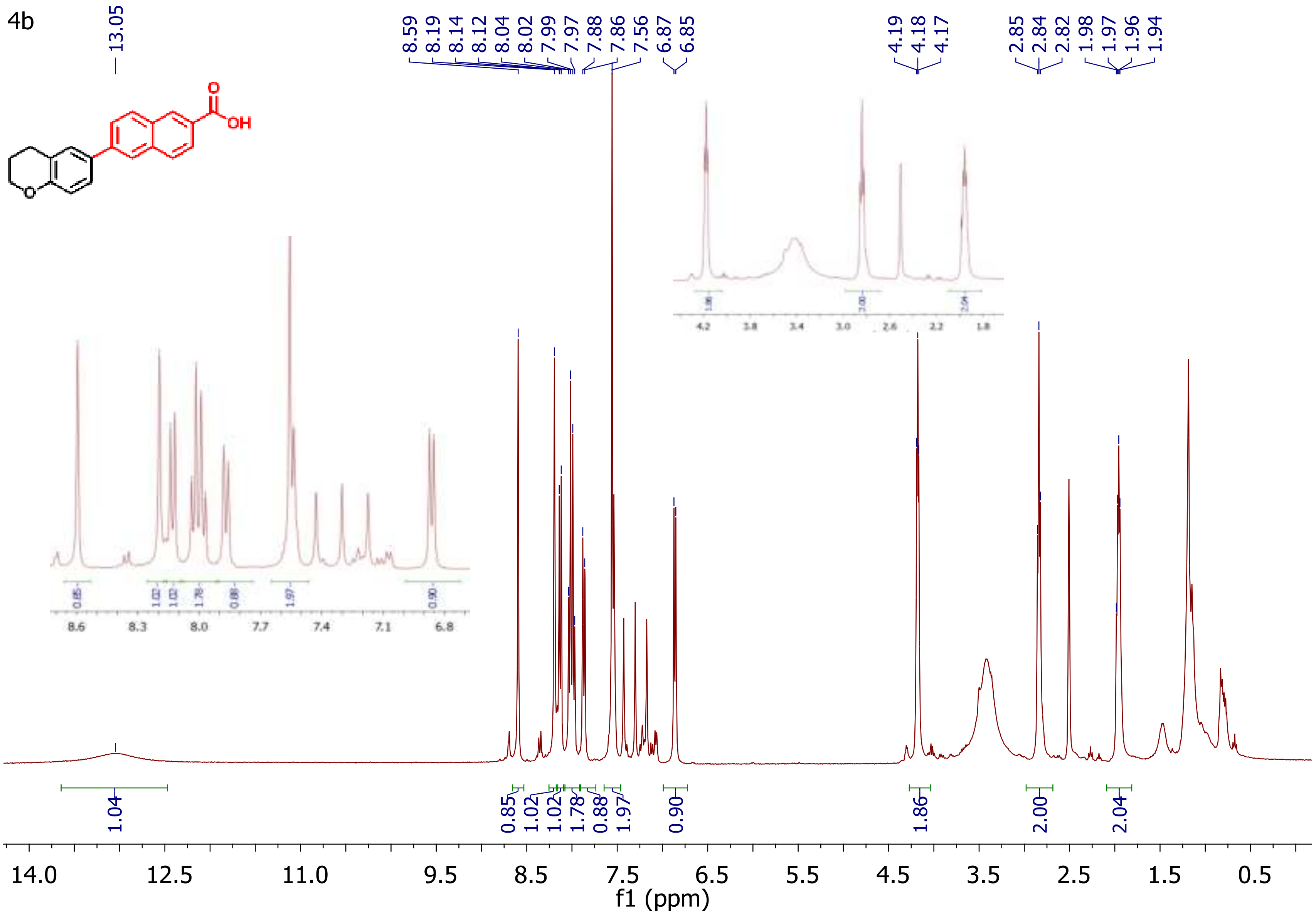
4b
13.05
8.59 8.19 8.14 8.12 8.04 8.02 7.99 7.97 7.88 7.86 7.56 6.87 6.85
4.19 4.18 4.17
2.85 2.84 2.82 1.98 1.97 1.96 1.94
OH
O
1.04
0.85 1.02 1.02 1.78 0.88 1.97
0.90
1.86
2.00
2.04
14.0 12.5 11.0 9.5 8.5 7.5 6.5 5.5 4.5 3.5 2.5 1.5 0.5
f1 (ppm)

4b + D2O

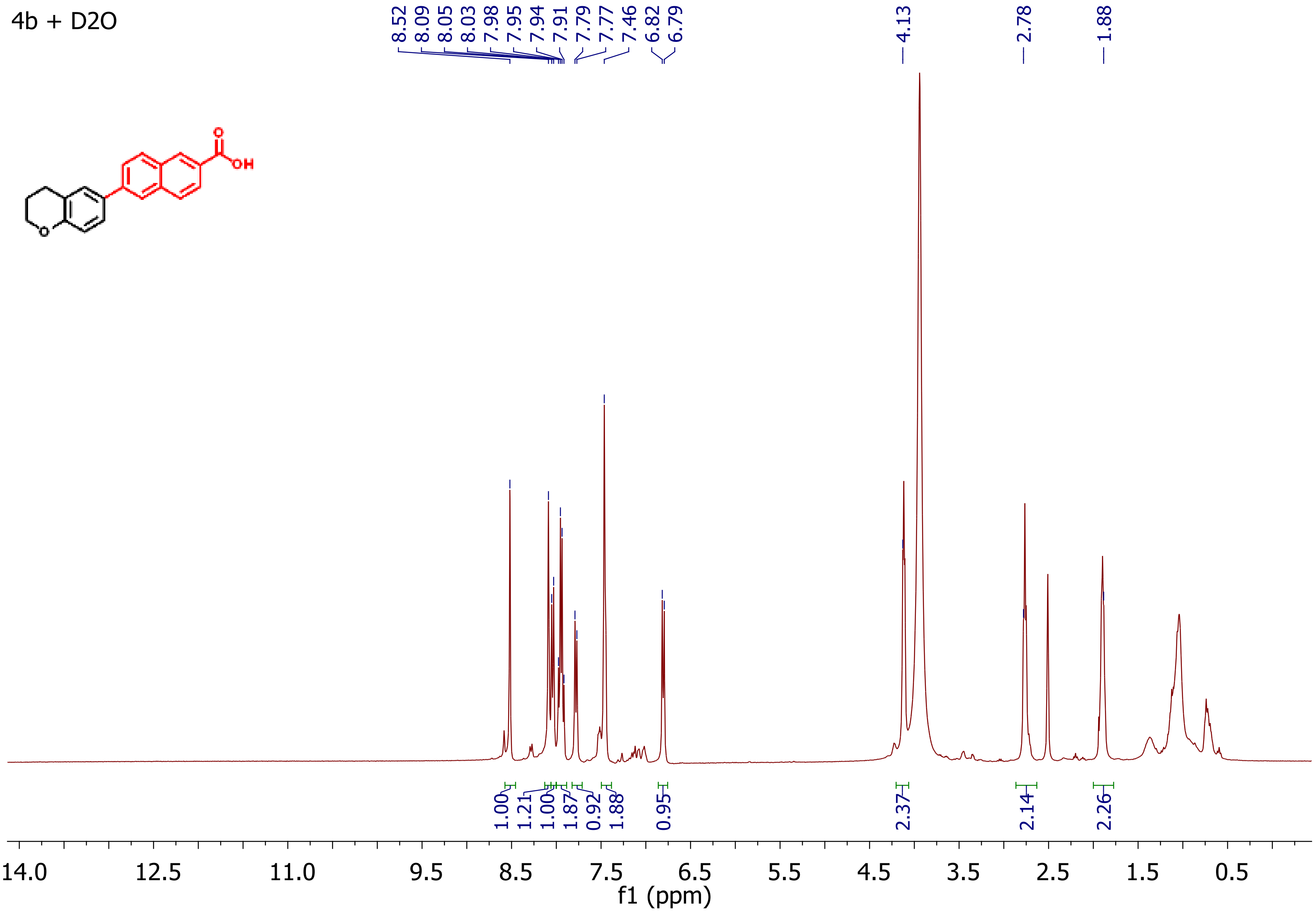

8.52
8.09
8.05
8.03
7.98
7.95
7.94
7.91
7.79
7.77
7.46
6.82
6.79
4.13
2.78
1.88
O
OH
O
1.00
1.21
1.00
1.87
0.92
1.88
0.95
2.37
2.14
2.26
14.0
12.5
11.0
9.5
8.5
7.5
6.5
5.5
4.5
3.5
2.5
1.5
0.5
f1 (ppm)

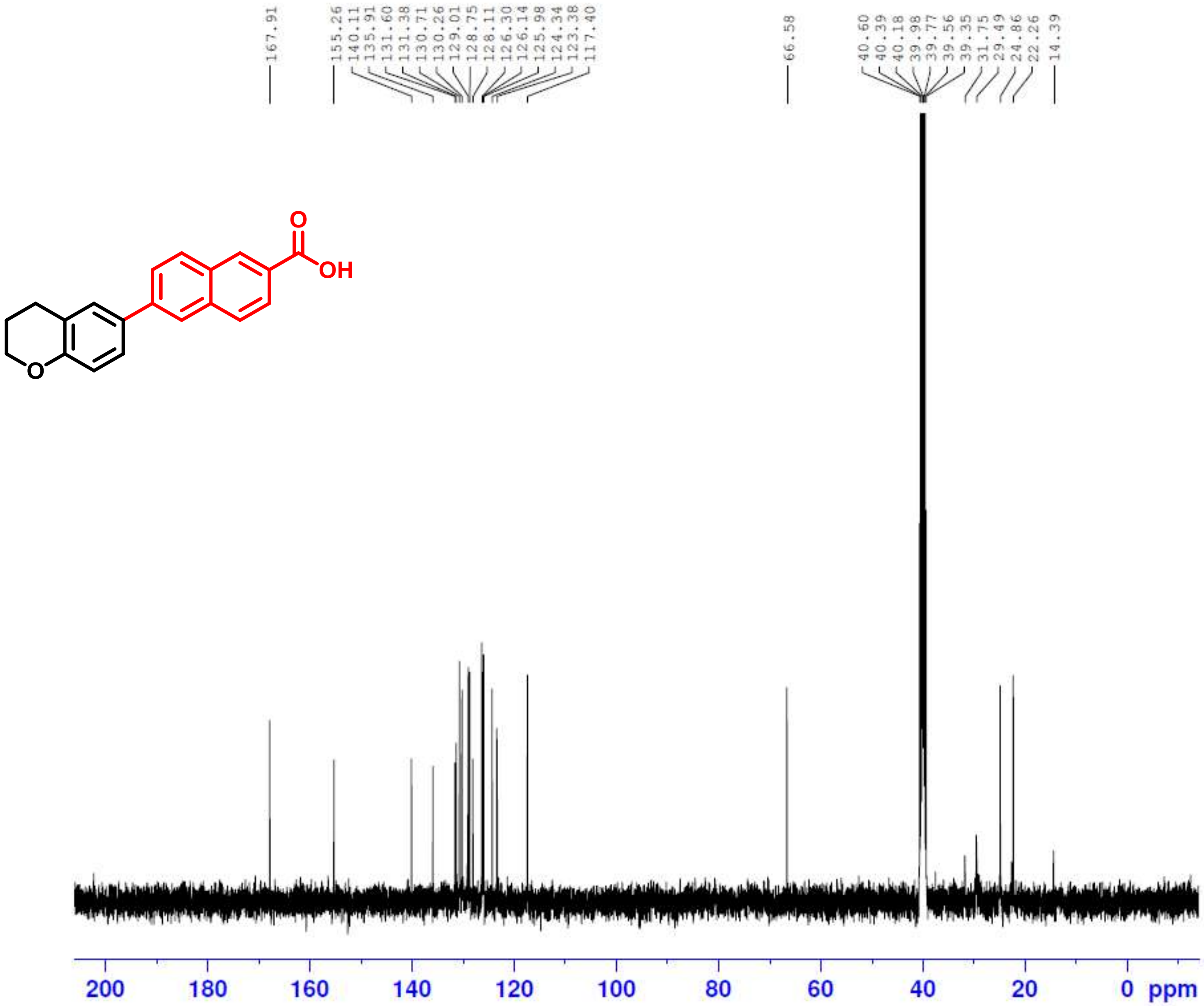


```
Current Data Parameters
NAME       yara-essam-YR67
EXPNO                    2
PROCNO                   1

F2 - Acquisition Parameters
Date_             20230731
Time                 12.56
INSTRUM              spect
PROBHD    5 mm PABBO BB/
PULPROG            zgpg30
TD                  65536
SOLVENT              DMSO
NS                    120
DS                      4
SWH             24038.461 Hz
FIDRES           0.366798 Hz
AQ              1.3631488 sec
RG                 205.37
DW                 20.800 usec
DE                   6.50 usec
TE                  300.0 K
D1             2.00000000 sec
D11            0.03000000 sec
TD0                     1

======== CHANNEL f1 ========
SFO1         100.6278588 MHz
NUC1                 13C
P1                 10.00 usec
PLW1         47.00000000 W

======== CHANNEL f2 ========
SFO2         400.1516006 MHz
NUC2                  1H
CPDPRG[2         waltz16
PCPD2              90.00 usec
PLW2         18.00000000 W
PLW12         0.34722000 W
PLW13         0.28125000 W

F2 - Processing parameters
SI                 32768
SF           100.6177975 MHz
WDW                   EM
SSB       0
LB                  1.00 Hz
GB        0
PC                  1.40
```

Peak ID **Time** **Error PPM**
11 8.95

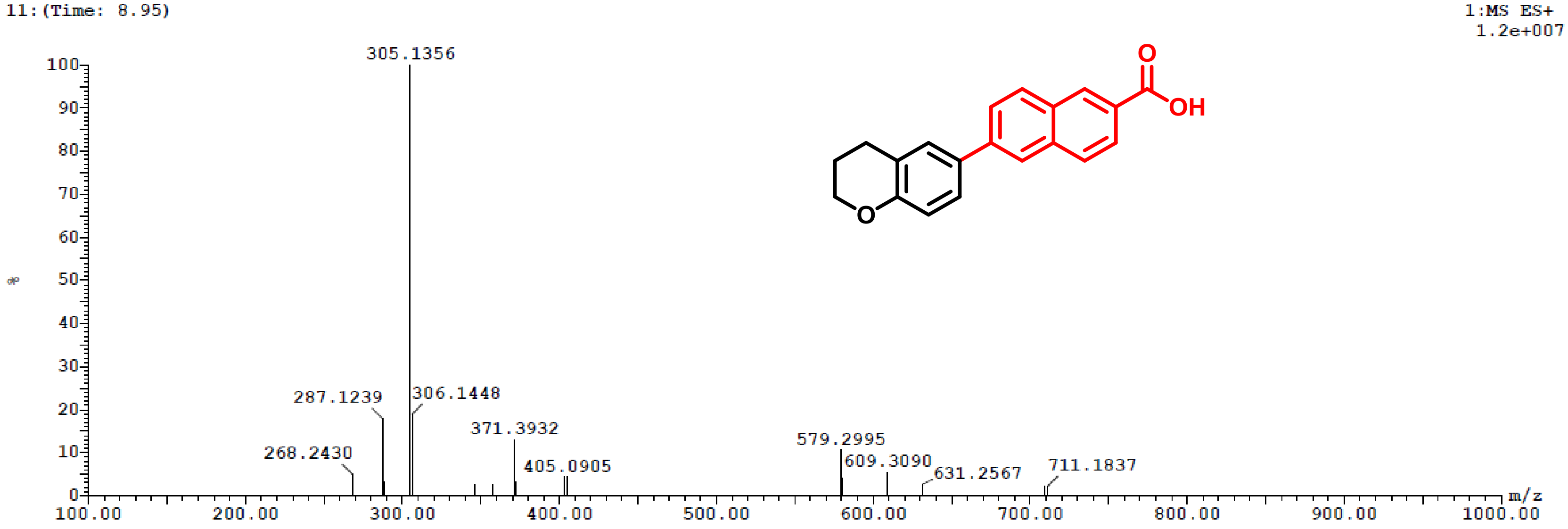

Supplement: Supplementary file 7 — Supplementary file7 (PDF 639 KB) [file 10895_2025_4343_MOESM7_ESM.pdf]
